# Stellate Cells in the Medial Entorhinal Cortex Are Required for Spatial Learning

**DOI:** 10.1016/j.celrep.2018.01.005

**Published:** 2018-01-30

**Authors:** Sarah A. Tennant, Lukas Fischer, Derek L.F. Garden, Klára Zsófia Gerlei, Cristina Martinez-Gonzalez, Christina McClure, Emma R. Wood, Matthew F. Nolan

**Affiliations:** 1 Centre for Discovery Brain Sciences, University of Edinburgh, Edinburgh EH8 9XD, UK

**Keywords:** spatial cognition, learning, memory, neural computation, location estimation, cue-based navigation, path integration, entorhinal cortex, virtual reality, behavior

## Abstract

Spatial learning requires estimates of location that may be obtained by path integration or from positional cues. Grid and other spatial firing patterns of neurons in the superficial medial entorhinal cortex (MEC) suggest roles in behavioral estimation of location. However, distinguishing the contributions of path integration and cue-based signals to spatial behaviors is challenging, and the roles of identified MEC neurons are unclear. We use virtual reality to dissociate linear path integration from other strategies for behavioral estimation of location. We find that mice learn to path integrate using motor-related self-motion signals, with accuracy that decreases steeply as a function of distance. We show that inactivation of stellate cells in superficial MEC impairs spatial learning in virtual reality and in a real world object location recognition task. Our results quantify contributions of path integration to behavior and corroborate key predictions of models in which stellate cells contribute to location estimation.

## Highlights

• Mice learn to estimate location by path integration and cue-based strategies
• Motor-related self-motion signals are used for path integration
• Accuracy of path integration decreases with distance
• Stellate cells in medial entorhinal cortex are required for spatial learning

## Graphical Abstract

**Figure:**
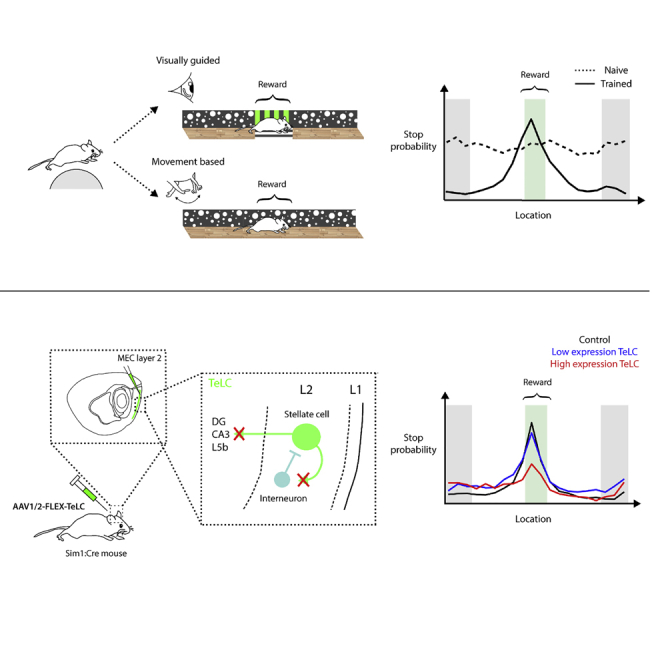

## Teaser

Tennant et al. develop virtual reality tasks that dissociate beaconing and path integration strategies for location estimation. In combination with genetically targeted inactivation of synaptic output, the authors provide evidence for a critical role for entorhinal stellate cells in spatial learning.

## Introduction

The ability to learn and update estimates of location during movement is central to theories of animal and artificial navigation (Durrant-Whyte and Bailey, 2006, McNaughton et al., 1996, McNaughton et al., 2006). In mammals, this core cognitive function may be achieved either using spatial cues, for example, through triangulation or beaconing strategies (Geva-Sagiv et al., 2015), or by path integration mechanisms, which generate representations of location from information about direction and speed of movement (Etienne and Jeffery, 2004). However, behavioral dissociation of path integration from cue-based navigation is challenging, as for many spatial behaviors investigated experimentally location estimates generated by any of several possible strategies may be sufficient for successful task performance. Indeed, while elegant experimental manipulations have directly tested mechanisms and roles of path integration in invertebrates (Collett et al., 1998, Collett et al., 2013, Wittlinger et al., 2006), the extent to which mammals use path integration strategies behaviorally is unclear, and whether the underlying neural substrates differ from those for cue-based location estimation is not known (Etienne and Jeffery, 2004, Jacob et al., 2017, Van Cauter et al., 2013, Winter et al., 2013).

The medial entorhinal cortex (MEC) contains multiple functional cell types that generate spatial representations that may be well suited to support behavioral estimation of location (Diehl et al., 2017, Hafting et al., 2005, Hardcastle et al., 2017, Solstad et al., 2008). These functional cell types include grid cells, which encode location through repeating hexagonally arranged firing fields (Hafting et al., 2005). Within the MEC, layer 2 has the greatest density of neurons with grid-firing fields (Sargolini et al., 2006), and grid firing has been localized to excitatory neurons with stellate and pyramidal morphology (Domnisoru et al., 2013, Schmidt-Hieber and Häusser, 2013, Sun et al., 2015). The stellate cells in layer 2 (L2SCs) have extensive local intra-laminar connections (Beed et al., 2013, Couey et al., 2013, Fuchs et al., 2016, Pastoll et al., 2013), discrete projections to principal cells in layer 5b (Sürmeli et al., 2015), and long-range projections to the hippocampus (Schwartz and Coleman, 1981, Varga et al., 2010), making them well placed to coordinate and distribute grid and other spatial signals. Inactivation of L2SCs suppresses contextual fear conditioning (Kitamura et al., 2015). However, while grid cells encode representations of an animal’s current location and theoretical models predict they may be used to plan trajectories to future locations (Burak and Fiete, 2009, Bush et al., 2015, Stemmler et al., 2015), it is not clear whether output from L2SCs is required for behaviors that require estimation of specific locations.

Because of the metric properties of their firing fields, grid cells have been proposed to encode the output of a path integration computation (McNaughton et al., 2006), and many theoretical models of grid firing perform path integration (Burgess and O’Keefe, 2011, Giocomo et al., 2011, Zilli, 2012). For example, continuous attractor network models (McNaughton et al., 2006), which have been proposed to account for grid firing based on connectivity between L2SCs and nearby interneurons (Couey et al., 2013, Pastoll et al., 2013), generate location estimates by integrating external spatial cues with velocity signals (Burak and Fiete, 2009, Fuhs and Touretzky, 2006, Guanella et al., 2007). Other models demonstrate that grid firing need not be the result of a path integration computation (Cheung, 2016, Kropff and Treves, 2008), and theoretical analyses suggest that the grid code may simply serve as a high-capacity spatial representation (Mathis et al., 2012, Sreenivasan and Fiete, 2011). In support of a path integration role, lesioning the MEC impairs measures of path integration in real world behavioral tasks (Jacob et al., 2017, Van Cauter et al., 2013, Winter et al., 2013); but, with this approach, it is not possible to distinguish roles of individual cell populations, and the contributions of surrounding brain structures are difficult to rule out. Moreover, to avoid confounding visual cues that might support landmark or beaconing strategies, path integration in real world conditions must be tested in the dark, which may impair representation by grid cells (Chen et al., 2016, Pérez-Escobar et al., 2016).

The hypothesized importance of L2SCs within circuitry that generates grid firing leads to the prediction that L2SCs are critical for spatial behaviors. The nature of the predicted contribution of L2SCs to spatial behaviors depends on the model considered. While path integration has been a major focus when investigating models of grid firing, in continuous attractor network models the same circuitry, depending on the availability of external inputs, generates grid patterns either through path integration or as a consequence of external spatial drive to the grid circuit (Guanella et al., 2007, Milford et al., 2010, Pastoll et al., 2013, Solanka et al., 2015). In these models, when external spatial cues are available, they are sufficient to dictate which neurons are active; but, when external spatial cues are not available, the models estimate location relative to the last spatial cue using path integration (Pastoll et al., 2013, Solanka et al., 2015). Analysis of robot systems suggests that this feature of continuous attractor circuits may be important for resolving navigational uncertainty (Milford et al., 2010). Given these considerations, if hypothesized continuous attractor networks within the MEC are the sole source of location estimates for hippocampal neurons important for spatial memory, then the inactivation of L2SCs should impair learning of cued and path integration-based estimates of location. On the other hand, if L2SCs are the source of velocity inputs to a downstream path integrator circuit, or if cue-based information reaches the hippocampus by routes that do not involve L2SCs, then inactivation of L2SCs would impair only path integration-based estimates of location. Finally, if the function of L2SCs is restricted to the identification of context (Kitamura et al., 2015), then inactivation of L2SCs should not affect estimation of location by either cued or path integration strategies.

Here we introduce methods for behavioral dissociation, in mice, of the linear component of path integration from cue-based localization strategies. We demonstrate that virtual reality-based behaviors can probe path integration strategies while avoiding confounds from spatial cues present in real world experiments. We find that mice successfully learn to use motor-related information to locate rewards using a path integration strategy, although with accuracy that decreases rapidly as a function of distance, unlike that of cue-based strategies. To investigate roles of L2SCs, we inactivated their outputs by the expression of tetanus toxin light chain (TeLC) (Murray et al., 2011). We found that this disrupts the adoption of both path integration and cue-based behavioral strategies. Consistent with hypothesized spatial roles of L2SCs, we also found that, in real-world experiments, the inactivation of L2SCs impairs the recognition of object locations, but not recognition of novel objects. Our results provide quantitative constraints for models that aim to account for mammalian path integration, and they implicate L2SCs as a critical component of the neural circuitry for cue- and path integration-based spatial learning.

## Results

### A Behavioral Task for Quantitative Investigation of Cue- and Path Integration-Based Estimation of Location

**Figure 1:**
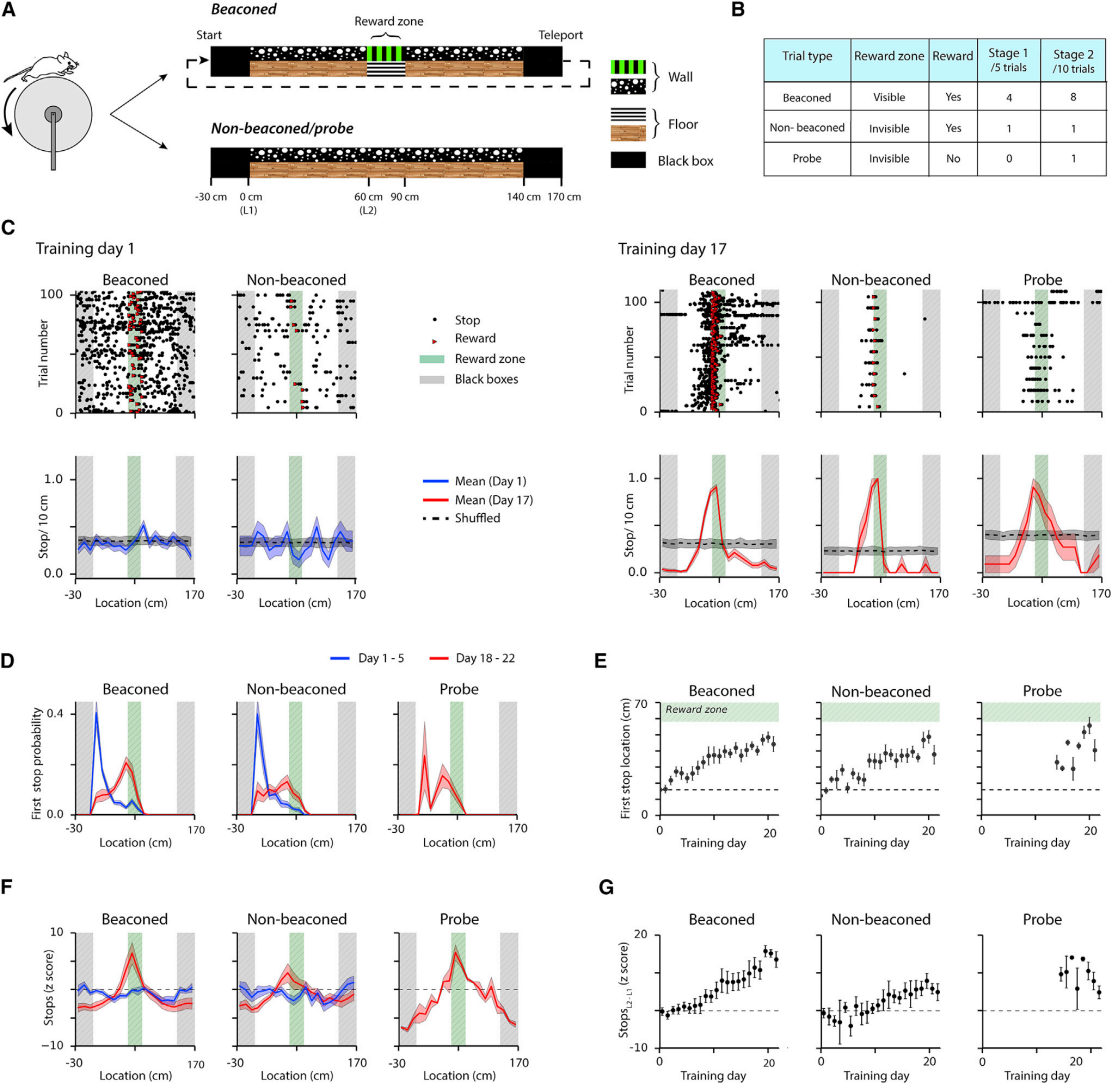
Mice Learn to Estimate Location Using a Path Integration Strategy (A) Schematic of the virtual track used on beaconed trials (upper) or non-beaconed and probe trials (lower). The reward location is indicated by visual cues from stripes on the floor and walls of the track only on the beaconed trials. (B) Configuration of trial types. (C) Examples of raster plots of stopping locations as a function of track position, separated according to trial type, on day 1 (upper left) and on day 17 (upper right), and corresponding mean number of stops/10-s bin (lower plots). Stopping locations on the raster plots are indicated by dots, which are red for locations that triggered a reward, and otherwise are black. The mean numbers of stops are indicated by solid lines and shuffled means by dashed lines. The shaded bands around the means indicate the SEM. (D) Average probability of the first stop on each trial as a function of binned track location for all mice (n = 8 mice) across days 1–5 (blue lines) and days 18–22 of training (red lines) separated according to trial type. Shaded regions indicate SEM. Bin width is 10 cm. (E) Average first stop location as a function of training day for each trial type. The location of the first stop varied as a function of day for beaconed (p < 2.2 × 10^−16^, χ(1)^2^ = 119.4, likelihood ratio test) and non-beaconed trials (p = < 2.2 × 10^−16^, χ(1)^2^ = 92.4). There was no significant difference between the three trial types on days 18–22 (p = 0.23, F(2,87) = 1.48), 1-way repeated-measures ANOVA). Error bars are SEM (N = 8 mice for beaconed and non-beaconed trials and N = 6 mice for probe trials). (F) Mean *Z* scored probability of stopping as a function of binned track location for all mice (N = 8) across days 1–5 (blue lines) and days 18–22 of training (red lines) separated according to trial type. Shaded regions indicate SEM. Bin width is 10 cm. (G) Spatial stopping behavior, quantified by the difference between the z score at the start of the track and at the entrance to the reward zone, plotted as a function of training day for each trial type. The difference varied as a function of day for beaconed (p < 2.2 × 10^−16^, χ(1)^2^ = 142.5, likelihood ratio test) and non-beaconed groups (p = 7.4 × 10^−10^, χ(1)^2^ = 37.9). Probe trials on days 18–22 did not differ from non-beaconed trials (p = 0.78, F(1,50) = 0.08).

To be able to selectively investigate neural mechanisms for beaconing and path integration, we developed a behavioral test that, depending on the task configuration, can be effectively solved either using local cues or by estimating location from self-motion signals, but in which potentially confounding external spatial cues are not available (Figure 1A; Movie S1). We trained mice to stop at a defined location on a virtual linear track to receive rewards. The virtual track had clearly identifiable start and end zones connected by a corridor that, apart from the clearly marked reward zone, did not contain any location-specific cues. In contrast to real world experiments, in which the distant end of a track could be used as a cue to estimate location, the end of the virtual track was not visible from the reward zone and, therefore, could not be used as a cue. In the first phase of training, the location of the reward zone was clearly indicated to the mouse using local visual cues on 4 of every 5 trials (beaconed trials) (Figure 1B). On the fifth trial, the visual markers for the reward zone were absent, but stopping within the zone was rewarded (non-beaconed trials). After a pre-determined training period, and if mice passed a performance criterion (see the Supplemental Experimental Procedures), every second non-beaconed trial was replaced with a probe trial. On probe trials, the visual cue was absent and no reward was delivered. Performance on non-beaconed and probe trials can be used to test whether mice estimate their position on the track using a path integration strategy, while probe trials also enable search strategies to be investigated.

We asked if mice locate the reward zone using a path integration strategy. On the first day of training, mice often received rewards by stopping in the reward zone, but there was no apparent spatial organization to their stopping behavior (Figure 1C). With training, the behavior of the mice changed, such that on leaving the start of the track mice ran, typically without stopping, to a region close to the reward zone, at which point they advanced at short intervals until they obtained a reward (Figure 1C). This change in behavior was readily observed in all mice as an increase in the distance from the start zone to the location of the first stop (Figures 1C–1E), from location-dependent changes in the probability of stopping (Figure S1A), and by a reduction in running speed as the animal approached the reward zone (Figure S1B). To enable quantitative comparison of stopping strategies between animals, we calculated *Z* scored stopping probabilities by normalizing the mean probability of stopping at a given location to the mean and SD predicted by shuffled datasets (see the Supplemental Experimental Procedures). The distribution of stops in naive animals was similar in the experimental and shuffled datasets, whereas in trained animals the probability of stopping on the first part of the track was reduced and immediately before the reward zone was increased, relative to the shuffled data (Figures 1C, 1F, and 1G). Strikingly, this spatially selective stopping behavior was maintained on both non-beaconed and probe trials (Figures 1C–1G; Figures S1A and S1B). Because on these trials visual cues that might indicate the correct stopping location were absent, and on probe trials cues associated with the dispensing of rewards were also not available, these data indicate that mice solve the task using a path integration strategy. On the probe trials, mice typically stopped near the start of the hidden reward zone and continued to stop at short intervals until they reached the end of the hidden reward zone, at which point trained mice typically ran continuously to the end of the track to initiate a new trial (Figure 1C), suggesting that mice may also use path integration strategies to estimate the length of the reward zone.

### Path Integration-Based Estimates of Location Update Using Self-Motion Signals

**Figure 2:**
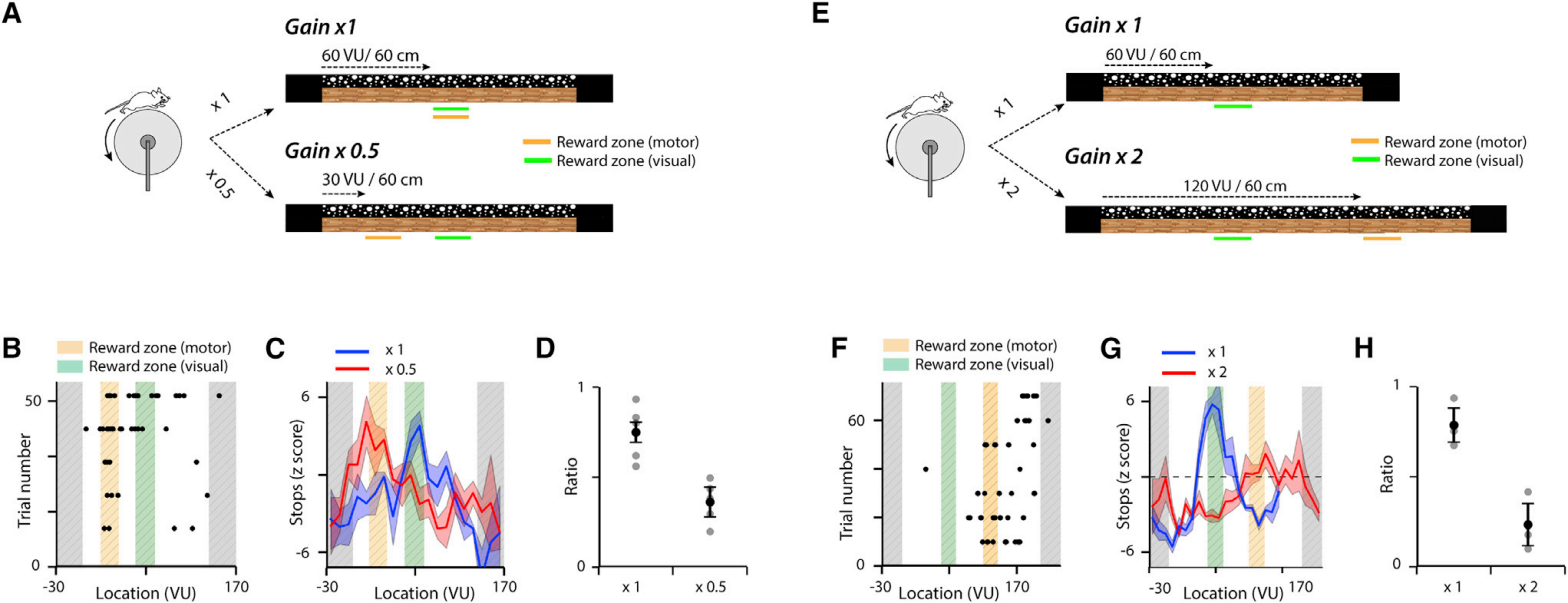
Path Integration Uses Motor-Related Movement Signals (A and E) Schematic of track designs used to test a decrease (A) or an increase (E) in the gain between motor and visual reference frames. For standard trials, for every 60 cm mice run on the treadmill, the visual track moves 60 virtual units (VU). On reduced gain trials, for every 60 cm mice run, the visual track moves 30 VU. For increased gain trials the visual track moves 120 VU for every 60 cm mice run. (B and F) Example plots of stop locations from single mice for trials in which the gain between treadmill movement and visual update of the track is reduced by 0.5 (B) or increased by ×2 (F). The trial number refers to all trials, but for clarity only data from gain change trials are shown. (C and G) Average of *Z* scored stop locations across all mice for control probe trials (×1) and trials on which the gain is reduced (C) or increased (G). Averaged data are plotted as ± SEM (N = 5 mice for ×0.5 gain, N = 4 mice for ×2 gain). (D and H) To quantify the effects of the gain change we compared, for each trial type, the ratio of stops in the location of the reward zone in the visual reference frame (orange) to the sum of the number of stops in the reward zone in the visual and motor reference frames (green). The ratio is modified by reducing (t(4) = 3.7, p = 0.021, paired t test) (D) or increasing gain (t(3) = 6.5, p = 0.0073) (H). Error bars indicate SEM. Thus, on trials with reduced gain (B–D), or increased gain (F–H), stops occur in anticipation of the reward zone location in the motor reference frame.

In principle, path integration can be achieved by updating location estimates using either visual (e.g., optic flow) or self-motion (e.g., proprioceptive feedback and motor efference) signals (Etienne et al., 1996, Raudies et al., 2016). To distinguish between these possibilities, we altered the relationship between treadmill movement and update of the visual projection of the track during probe trials. We found that, on these gain manipulation trials, mice continue to stop at a location predicted by the treadmill movement rather than by the visually perceived track movement (Figures 2A–2H). Thus, when the rate of update of the visual projection of the track was halved (Figures 2A–2D), or doubled (Figures 2E–2H), the first stop location and peak average stop location were just ahead of the reward zone predicted by the treadmill position. It is possible that animals use estimates of time instead of, or as well as, distance to complete the task (Kraus et al., 2015). To address this, we examined stopping location as a function of average running speed. If mice were using elapsed time to estimate location, then the distribution of stopping locations should depend on running speed, as mice running faster would cover a greater distance. In contrast, we found little or no dependence of the first stop location on running speed (Figures S2A and S2B). Thus, mice appear able to solve the task using a path integration strategy based on self-motion cues.

### Accuracy of Path Integration Decreases with Distance from Location Cues

**Figure 3:**
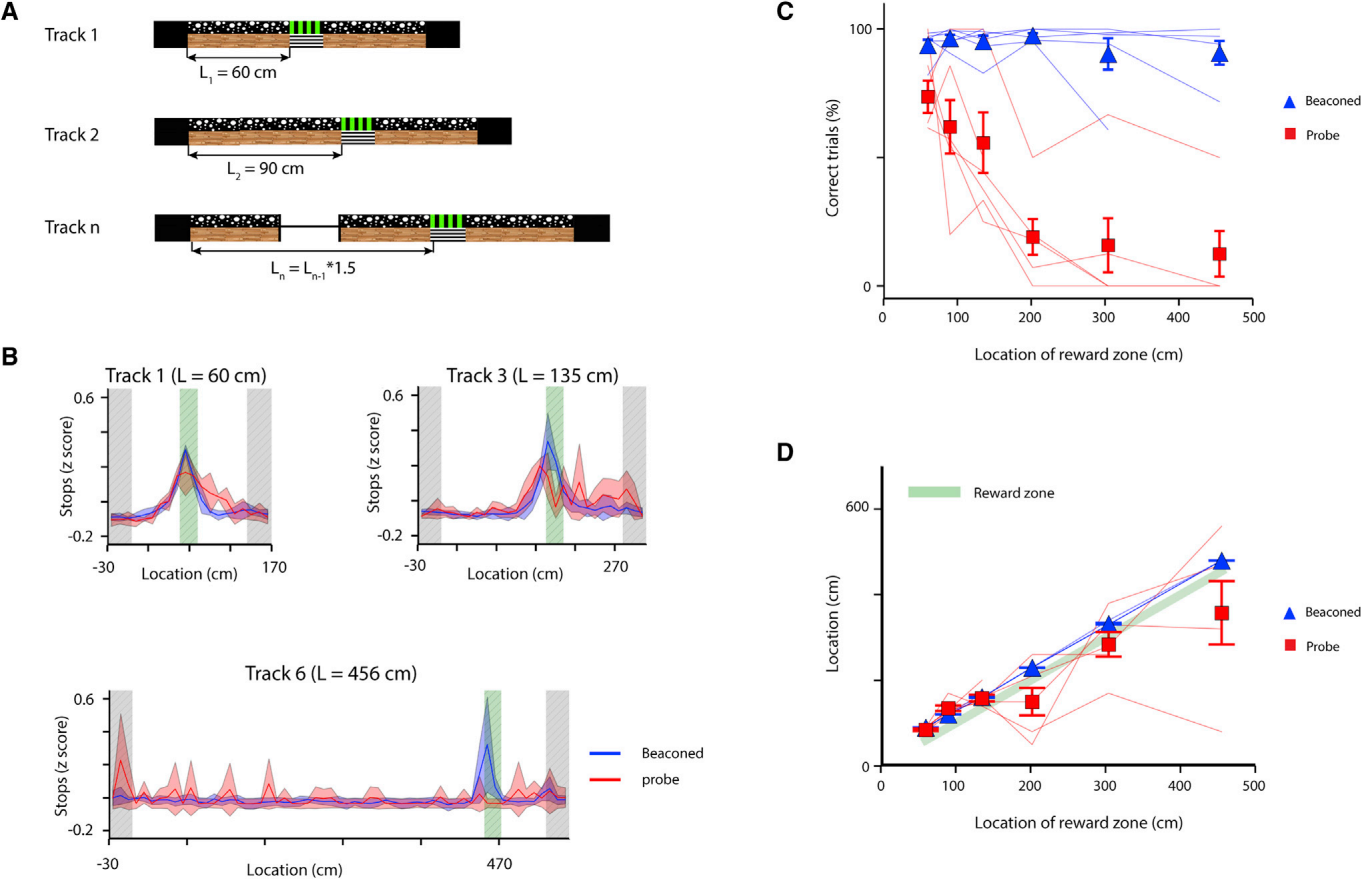
Path Integration Becomes Less Accurate with Increasing Distance (A) For tracks of increasing length the distance from the start zone to the reward zone increases as indicated. The length of other parts of the track does not change. (B) *Z* scored probability of stopping during probe trials as a function of location for three tracks of increasing length. (C) Mean success rate at obtaining rewards as a function of distance from the start of the track to the reward zone separated according to trial type (N = 7 mice). The success rate depended on distance to the reward zone (p = 8.74 × 10^−7^, F(1,60) = 30.1) and accuracy of probe trials differed from beaconed trials (p < 10^−16^, F(1,60) = 139.3). Success rate depended on distance to the reward zone for probe trial data (χ^2^(1) = 22.8, p = 1.8 × 10^−6^), but not for beaconed trial data (χ^2^(1) = 1.87, p = 0.17). (D) Mean of the most frequent stop location plotted as a function of distance. The most frequent stop location depended on distance to the reward zone (p < 10^−16^, F(1,60) = 276.5, 2-way repeated-measures ANOVA), but was independent of trial type (p = 0.2, F(1,60) = 1.5). Error bars in (C) and (D) indicate SEM.

Theoretical models predict that internal noise will result in errors in path integration that increase with distance traveled (Cheung and Vickerstaff, 2010). The extent to which such errors limit the ability of mice to estimate location by path integration mechanisms is unclear. We therefore trained mice, using tracks of increasing length, to locate rewards at distances from 60 cm to >4 m from the start zone (Figure 3). After mice reached a criterion performance (see the Supplemental Experimental Procedures) on a track of a given length, we increased the distance to the reward zone by a factor of 1.5 (Figure 3A). We found that, as the length of the track was increased, mice continued to stop in the reward zone on a high proportion of beaconed trials (Figures 3B and 3C). Adaptation to the new reward zone location was usually apparent within the first 5 trials of the first session with the new track, indicating that visual cues can rapidly reconfigure the behavior. In contrast, the fraction of probe trials in which mice stopped in the reward zone dropped substantially as the track length increased (Figure 3C; Figure S3A). This steep drop in performance was also seen when success was evaluated as a function of time taken to reach the reward zone for tracks of different lengths (Figure S3B). Examination of stopping patterns indicated that, for intermediate-length tracks, the stopping locations were centered around the reward zone, even as the number of correct trials decreased (Figures 3B and 3D; Figure S3A). However, from trial to trial stop locations were variable and often were outside the reward zone, explaining the high number of errors (Figures 3B and 3D; Figure S3A). These observations argue against errors resulting from a residual memory for the previous stop location and are consistent with an accumulation of error in a path integrator system. Thus, in the absence of landmark cues to anchor path integration, the ability to accurately estimate location drops rapidly with distance from a known starting point in a manner that is consistent with performance of a noisy path integrator.

### Blocking the Output from L2SCs Prevents Spatial Learning

**Figure 4:**
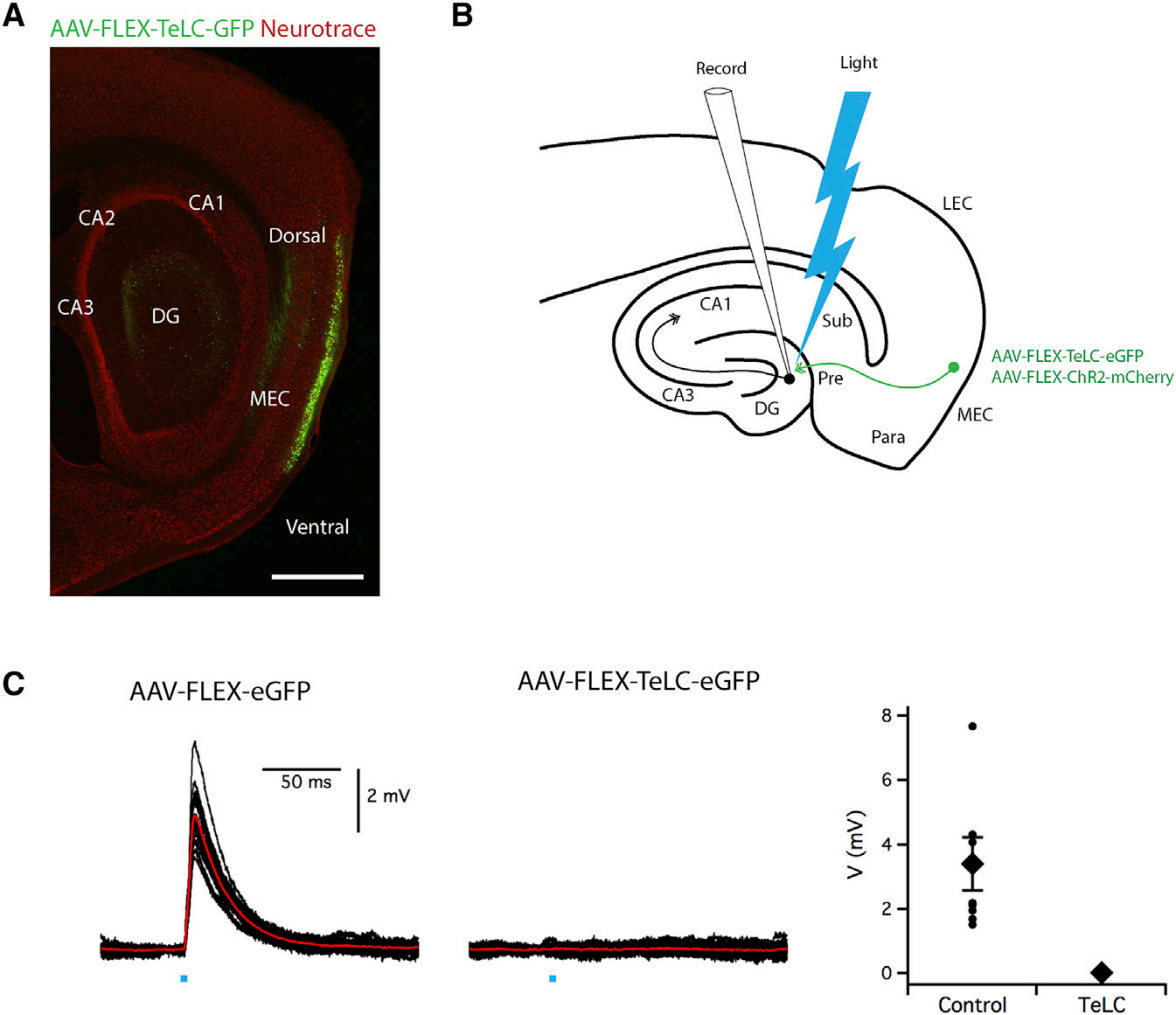
Targeted Expression of TeLC to L2SCs Abolishes Their Synaptic Output (A) Example of a sagittal section from the brain of a Sim1^Cre^ mouse following injection of AAV-TeLC-EGFP into the MEC. Scale bar, 1 mm. (B) Schematic of experiment to test the effect of TeLC expression on synaptic output from L2SCs. AAV-FLEX-ChR2-mCherry and either AAV-FLEX-TeLC-EGFP or AAV-FLEX-EGFP were injected into the MEC of Sim1^Cre^ mice. Synaptic output from L2SCs was evaluated by recording light evoked response of granule cells in the dentate gyrus. (C) Examples of membrane potential responses of dentate gyrus granule cells to optogenetic activation of L2SCs expressing ChR2 and either GFP (left) or TeLC-EGFP (middle). Responses are present in all neurons from control animals (n = 10 neurons, N = 5 mice) and were absent in all neurons from animals expressing TeLC-EGFP (n = 8 neurons, N = 4 mice). The peak response was reduced by expression of TeLC-EGFP (right) (p = 0, percentile bootstrap comparison of control and TeLC-EGFP groups, test statistic = 2.125, 95% confidence interval [1.69, 4.29]). Circles are individual neurons, diamonds are the population average. Two neurons from two control mice were excluded from the plot and statistical analysis as they showed very large responses that reached action potential threshold preventing their quantification. Error bars indicate SEM.

Because superficial layers of the MEC are enriched with neurons that have spatial firing properties (Diehl et al., 2017, Hafting et al., 2005, Hardcastle et al., 2017, Solstad et al., 2008), and as grid-firing patterns generated by neurons in the MEC are consistent with the output of a neural path integrator (McNaughton et al., 2006), we asked if neural circuitry in superficial MEC is required for learning of the beaconed or path integration components of the location estimation task. We focused on L2SCs, as the highest density of grid cells is in layer 2 (Sargolini et al., 2006), and L2SCs have grid-firing fields (Domnisoru et al., 2013). To be able to selectively manipulate L2SCs, we took advantage of Sim1^Cre^ mice, which we found previously give specific genetic access to L2SCs (Sürmeli et al., 2015). To test the role of L2SCs, we blocked their synaptic output by injecting an adeno-associated virus (AAV) that expresses TeLC and EGFP conditionally on the presence of Cre (AAV-FLEX-TeLC-EGFP) (Murray et al., 2011) into the superficial MEC of Sim1^Cre^ mice (Figure 4A). As a control, we used an AAV that expresses only EGFP (AAV-FLEX-EGFP). Expression of EGFP was restricted to L2SCs and was absent from the surrounding neurons (Figure 4A; Figures S4 and S5). To test whether expression of TeLC blocks SC output, we co-expressed channelrhodopsin 2 (ChR2) in L2SCs, to enable their optical activation, along with either TeLC-EGFP or EGFP (Figure 4B). When we recorded from downstream granule cells in the hippocampus, we observed synaptic currents following light activation of ChR2 in slices from mice expressing the control EGFP construct, but not in slices from mice expressing TeLC (Figure 4C). Thus, targeted expression of TeLC using Sim1^Cre^ mice enables the block of synaptic output from L2SCs.

**Figure 5:**
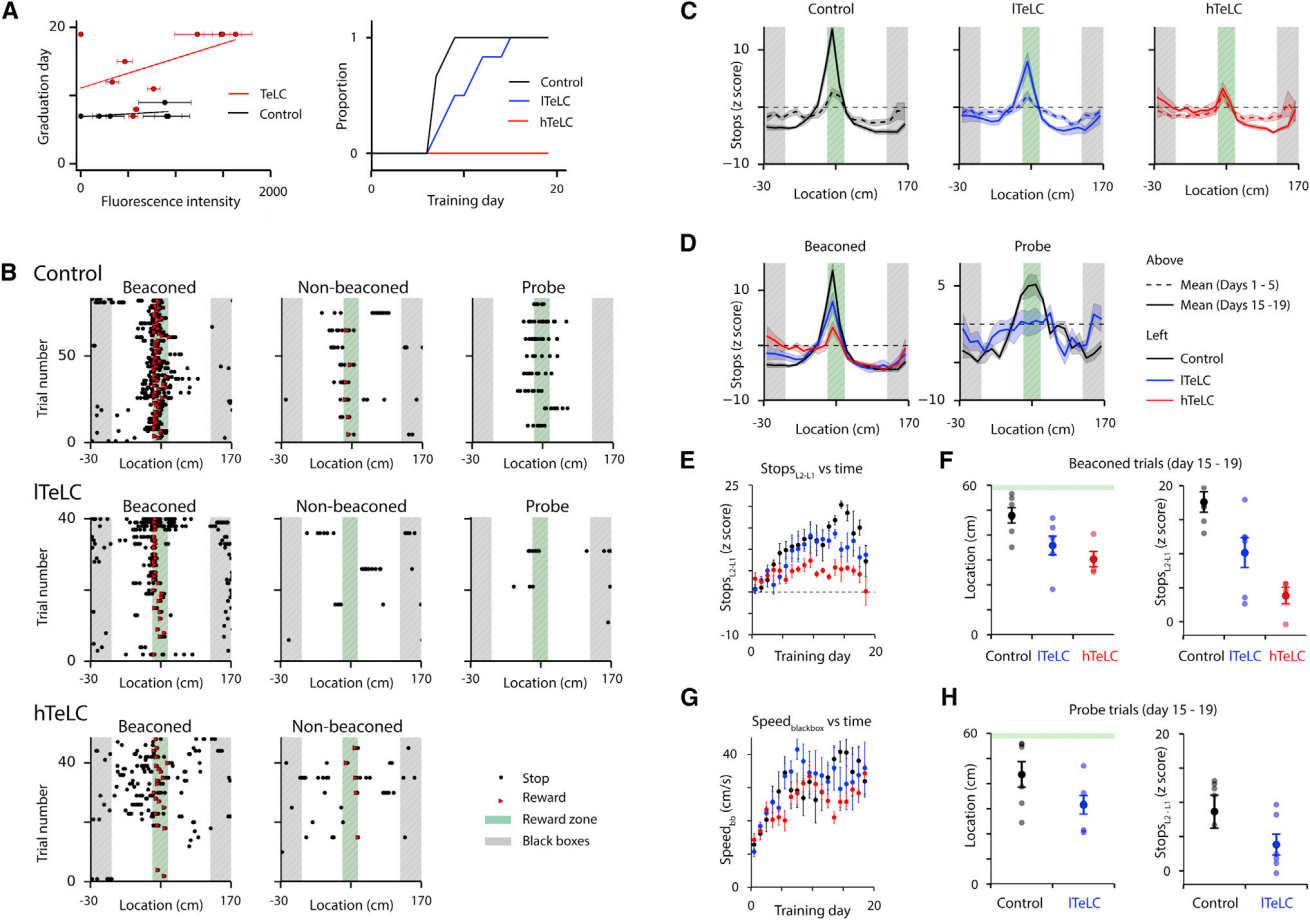
Inactivation of L2SCs Impairs Estimation of Location (A) Day of the experiment on which each mouse from TeLC (N = 10) and control groups (N = 6) met the performance criteria to graduate from stage 1 (beaconed and non-beaconed trials) to stage 2 (beaconed, non-beaconed and probe trials) as a function of mean intensity of GFP fluorescence in layer 2 of the dorsal MEC (left), and proportion of mice that had graduated to stage 2 as a function of training day (right). The graduation day correlated with fluorescence intensity for the TeLC group (p = 0.00077, robust least-squares regression), but not the control group (p = 0.38; comparison of GFP and TeLC-GFP groups: p = 0.0012, for statistical analysis see the Experimental Procedures). (B) Examples of rasters of stopping locations on day 17 of training for a control mouse, and for mice with high and low expression levels of TeLC (lTeLC and hTeLC). Black dots indicating stopping location are absent on some trials because the animal did not stop. (C) Mean z-scored probability of stopping as a function of track location during beaconed trials for GFP only control (left), lTeLC (center), and hTeLC mice (right) on days 1–5 and days 15–19. (D) Comparison of mean z-scored probability of stopping for trained mice (days 15–19) for each group on beaconed trials (left) and probe trials (right). (E) The difference, between the start of the track and the start of the reward zone, in the probability of stopping (Stops_L2-L1_) (locations L1 and L2 are indicated in Figure 1A) increased with training for GFP mice (p = 1.4 × 10^−10^, χ(1)^2^ = 41.2, likelihood ratio test) and lTeLC mice (p = 5.2 × 10^−8^, χ(1)^2^ = 29.6), but not for hTeLC mice (p = 0.89, χ(1)^2^ = 0.017). (F) Analysis of spatial strategy for beaconed trials during days 15-19. The mean location of the first stop (left) differed between control (GFP) and all TeLC mice (lTeLC and hTeLC combined) (p = 0.021, percentile bootstrap, test statistic = 16.1, confidence interval [2.9, 26.1]), and hTeLC mice differed from control mice (p = 0.01, percentile bootstrap corrected for multiple comparisons, test statistic = 22.2, 95% confidence interval [6.4, 28.6]), but there was no significant difference between lTeLC and control mice (p = 0.09, test statistic = 13.1, 95% confidence interval [−0.23, 26.8]). Stops_L2-L1_ (right) differed between control and all TeLC mice (lTeLC and hTeLC combined) (p = 0.00052, test statistic = 11.75, 95% confidence interval [3.26, 16.1]), and hTeLC and lTeLC mice differed from control mice (hTeLC: p = 0.0, test statistic = 12.1, 95% confidence interval [8.4, 18.1]; lTeLC: p = 0.034, test statistic = 5.42, 95% confidence interval [1.1, 14.1]). (G) Running speed in the black box at the end of the track increased with training for all groups of mice (GFP: p = 3.5 × 10^−11^, χ(1)^2^ = 43.7; lTeLC: p = 0.0013, χ(1)^2^ = 10.4; hTeLC: p = 6.5 × 10^−6^, χ(1)^2^ = 20.3). During week 4 there was no difference between groups in their running speed within the black box (adjusted p = > 0.7 for all comparisons, percentile bootstrap test). (H) Analysis of spatial strategy for probe trials during days 15–19. The first stop location (left) differed between lTeLC and GFP groups (p = 0.045, test-statistic = 17.9, 95% confidence interval [0.64, 34.5]). Stops_L2-L1_ during probe trials (right) did not differ significantly between lTeLC and control mice (p = 0.097, test statistic = 10.7, 95% confidence interval [−1.2, 12.2]). Error bars in (A) and (E)–(H) indicate SEM.

Does blocking output from L2SCs affect the ability of mice to learn a rewarded location? To address this, we injected the MEC of Sim1^Cre^ mice with AAV-FLEX-TeLC-EGFP (n = 10) or AAV-FLEX-EGFP (n = 6). We trained the mice for 3 weeks in the virtual location estimation task, and then we sacrificed them in order to analyze the extent of expression of the viral transgenes (Figures S4 and S5). We found that the proportion of trials on which mice stopped in the reward zone was reduced for the TeLC-expressing mice compared to control mice. This was manifest as a delay to reach the criterion for the introduction of probe trials into the experiment (control: 7.33 ± 0.33 days, TeLC: 14.3 ± 1.69 days; p = 0.00069, percentile bootstrap comparison of control and TeLC groups, test statistic = −6.5, 95% confidence interval [−13,−2.5]). The delay depended on the extent of viral transduction in the TeLC group, but not in the control group (Figure 5A). Because expression and task progression were variable between animals, for further analysis we divided the mice, according to the extent of labeling of neurons in the dorsal MEC, into groups with high (hTeLC, n = 4) and low (lTeLC, n = 6) expression of TeLC (Figure 5A; Figure S4). Whereas all control mice reached the criterion for inclusion of probe trials within 9 days (7.33 ± 0.33 days), the lTeLC mice were delayed (10.5 ± 1.18 days; p = 0.019, test statistic = −3.5, 95% confidence interval [−6.5,−0.5]), and the hTeLC mice did not meet the criteria within the 19 days of the experiment. We note that, in 3 of 4 mice from the hTeLC group, we observed small numbers of labeled cells in L5a. Because very few cells were labeled in L5a in any animal and as the behavioral impairment was present in the mouse that had no detectable expression in L5a, expression of TeLC in deep layers is unlikely to account for the observed behavioral changes (Figure S6). Thus, these data indicate that output from L2SCs plays a key role in learning the location of a reward zone, with the number of available L2SCs determining the rate of learning.

### Learning Deficits following the Block of L2SC Output Include Cue- and Path Integration-Based Estimation of Location

How do the deficits in performance of TeLC mice relate to the acquisition of a spatial stopping strategy? We first compared stopping strategies used by mice after >14 days training with stopping strategies used over the first 5 days of training. We found that, after training, control mice and lTeLC mice both demonstrated spatial stopping strategies on beaconed trials (Figures 5B and 5C). The distribution of stop locations was distinct from that of naive mice (Figure 5C), suggesting that mice in both groups learn to stop in the region of the reward zone. In contrast, hTeLC mice did not develop a clear spatial stopping strategy (Figures 5B–5D), and the distribution of stop locations appeared similar to the first week of training (Figures 5C and 5D).

To quantitatively compare these changes, we evaluated the difference between stopping probability at the start of the track and the start of the reward zone, which we refer to as Stops_L2-L1_, as a function of the day of the experiment (Figure 5E). We found that Stops_L2-L1_ measured during beaconed trials increased with training for the control group and the lTeLC group, consistent with these mice learning a spatial stopping strategy, but did not change for the hTeLC group (Figure 5E). Comparison of stopping patterns on days 15–19 indicated that mice in the lTeLC group were, nevertheless, impaired relative to the control group, with first stop location and Stops_L2-L1_ that were intermediate to that of the hTeLC group (Figure 5F). We noticed that training in the task was also associated with an increase in running speed that was particularly apparent in the part of the track between the end of the reward zone and the end of the black box that separates tracks between trials (Figure S1B). To quantify this change, we evaluated the average running speed in the black box as a function of day of the experiment. In contrast to measures of the spatial stopping strategy, running speed in the black box increased with training for control, lTeLC, and hTeLC mice (Figure 5G), suggesting that all mice learn about the structure of the task such that they increase their running speed to minimize the time between consecutive rewards. Consistent with this interpretation, we found no detectable difference between groups in their running speed on days 15–19 (Figure 5G). Thus, inactivation of L2SCs impairs learning of a location, but not task structure, with the size of the deficit dependent on the extent of inactivation.

To evaluate the effects of inactivation of L2SCs on path integration, we compared probe trials between lTeLC and control groups (Figures 5D and 5H). In contrast to control mice, the average distribution of stop locations for lTeLC mice showed little spatial organization on probe trials (Figure 5D). The lTeLC group differed significantly from the EGFP group in the first stop location (Figure 5H), but not the *Z* score difference between the start of the track and the reward zone (Figure 5H). Analysis of preferred stopping locations and running speed also indicated deficits in the lTeLC group compared to the control mice (Figures S6B and S6C). Thus, estimation of location by lTeLC mice on probe trials appears to be impaired.

Together, these results indicate that L2SCs are required to learn a reward location in environments in which beaconing and path integration are the only available strategies, while inactivation of L2SCs does not appear to influence learning about task structure.

### L2SC Output Is Required for Object Location Recognition

**Figure 6:**
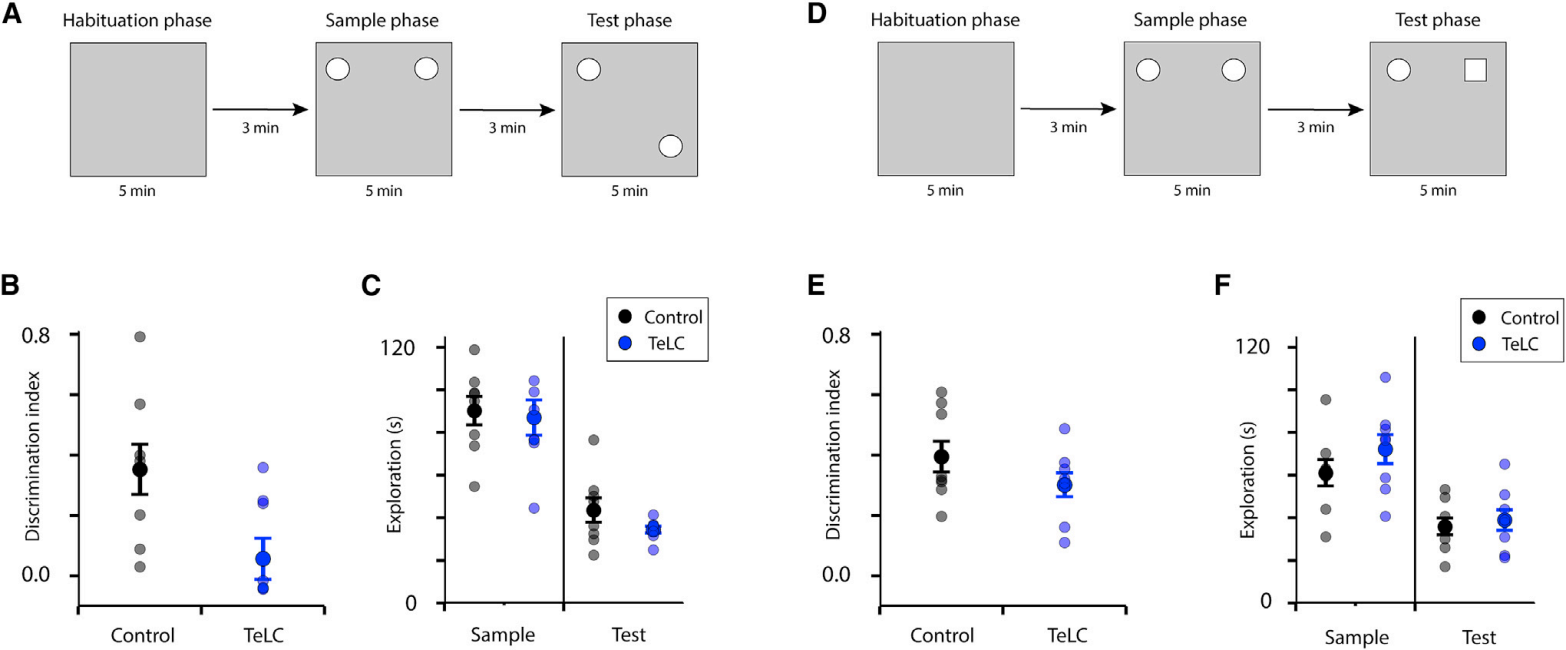
Layer 2 Stellate Cells Are Required for Object Location Recognition (A and D) Schematized organization of the object location (A) and object recognition (D) experiments. In the test phase of the object location experiment one object is moved to a novel location (A), whereas in the object recognition experiment a novel object is introduced at a familiar location (D). (B and E) The discrimination index for control mice (Control, N = 8) differed significantly from mice with output from L2SCs inactivated (TeLC, N = 8) in the object location experiment (p = 0.022, unpaired t test, df = 14, t = 2.58) (B), but not the object recognition experiment (p = 0.19, unpaired t test, df = 14, t = 1.37) (E). (C and F) Total exploration times in the sample and test phases did not differ between animals in the object location experiment (sample phase p = 0.78, df = 14, t = 0.28; test phase p = 0.17, df = 14, t = 1.45) (C) or in the object recognition experiment (sample phase p = 0.27, df = 14, t = −1.14; test phase p = 0.66, df = 14, t = −0.45) (F), indicating that different recognition scores do not result from differences in overall exploration. Error bars in (B), (C), (E), and (F) indicate SEM.

Finally, to establish whether the deficits we identified in virtual reality-based tests of location estimation extend to real world behaviors, we investigated the effects on location recognition of expressing TeLC in L2SCs. We used an object location memory task that takes advantage of an animal’s spontaneous tendency to explore relocated objects (Figure 6A). During a sample phase, mice explored an arena containing two identical objects. During a test phase, one of the objects was relocated to a novel location and mice were allowed to re-explore the arena. To compare the two groups, we calculated the relative time spent exploring each object during the test phase. Whereas control mice that expressed only EGFP in L2SCs showed preferential exploration of the relocated object over the stationary object (discrimination ratio significantly greater than 0, p = 0.0053, one-sample t test versus 0, df = 7, t = 3.99), mice that expressed TeLC in L2SCs did not (p = 0.45, one-sample t test versus 0, df = 7, t = 0.79) (Figure 6B). Consistent with this, discrimination between the objects was substantially lower for mice expressing TeLC in L2SCs compared with control mice (Figure 6B). There was no significant difference in total exploration times between control mice and mice expressing TeLC in L2SCs (Figure 6C), indicating that the differences in discrimination ratios did not reflect disparities in exploration.

To test whether impairments in object location discrimination following the expression of TeLC in L2SCs extend to recognition of objects, we tested the same mice in a version of the task in which a familiar object was replaced with a novel object in the test phase (Figure 6D). Total exploration during the sample and test phases of the task were similar between control and TeLC-expressing mice; both groups showed above-chance discrimination of the novel object, and there was no significant difference in discrimination indexes between control and TeLC-expressing mice, indicating that object recognition was unimpaired (Figures 6E and 6F). Together, these results indicate that, while L2SCs are not required for the recognition of objects, blocking their output impairs discrimination between novel and familiar object locations.

## Discussion

By using a virtual reality-based behavioral task, we dissociate estimation of location by beaconing and path integration in conditions in which both visual and motor-related movement information is available. Our results indicate that the accuracy of location estimation by path integration drops steeply as a function of distance, and they suggest that, when visual motion signals are available, mice nevertheless use a motor-based reference frame for path integration. By selectively inhibiting output from L2SCs, we find that this population of medial entorhinal neurons is required for mice to learn to estimate location in the virtual reality task and in a real world object location recognition task. Our data constrain possible models for behavioral estimation of location, and they provide evidence for a critical involvement of grid cell circuitry.

Because spatial cognition involves parallel perceptual and memory processes, with multiple strategies available to provide the brain with estimates of location, investigation of specific cognitive mechanisms in isolation is challenging. Our approach using a virtual location estimation task is in contrast to real world experiments that isolate path integration from other behavioral strategies through the use of environmental manipulations (Etienne and Jeffery, 2004, Jacob et al., 2017, Van Cauter et al., 2013, Winter et al., 2013). In real world experiments, evaluation of path integration requires the execution of behaviors in darkness in order to prevent confounding influences of visual landmark cues, but this manipulation also removes visual input required for normal function of grid cell circuits, and, therefore, it could impair path integration mechanisms that rely on grid cells (Chen et al., 2016, Pérez-Escobar et al., 2016). Confounding contributions from residual spatial cues, for example, from odors or sounds, are also difficult to fully exclude in real world experiments. In contrast, in the virtual reality-based tasks we introduce here, both visual and motor information is available to the mice, while odor, auditory, or visual cues in the experimental room are not useful in solving the task. Thus, because we were able to represent reward locations in an environment that is devoid of triangulation and beaconing cues, we have been able to specifically probe psychophysical properties of path integration, including dependence on distance and the roles of visual and motor reference frames.

What is the nature of the movement signals used to estimate location by path integration? While place and grid cells can encode elapsed time as well as location (Kraus et al., 2015, Pastalkova et al., 2008), the estimation of time rather than distance is unlikely to explain our observations, as the time taken by an animal to reach the reward zone from the start of the track was a poor predictor of stopping location (Figures S2A and S2B). Recordings from hippocampal place cells suggest that either motor or visual reference frames can be used to represent location (Chen et al., 2013). Because we find that the locations at which mice stop followed the physical distance moved on the treadmill, rather than that predicted by visual signals from the projected track (Figures 2A–2D), our data suggest that behavioral estimation of location by path integration uses a motor-based reference frame. The origin of the motor signals driving the path integrator is unclear, but it may include copies of centrally generated motor commands or proprioceptive feedback. Because mice were head fixed and, therefore, vestibular output is effectively clamped, a necessary role of vestibular motion signals in our experimental conditions can be ruled out. Nevertheless, it is possible that, in different behavioral conditions, the signals used to generate location estimates by path integration may differ.

A critical constraint on behavioral use of path integration to estimate location is the extent to which estimates drift in the absence of spatial cues to anchor the path integrator (Cheung and Vickerstaff, 2010). In models that account for grid cell firing through a path integration mechanism, grid patterns are stable in the absence of noise; but, when noise is introduced into the neural circuitry, the grid pattern drifts unless additional spatial input is provided (Burak and Fiete, 2009, Guanella et al., 2007, Solanka et al., 2015, Zilli and Hasselmo, 2010). While models differ in their assumptions about the rate at which drift accumulates, in all models additional information about location is required to correct drift. Indeed, as we discuss below, when this additional information is available, for example, from beaconing cues, it may dominate output from circuits capable of path integration. We find that the estimation of location through path integration, but not by beaconing, becomes unreliable for distances >2 m. Since the estimation of longer distances might, in principle, be improved if additional training reduces the sensitivity of the path integrator to noise, our results place only a lower bound on performance. Nevertheless, our observations impose constraints on the behavioral scenarios under which outputs of a neural path integrator may be useful. Our results are also consistent with recent findings that grid fields appear to rapidly drift when visual stimuli are removed (Chen et al., 2016, Pérez-Escobar et al., 2016). While this loss of grid firing may result from an absence of optic flow signals as movement inputs to the grid circuit (Raudies and Hasselmo, 2015, Raudies et al., 2016), our data suggest that distance estimation uses motor signals. Visible spatial cues may instead be critical to anchor grid firing and path integration in the face of drift (Pastoll et al., 2013). The relatively rapid accumulation of drift in grid cell firing (Chen et al., 2016, Pérez-Escobar et al., 2016) and of path integration error that we describe here suggest that, in mice, path integration mechanisms may be important for moment-to-moment tracking of location rather than long-range navigation.

While cells with grid and other spatial firing properties are enriched in superficial layers of the MEC (Sargolini et al., 2006), and previously L2SCs have been shown to be important for contextual learning (Kitamura et al., 2015), it has been unclear whether they contribute to spatial behaviors. Our results provide evidence that L2SCs in the dorsal MEC are required for learning that depends on location estimation within an environment (Figures 5 and 6). These data also speak to a hypothesized role for grid cells as the output of a neural path integrator (McNaughton et al., 2006). Continuous attractor network models that generate grid fields perform path integration using speed and direction signals, but, when external spatial signals are present, they can dictate activity in these circuits. Our results with inactivation of L2SCs corroborate the prediction that, if circuits of this kind are the source of location estimates used to guide behavior, inactivation of these circuits should impair estimation of location by beaconing and by path integration. Nevertheless, additional interpretations are conceivable. Inactivation of external spatial inputs to an integrator circuit would lead to similar behavioral outcomes, although this interpretation is inconsistent with the finding that L2SCs have grid fields (Domnisoru et al., 2013). Alternatively, L2SCs may be downstream of the hypothesized path integrator circuit. In this case, L2SCs must, nevertheless, be a necessary output path by which combined path integration and beaconing signals influence spatial behaviors. The possibility that beaconing and path integration systems operating in parallel, with L2SCs required only for beaconing, appears unlikely, as in this scenario path integration behavior should be maintained after the inactivation of L2SCs.

The effects of our targeted manipulation may be manifest via interactions within L2 of the MEC (Beed et al., 2013, Couey et al., 2013, Pastoll et al., 2013), by projections from L2SCs to cell populations in deeper layers of the MEC (Sürmeli et al., 2015), or through longer range projections to the dentate gyrus and CA3 (Schwartz and Coleman, 1981). Indeed, brain regions downstream of the MEC, including the hippocampus, contain neurons with properties indicative of roles in encoding of path integration and beaconing signals (Chen et al., 2013, Samsonovich and McNaughton, 1997). While following lesions of the MEC place firing in the hippocampus is reduced (Hales et al., 2014) and learning in the water maze is impaired (Hales et al., 2014, Morrissey and Takehara-Nishiuchi, 2014, Steffenach et al., 2005), the loss of place firing is only partial, suggesting that additional spatial signals reach hippocampal structures. One possibility is that L2SCs may be specialized to integrate visual cues with spatial information (Pérez-Escobar et al., 2016, Yoo and Lee, 2017), while spatial information from olfactory or other non-visual cues reaches the hippocampus through the lateral entorhinal cortex (Leitner et al., 2016, Van Cauter et al., 2013). These signals are of no use to solve the virtual reality-based location estimation task, and they may not be sufficient for the object location recognition task under our experimental conditions.

Quantitative dissection of simple behaviors has been essential in establishing underlying computational principles and circuit mechanisms. In contrast, analysis of cognitive behaviors involving multiple sensory modalities is more challenging because of their additional behavioral complexity and because brains may have multiple neural strategies available to solve a task. By implementing a relatively simple spatial task in virtual reality, we have been able to quantitatively dissect roles of beaconing and linear path integration in estimation of location. Our experiments provide evidence to support the long-standing idea that computations by grid cells in the MEC support estimation of location, and, while alternative models remain feasible, our results corroborate key predictions of grid cell models that perform path integration in a manner that integrates external spatial input with velocity signals. Finally, our results may help link deficits in spatial cognition found in dementia to underlying circuit mechanisms. Conceptually similar virtual tasks may be useful as assays of early deficits in dementia, while the key roles we identify for L2SCs suggest that damage restricted to a single cell population at very early stages of degeneration may be detectable by appropriately designed behavioral tests.

## Experimental Procedures

Further details and an outline of methods and resources used in this work can be found in the Supplemental Experimental Procedures.

### Animals

All animal experiments were carried out under a project license granted by the UK Home Office, were approved by the Animal Welfare and Ethical Review Board (AWERB) of the University of Edinburgh School of Medicine and Veterinary Medicine, and conformed with the UK Animals (Scientific Procedures) Act 1986 and the European Directive 86/609/EEC on the protection of animals used for experimental purposes. Male and female mice, aged 7–12 weeks, were used for all experiments. Mice were randomly allocated to experimental groups.

### Data Analysis and Statistical Methods

To quantify virus expression, we measured mean GFP fluorescence using FIJI. Confocal images were opened using the Bio-Formats package (Linkert et al., 2010). Collection, analysis, and presentation of data from virtual reality-based behavioral experiments were performed using custom scripts written in python 3.5 (https://www.python.org) using Numpy version (v.)1.8.1, Scipy v.0.11.0b1, and Matplotlib v.1.5.1 packages. Scripts were written using Spyder 2.3 (www.pythonhosted.org/spyder). Electrophysiology data were analyzed using IGORpro (Wavemetrics). For object exploration tasks, behavior was quantified using the Multitimer scoring system (Vogel-Ciernia and Wood, 2014), and manual scores were confirmed by repeating the scoring using AnyMaze (http://www.anymaze.co.uk/) on video recordings of the mouse’s exploration. Full details of quantification are provided in the Supplemental Experimental Procedures.

Statistical analysis was performed in R v.3.30 (R Core Team, 2014). Scripts were written and run using RStudio 0.99.902 (RStudio Team, 2015; https://www.rstudio.com). Details of data distributions and tests are given in the main text and figures. When a measure was obtained repeatedly from the same animal, the mean for that animal was used for population level analyses unless indicated otherwise. Linear mixed effect models (LMEs) were fit using lme 4 1.1-12 (Bates et al., 2015). Animal identity was included in models as a random effect and the variable of interest as a fixed effect. To evaluate significance of effects using LMEs, the model without the variable of interest (a reduced/null model) was compared to the model with the variable of interest using a likelihood ratio test. Because for experiments comparing effects of expression of GFP with TeLC-GFP (Figures 4 and 5) the distribution of the data appeared clearly non-normal, for analysis of these experiments we used robust statistical methods to compare groups (Wilcox, 2016). These were implemented in R using the packages WRS (https://github.com/nicebread/WRS) and WRS2 (v.0.9-2 from https://cran.r-project.org). Comparisons of groups used the percentile bootstrap method. For independent groups, differences between medians were evaluated using the R function medpb2 (in WRS2). For dependent groups, the bootdpci function (in WRS) was used to compare 20% trimmed means. Results are reported using 10^5^ bootstrap samples. Linear regression was performed using a least-squares method that allows heteroscedasticity, implemented in the R function olshc4 (WRS package), with slopes compared using the R function ols2ci (WRS package). For multiple comparisons within an experiment, reported p values were adjusted by the Benjamini and Hochberg method using the R function p.adjust.

Data and code to reproduce the analyses reported in the paper will be made available via the University of Edinburgh DataShare repository (http://dx.doi.org/10.7488/ds/2290). Analysis code will be made available via the Nolan Lab GitHub repository (https://github.com/MattNolanLab).

## Supplemental Information

Document S1. Supplemental Experimental Procedures and Figures S1–S6

Movie S1. A Trained Mouse Performing Four Consecutive Beaconed Trials followed by a Non-beaconed Trial, Related to Figure 1

Document S2. Article plus Supplemental Information
