# Supplementary material for: Stellate Cells in the Medial Entorhinal Cortex Are Required for Spatial Learning
Source: Cell Rep. 2018 Jan 30;22(5):1313–24. doi: 10.1016/j.celrep.2018.01.005 (PMC5809635; doi:10.1016/j.celrep.2018.01.005)
Supplement: Document S1. Supplemental Experimental Procedures and Figures S1–S6 [file mmc1.pdf]

Cell Reports, Volume *22*

Supplemental Information

Stellate Cells in the Medial Entorhinal Cortex

Are Required for Spatial Learning

Sarah A. Tennant, Lukas Fischer, Derek L.F. Garden, Klára Zsófia Gerlei, Cristina Martinez-Gonzalez, Christina McClure, Emma R. Wood, and Matthew F. Nolan

Supplemental Experimental Procedures

CONTACT FOR REAGENT AND RESOURCE SHARING

Matthew Nolan (mattnolan@ed.ac.uk) is the Lead Contact for reagent and resource sharing. All published reagents will be shared on an unrestricted basis; reagent requests should be directed to the lead author.

RESOURCES TABLE

REAGENT or RESOURCE	SOURCE	IDENTIFIER
Antibodies		
NeuroTraceÂ® 640/660 Deep-Red Fluorescent Nissl Stain - Solution in DMSO	Thermo Fisher Scientific Inc.	Cat# N21483
NeuroTraceÂ® 530/610 Red Fluorescent Nissl Stain - Solution in DMSO	Thermo Fisher Scientific Inc.	Cat# N21482
Rabbit anti Calbindin D-28k	Swant	Cat# CB 38
Mouse Anti-Reelin Antibody	Millipore	Cat# MAB5364
Goat anti-Rabbit IgG (H+L) Secondary Antibody, Alexa FluorÂ® 405 conjugate	Invitrogen	Cat# A-31556
Goat anti-Mouse IgG (H+L) Secondary Antibody, Alexa FluorÂ® 647 conjugate	Invitrogen	Cat# A-21236
Bacterial and Virus Strains		
AAV1/2-FLEX-TeLC-GFP	Murray et al., 2011	NA
AAV1/2-FLEX-GFP	Murray et al., 2011	NA
AAV1/2-FLEX-rev-ChR2(H134R)-mCherry	Atasoy et al., 2008	Plasmid from Addgene Cat#18916
Chemicals, Peptides, and Recombinant Proteins		
LOCTITE 3 g Transparent Gel Tube Cyanoacrylate Adhesive for Various Materials Ultra Gel	RS components	Cat# 330-4018
Paraformaldehyde powder 95%	Sigma Aldrich	Cat# 158127
Phosphate Buffer Powder	Sigma Aldrich	Cat# P7994-1EA
Simplex Powder Pink Shade S28/1	Associated Dental Products Ltd	Cat# ACR806
Simplex Rapid Liquid	Associated Dental Products Ltd	Cat# ACR924
Deposited Data		
Raw and analyzed data	This Paper	http://datashare.is.ed.ac.uk/

Analysis code	This Paper	https://github.com/orgs/MattNolanLab/
Body weight information for C57BL/6J (000664)	Jax Jaboratory	https://www.jax.org/jax-mice-and-services/strain-data-sheet-pages/body-weight-chart-000664
Experimental Models: Organisms/Strains		
Mouse: Tg(Sim1cre)KJ21Gsat/ Mmucd	MMRRC	RRID:MMRRC_034614-UCD
Mouse: JAX C57/BL6jCrl	Charles River	Cat# 000664
Software and Algorithms		
Any – Maze Behavioural Tracking Software	Stoelting Co.	http://www.anymaze.co.uk
Blender3D	Bourke and Felinto (2010)	https://www.blender.org
Fiji is just ImageJ	Schindelin et al (2012)	https://fiji.sc
Bio-Formats	Linkert et al (2010)	https://www.openmicroscopy.org/bio-formats/
Multitimer	Patrick Spooner, University of Edinburgh	NA
Python programming language v3.6.1	Van Rossum (1995)	Python Software Foundation (https://www.python.org)
R v3.4.2	RCore Team (2013)	https://www.r-project.org
RStudio v1.0.153	RStudio Team (2016)	http://www.rstudio.com/
Spyder - The Scientific PYthon Development EnviRonment v2.3.5	Github repository	www.pythonhosted.org/spyder / https://github.com/spyder-ide/spyder
Numpy v1.8.1	van der Walt et a (2011)	http://www.numpy.org
Scipy v0.11.0b1	Jones et al (2001)	https://www.scipy.org
Matplotlib v1.5.1	Hunter (2007)	https://matplotlib.org

ggplot2 v2.2.1	Wickham (2009)	http://ggplot2.org
gridExtra	Auguie (2017)	https://cran.r-project.org/web/packages/gridExtra/index.html
dplyr v0.7.4	Wickham et al (2017)	https://cran.r-project.org/web/packages/dplyr/index.html
tidyr	Wickham and Henry (2017)	https://cran.r-project.org/web/packages/tidyr/index.html
lme4 v1.1-14	Cran repository	https://cran.r-project.org/
Nlme v3.1-131	Cran repository / Pinheiro et al (2017)	https://cran.r-project.org/web/packages/nlme/index.html
WRS2 v0.3-2	Cran repository	https://cran.r-project.org/
WRS	R forge repository	http://R-Forge.R-project.org
Key components of virtual reality set up		
Cylindrical polystyrene treadmill	Graham Sweet Studios	Custom made *
Silicon tubing	NResearch	Cat# TBGM101
Torus shaped screen	Talbot Designs Limited	Custom made *
Rotary encoder	Pewatron	Cat# E6-2500-472-IE
Disposable Straight Feeding Needle, 18 Gauge, 50.8 mm	Harvard apparatus	Cat# 598636
1Tube Normally Closed Full Opening Pinch Valve	NResearch	Cat# 225PNC1-21
Arduino Due	RS Components	Cat# 769-7412
L-shaped head post	3D Creations Lab	Custom made *
Feeding needle holder	3D Creations Lab	Custom made *
CoolDrive one single valve driver	NResearch	Cat# 225D1X180
Head clamp	Thor Labs	Custom made *
Enhanced aluminium coated mirror, 140mm diameter x 6mm thick	Knight Optical Ltd	Custom made *
Angular amplification mirror	3D Creations lab	Custom made *
Other		
Alpro Dairy Free Fresh Milk Alternative Soya, Original	Sainsbury’s	NA

* Custom designs will be shared upon request

REFERENCES

Atasoy D, Aponte Y, Su HH, Sternson SM. *J.* A FLEX switch targets Channelrhodopsin-2 to multiple cell types for imaging and long-range circuit mapping. *Neurosci. 2008 Jul 9. 28(28):7025-30.* 10.1523/JNEUROSCI.1954-08.2008PubMed 18614669

Baptiste Auguie (2017). gridExtra: Miscellaneous Functions for "Grid" Graphics. R package version 2.3. https://CRAN.R-project.org/package=gridExtra

Bourke, P. D. and Felinto, D. Q. (2010), Blender and Immersive Gaming in a Hemispherical Dome , Global Science & Technology Forum (GSTF).

G. van Rossum, Python tutorial, Technical Report CS-R9526, Centrum voor Wiskunde en Informatica (CWI), Amsterdam, May 1995

John D. Hunter. Matplotlib: A 2D Graphics Environment, Computing in Science & Engineering, 9, 90-95 (2007),DOI:10.1109/MCSE.2007.55 (publisher link)

Jones E, Oliphant E, Peterson P, *et al.* SciPy: Open Source Scientific Tools for Python, 2001- , http://www.scipy.org/ [Online; accessed 2017-12-19].

Linkert, M., Rueden, C. T., Allan, C., Burel, J. M., Moore, W., Patterson, A., Loranger, B., Moore, J., Neves, C., MacDonald, D., Tarkowska, A., Sticco, C., Hill, E., Rossner, M., Eliceiri, K. W. and Swedlow, J. R. (2010), 'Metadata matters: Access to image data in the real world', Journal of Cell Biology 189 (5), 777–782.

Murray AJ[1], Sauer JF, Riedel G, McClure C, Ansel L, Cheyne L, Bartos M, Wisden W, Wulff P. Parvalbumin-positive CA1 interneurons are required for spatial working but not for reference memory. Nat Neurosci. 2011 Mar;14(3):297-9. doi: 10.1038/nn.2751. Epub 2011 Jan 30.

Pinheiro J, Bates D, DebRoy S, Sarkar D and R Core Team (2017). _nlme: Linear and Nonlinear Mixed Effects Models_. R package version 3.1-131, <URL: https://CRAN.R-project.org/package=nlme>.

R Core Team (2013). R: A language and environment for statistical computing. R Foundation for Statistical Computing, Vienna, Austria. URL http://www.R-project.org/

RStudio Team (2016). RStudio: Integrated Development for R. RStudio, Inc., Boston, MA URL http://www.rstudio.com/.

Schindelin, J., Arganda-Carreras, I., Frise, E., Kaynig, V., Longair, M., Pietzsch, T., Preibisch, S., Rueden, C., Saalfeld, S., Schmid, B., Tinevez, J.-Y., White, D. J., Hartenstein, V., Eliceiri, K., Tomancak, P. and Cardona, A. (2012), ‘Fiji: an open-source platform for biological-image analysis’, Nature Methods 9 (7), 676–682.

Stéfan van der Walt, S. Chris Colbert and Gaël Varoquaux. The NumPy Array: A Structure for Efficient Numerical Computation, Computing in Science & Engineering, 13, 22-30 (2011), DOI:10.1109/MCSE.2011.37 (publisher link)

H. Wickham. ggplot2: Elegant Graphics for Data Analysis. Springer-Verlag, New York, 2009.

H. Wickham, Romain Francois, Lionel Henry and Kirill Müller (2017). dplyr: A Grammar of Data Manipulation. R package version 0.7.4. https://CRAN.R-project.org/package=dplyr

H. Wickham and L. Henry (2017). tidyr: Easily Tidy Data with 'spread()' and 'gather()' Functions. R package version 0.7.2. https://CRAN.R-project.org/package=tidyr

EXPERIMENTAL MODEL AND SUBJECT DETAILS

All animal experiments were carried out in compliance with protocols approved by the Animal Welfare and Ethical Review Board (AWERB) of the University of Edinburgh School of Medicine and Veterinary Medicine and under a UK Home Office Project License. Male and female mice, aged 7 – 12 weeks, were used for all experiments. Mice were randomly allocated to experimental groups. To selectively manipulate the activity of stellate cells in layer 2 of the MEC, a transgenic mouse line ($Sim1^{Cre}$) was used that enabled genetic access to layer 2 stellate cells as described in Sürmeli et al (2015). In this mouse line, Cre expression is controlled by the Single minded homolog-1 (Sim1) promoter. The line was generated by GenSat and obtained from MMRRC (strain name: Tg(Sim1cre)KJ21Gsat/ Mmucd). It was maintained by crossing male $Sim1^{Cre}$ mice with female C57/BL6jCrl wild-type mice (Charles River). Animals were kept in standard cages containing toys and wheels, and were maintained on a 12:12 hour reverse light:dark schedule (with lights off at 7am). Animals were group housed (up to 4 mice per cage post weaning) until surgeries for head post implantation and then were single housed. Mice had free access to food and water until the beginning of food deprivation described below. All animals had access to a running wheel in their home cages, except for two days of postoperative recovery. Small cardboard tubes were also placed in each cage after postoperative recovery for environmental enrichment.

METHOD DETAILS

Virtual-reality system. Mice were attached to a custom designed head clamp (Thor Labs) situated above a cylindrical polystyrene treadmill (Graham Sweet Studios) that allows movement in two directions; forward and back. Linear virtual tracks designed in Blender3D (Blender.com) were projected onto a torus shaped screen (Talbot Designs Limited) that surrounded the animal. The system was designed so that the convex mirror spreading the image over the screen has a

surface profile that creates a linear relationship between the incident angle and the deflection angle across its entire surface. The animal is placed at one intersection point of the torus, while the convex mirror is placed at the other intersection point, thus creating a horopter. The resulting linear relationships along the horizontal and vertical axes between the projection of the virtual environment, and its perception in terms of movement across the animal's retina, avoid 'warping' or other unnatural movement of visual flow patterns across the animal's field of vision. A rotary encoder (Pewatron, E6-2500-472-IE) that monitors movement on the treadmill communicates with Blender3D, which was used to generate and update the virtual environment. A feeding tube (Harvard Apparatus) placed in front of the animal, and controlled by a normally closed, full opening pinch valve (NResearch, 225PNC1-21), was used to dispense soy-milk rewards (5-10 μl per reward). Stops in the reward zone for a pre-specified duration were detected by Blender3D, which interacted with the pinch valve via a microcontroller (Arduino Due, www.arduino.cc).

Virtual-track design. The standard virtual track was 2 m long with a 20 cm wide floor and 20 cm high walls. The floor had a wooden pattern and the proximal walls were decorated with a uniform repeating pattern of pseudo-randomized black and white dots. To allow consecutive trials to be clearly distinguished, the visible track was preceded and followed by black boxes of length 30 cm, such that locations -30 cm to 0 cm correspond to a black box, locations 0 to 140 cm to the visible track and locations 140 to 170 cm to a black box. On reaching the end of the track animals were 'teleported' back to the beginning to start another trial. To prevent the black box being used as a distal cue for locating the reward zone the visible the track was restricted to 50 cm ahead of the animal.

For beaconed trials a reward zone was located 60-80 cm from the start of the visible track. The reward zone was marked by cues consisting of black and green vertical stripes on the track walls. For non-beaconed and probe trials the cues were removed. On beaconed and non-beaconed trials, but not on probe trials, rewards were dispensed when mice stopped in the reward zone.

During standard trials every 1 cm travelled on the treadmill moves the animal an equivalent virtual unit (VU) along the visually projected track (1 cm: 1 VU). For gain modulation trials the conversion of physical movement on the treadmill to virtual movement was altered such that, the ratio of distance moved on the treadmill to distance traversed in the virtual linear track was either halved (gain x 0.5, 2 cm: 1 VU) or doubled (gain x 2, 1 cm: 2 VU).

For experiments in which the track length was increased, a series of tracks were generated (track 1 to track n) in which the distance from the end of the first black box to the start of the reward zone was described by the equation, $L_n = L_{n-1}*1.5$, where L_n and L_{n-1} are the distance from the start point to the reward zone for the current and previous tracks in the series. Track 1 was the standard track described above (length = 2m, L = 60 cm and gain = 1). The black boxes at the start and end of the tracks were 30 cm long for all tracks.

Head fixation. A head-post was attached to the skull of each mouse enabling it to be head fixed in the virtual reality set up. Surgeries were performed 10-13 days before beginning behavioral experiments. Mice were anesthetized using 1-3 % Isoflurane gas (Abbott Laboratories, IL) in oxygen. Analgesia was achieved by subcutaneous administration of Vetergesic (Ceva, UK) at a dose of 0.08 ml/kg bodyweight. The scalp was shaved and cleaned with antiseptic, the mouse was placed in a stereotaxic frame (Stoelting 51900) and maintained at 37°C using a

thermostatic heat blanket. The eyes were covered throughout surgery with hydrating opaque eye-gel (Viscotears, TX). A small region of the scalp (approx. 1.8 mm by 2.5 mm) was removed to expose lambda and bregma, the top of the skull was cleaned with isotonic saline and connective tissue removed with a scalpel blade. The skin was then glued to the skull with Vetbond (VetTech) leaving the top of the skull exposed for head-post implantation. A scalpel blade was used to cut ridges into the top of the skull. An L-shaped head post (Protolabs) was lowered onto the skull and fixed in place with Loctite glue. The exposed skull and post were then embedded in dental cement (Simplex rapid acrylic denture polymer, Associated Dental Products Ltd, UK). Following surgery mice were then placed on a heat bench for 30 - 60 minutes until they regained consciousness, and were given additional analgesic (Vetergesic) at 8-12 hours post surgery.

Viral injections. Adeno-associated viruses (AAVs) viruses were targeted stereotaxically to the MEC as described previously (Sürmeli et al., 2015). For behavioral experiments, viral injections were carried out during the head post surgery. Injections were via bilateral craniotomies targeted at 3.5 mm from bregma along the medial lateral axis and just above the transverse sinus along the anterior posterior axis. For each craniotomy, four injections were made along the dorsal-ventral axis of the MEC at intervals of approximately 200 μm. All four injections were performed using the same injection coordinates, but differed in the angle of the micropipette and depth of the injection site. For the first injection, which aimed to hit the dorsal most portion of the MEC, the micropipette was lowered at an angle of -16°. For each injection after this the angle was incrementally reduced by 3° so that the last injection was performed at an angle of -7° for targeting the ventral portion of layer 2. The depth of the injection sites was determined form the depth at which the micropipette bends once it hit the dura lining the surface of the brain. Approximate depths at which a bend should be seen in an 8 week old mouse are as follows for

each injection site: 1st injection: 1.8 mm; 2nd injection: 2 mm; 3rd injection: 2.2 mm; 4th injection: 2.4 mm.

Viruses were generated in-house and were titered by qPCR following expression in HEK cells. Viruses used where AAV-TeLC-GFP and AAV-GFP (obtained from Peer Wulf, Kiel University)(Murray et al., 2011), and AAV-FLEX-rev-ChR2(H134R)-mCherry (Addgene plasmid #18916). AAV-TeLC-GFP had a titer of 6.4 x 10^{12} cp/ml, AAV-GFP had a titer of 5.1 x 10^{12} cp/ml and AAV-FLEX-rev-ChR2(H134R)-mCherry had a titer of 9.45 x 10^{12} cp/ml. We injected 400-650 nl of AAV at each site. AAVs were generated with a chimeric 1/2 serotype as described previously (McClure et al., 2011).

Handling and habituation of experimental mice. To minimize behavioral variability the daily routine of the experimenter was made as consistent as possible (e.g. the same products were used for bodily hygiene, hands were cleaned before training in the same way etc). For one week after surgical procedures mice were handled daily for ten minutes, once in the morning and once in the afternoon for 5 and 10 minutes respectively. During each handling session mice were weighed to establish a baseline body weight for subsequent food deprivation.

Habituation to the apparatus began a minimum of a week after post-operative recovery. Mice were habituated to the head restraint on two consecutive days for sessions lasting 5 and 10 minutes respectively. Habituation sessions for each mouse were carried out at the same time of day as their subsequent training. During habituation sessions the virtual reality was on, but rewards were not dispensed. To minimize stress experienced by the mice, they were encouraged to walk voluntarily onto the treadmill before head restraint. Mice were handled for a further 5 minutes after habituation sessions.

Food deprivation. Mice began food deprivation after the final habituation session. Initially, mice were deprived of food for 24 hours. They were then fed 3.5 g of food daily at their allocated training time for 4 days before starting behavioral training. During behavioral training mice were fed between 3 and 4 g of chow 30 minutes after the end of their training session. The amount of food depended on their behavior during the training session and their current weight relative to their reference body weight. A daily growth curve was adjusted for each mouse according to its pre-experiment body weight (see (https://www.jax.org/jax-mice-and-services/strain-data-sheet-pages/body-weight-chart-000664)). The mouse's weight was then calculated as a percentage of the reference body weight and daily food was allotted to achieve a target bodyweight of 85 % of the reference body weight.

During behavioral training mice were trained in 5 day blocks followed by a 2 day break. For feeding on break days a specific protocol was followed. On the last training session of each block mice were fed 1 g more than their daily feeding amount. On the first day of the break mice were fed 0.5 g more than their daily feeding amount and on the last day of the break mice resumed their usual feeding amount.

Task rewards, criteria and stage progression. During behavioral experiments, mice were rewarded with 5 - 10 μl soy-milk when they stopped for a specified duration in the reward zone. A stop is detected during the experiment when the running speed of the mouse (calculated as the distance covered over the last 200 ms) drops below 0.7 cm/sec. On beaconed and non-beaconed trials mice were rewarded the first time they stopped in the reward zone. At the same time as the reward was released a 500 ms tone was played to reinforce the place-reward association. Rewards were not given on probe trials.

Each behavioral experiment was split into distinct stages. In stage 1, four out of five trials used beaconed tracks, and one out of five used non-beaconed tracks. In stage 2, probe trials replaced one out of every two trials on non-beaconed tracks. Mice progressed from stage 1 to stage 2 after a minimum number of days and when 75 % of trials in a session were successfully rewarded over two consecutive training days. For the experiment described in Figure 1, stage 1 was split into two sub-stages. In the first, the delay between stopping in the reward zone and receiving a reward was 0 ms. The delay was increased to 125 ms when mice completed 30 or more trials with ≥ 75 % of trials rewarded over two consecutive days. Once mice performed ≥ 75 % on a 125 ms delay, they graduated to stage 2 of the behavioral experiment. In this experiment, the minimum number of days to progress from stage 1 to stage 2 was 15. Following this set of experiments we decided the additional 125 ms delay was unnecessary given that probe trials could be used to assay performance and so in all other experiments the delay between stopping in the reward zone and receiving a reward was 0 ms throughout the experiment. In these experiments the minimum number of days to progress from stage 1 to stage 2 was 7.

Behavioral training. On each training day each mouse was removed from the holding room and taken to the experimental room 30 minutes prior to its allocated training time. The mice were handled for 5 - 10 minutes, weighed and then placed in a large cage or open field filled with toys and a wheel. Mice were then handled for a further 5 - 10 minutes before sessions on the virtual reality set up. Sessions lasted 30 - 40 minutes each. After training mice were handled again for 5 minutes before being placed in the large cage for a further 10 minutes. Mice were then returned to their home cages and the holding room before feeding.

For the experiments described in Figures 5 and 6 the experimenter was blind to the viral manipulation. For this, a colleague assigned each mouse in the experiment a number. Mice were then allocated training times according to their number. Typically the first mouse (mouse 1) began training between 10 and 11 am and the last no later than 7 pm. Allocation of mice was randomized so that an equal number of control and experimental mice were trained in the morning and afternoon. The experimenter was un-blinded only once behavioral training and initial histological analysis were complete. For the experiment described in Figure 5, a first histological analysis using a semi-quantitative scoring system was carried out before un-blinding, and a second analysis using the quantification of fluorescent images described below was carried out after un-blinding. Results from the two analyses were consistent with one another. The experiment described in Figure 6 used only the quantitative system for scoring of expression levels.

Histology. Following completion of behavioral experiments to evaluate effects of transducing stellate cells with AAV-TeLC-GFP and AAV-GFP, mice were anaesthetized with Isoflurane and given a lethal dose of sodium pentobarbital (Euthatal, Meridal Animal Health, UK). Tissues were fixed by transcardial perfusion of ice-cold phosphate buffered saline (PBS; Invitrogen) followed by 4 % paraformaldehyde (PFA; Sigma Aldrich) in 0.2 M phosphate buffer (PB; Sigma Aldrich). Brains were removed and left in 4 % PFA in 0.2 M PB overnight and then left in 30 % sucrose in PBS for two nights. Brains were stored at 4°C on a shaker and wrapped in foil at all times.

Brains were sectioned in the sagittal plane at 50 - 60 μm thickness with a freezing microtome and then floated in a solution of 0.2 % Triton X-100 (Sigma Aldrich) in PBS (PBS-T). Slices were permeabilised with 0.2 % Triton X-100 (Sigma Aldrich) in PBS (PBS-T) and blocked with 5 % normal goat serum (NGS) in PBS-T for one hour at room temperature. Sections were then

incubated for 1-2 hours in fluorescent Nissl stain (1:500; Neurotrace 640/660 or 530/610, Life Technologies) in PBS-T at room temperature. Slices were then washed in PBS-T 4 times for 15 minutes each before being mounted on polysine slides (Thermo Scientific, UK), embedded in mounting media (Mowiol) and cover-slipped. Sections from a subset of brains were tested for co-localization of reelin and calbindin with eGFP fluorescence (Supplemental Figure 4). Sections were rinsed in PBS 3 times and blocked for 1 h in 10 % NGS in PBS-T, before adding primary antibodies (anti-Reelin, raised in mouse, Millipore, 1:500; anti-Calbindin, raised in rabbit, Swant, 1:1000; in 10% NGS in PBST) and incubated overnight at 4°C. The next day, sections were washed in PBS 3 times, then incubated overnight at 4°C in secondary antibodies (goat anti-mouse Alexa-647 and goat anti-rabbit Alexa-405, both 1:500; Invitrogen in 1% NGS in PBST). They were then washed in PBS, mounted onto slides with Mowiol, and stored in the dark at 4°C until imaged.

Imaging. Imaging of labelled sections was carried out using either Nikon A1 or a Zeiss LSM800 confocal microscopes. Single plane tiled images of all the sagittal sections were taken using a EC Plan-Neofluar 5x/0.16 objective. Stacks of images containing the MEC were taken through 23 μm of the Z-axis (z-step of 3 μm) using a 20x Plan Apo VC 20x DIC N2 objective. Combinations of the laser wavelengths used were 647 nm, 555nm, 488 nm and 405 nm depending on the staining used. Images were subsequently adjusted and analysed in ImageJ. For each slice quantified, a control image was taken so that background fluorescence could be subtracted from the fluorescence measurements taken from the region of interest.

Electrophysiology experiments. We used stereotaxic surgeries as above to target AAV to the MEC of Sim1^{Cre} mice. Mice were injected either with a mixture of AAV-FLEX-rev-ChR2(H134R)-mCherry and either AAV-FLEX-TeLC-GFP or AAV-FLEX-GFP. Between 2 and 4 weeks after

injections, we prepared horizontal 400 μm thick brain slices containing the hippocampal dentate gyrus. We made patch-clamp recordings from granule cells and evaluated their responses to stimulation with 488 nm wavelength light following methods we have described previously (Sürmeli et al., 2015). Slices chosen for recording showed expression of eGFP and mCherry in the middle molecular layer of the dentate gyrus. Recording electrodes had a resistance of 3-5 MΩ and contained the following intracellular solution (mM): K Gluconate 130; KCl 10, HEPES 10, MgCl2 2, EGTA 0.1, Na2ATP 2, Na2GTP 0.3 NaPhosphocreatine 10, biocytin 5.4. The external aCSF had the following composition (mM): NaCl 124, NaH2PO4 1.2, KCl 2.5, NaHCO3 25, Glucose 25, CaCl2 2, MgCl2 1. Recordings were made in current-clamp mode from cells with series resistance ≤ 30 MΩ and with bridge-balance and pipette capacitance neutralization applied.

Object exploration tasks. Experiments compared Sim1^{Cre} mice with injection of either AAV-FLEX-TeLC-GFP or AAV-FLEX-GFP into the MEC. Behavioral testing started nine days after injections. Mice were placed in an opaque plastic chamber measuring 61cm x 61cm x 60cm, surrounded by black curtains equipped with salient high contrast distal visual cues (e.g. a large yellow bin bag, a feather duster). The positions of the distal cues remained consistent throughout experiments. The chamber was cleaned prior to testing and between sessions using 70% ethanol and the bedding redistributed evenly.

Over the first 4 days the animals were habituated to the testing box. Mice then received 4 testing sessions over 4 days: two object recognition sessions, and two object-location sessions. A session consisted of three 5 min exploration phases separated by 3 min

intervals: a habituation phase, a sample phase and a test phase. For each phase, the mouse was placed into the test box, and allowed to explore. Between phases the mouse was removed from the test box and placed in an opaque container while the floor and walls of the box and objects were cleaned with 70% ethanol and the box prepared for the next phase. At the end of a testing session the mouse was placed back into its home cage, and the test box and objects cleaned and prepared for the next mouse.

The phases of the object recognition task were structured in the following way. Habituation phase: the box contained no objects. Sample phase: the box contained 2 copies of the same novel object. Test phase: one object was replaced with a third identical copy, whilst the other was replaced with a novel object. The phases of the object location task were structured in the following way. Habituation phase: the box contained no objects. Sample phase: the box contained 2 copies of the same novel object. Test phase: one object was moved to a novel location. To ensure that changes in behavior were not a result of preference for a certain object or side of the arena, the location and choice of the objects were counterbalanced between treatment groups. For both tasks, the time spent exploring each object in the sample and tests phases was measured.

QUANTIFICATION AND STATISTICAL ANALYSES

Quantification of virus expression. Quantification of virus expression was carried out in FIJI. Confocal images were opened using the Bio-Formats package (Linkert et al., 2010). For each

hemisphere in each mouse, quantification was performed in 7 sagittal slices located 3 to 3.72 mm from the midline. First, using the image of the slice labeled with Neurotrace, the length of the MEC was outlined using the freehand tool in FIJI. The midpoint of the line was used to split the MEC into dorsal and ventral sections. Layer 2 of the MEC in the dorsal and ventral sections was then selected using the freehand tool. The mean pixel intensity of fluorescence in the GFP channel, which identifies virally transduced neurons, was then calculated for dorsal and ventral layer 2 using FIJIs measurement tool. This was carried out for each of the dorsal and ventral segments in each slice.

Expression of GFP outside of layer 2 was rare, but in some animals occasional labeled cell bodies were observed in layer 5a, which was identified on the basis of Neurotrace labeling. Because GFP expression in layer 5a was sparse, and to avoid contamination from axons of L2SCs, expression was quantified by counting the number of cell bodies expressing virus. Labeled cells were counted in each slice along for each hemisphere in each mouse

Analysis of virtual reality behaviours. Data collection, analysis and presentation was performed using custom scripts written in python 3.5 (www.python.org) using Numpy v1.8.1, Scipy v0.11.0b1 and Matplotlib v1.5.1 packages. Scripts were written using Spyder 2.3 (www.pythonhosted.org/spyder). For each trial the trial type, elapsed time, track location and running speed averaged over the previous 200 ms were recorded at approximately 60 Hz as outputs from Blender. Stopping locations were recorded as locations on the track at which the velocity was < 0.7 cm/s and that were > 1 cm from a previously recorded stop. For analysis of the location of stops the track was divided into 20 bins, and the mean number of stops calculated for each bin. For experiments using longer tracks the bin size remained at 10 cm and so the number of bins was increased.

To facilitate comparison between animals, z-scored stopping probabilities were calculated as follows. First, the stop times for a given session were shuffled. For a given animal on a given day we sampled 100 trials with replacement. We considered the track as a circle and added an angle drawn from a uniform random distribution to each set of stop locations for each trial. This procedure was repeated 1000 times to generate a shuffled data set. The shuffled data set was then used to calculate means and standard deviations for the probability of stopping in each bin. Z-scored stopping probabilities were then calculated for each bin such that z score = (Mean Stops - Mean Shuffled Stops)/(Shuffled STD).

A number of additional measures of general task performance were calculated. These included: the number of trials completed per training session; the number of rewards obtained per training day; the fraction of rewarded trial; the days to meet the criteria to graduate to stage two of the experiment.

For the experiment described in Figure 4, one of the mice in the GFP group became unwell on day 17 and at this point excluded from the experiment. Data from this mouse before this point were included in the reported analyses.

Electrophysiology analysis. Electrophysiology data were analyzed using IGORpro (Wavemetrics). Amplitudes of individual light-evoked EPSPs were measured and then the mean calculated for each neuron.

Analysis of object behaviors. For each task, object exploration was scored manually using the 'Multitimer' scoring system. An exploration was recorded when the mouse's nose was within 1 cm of the object and pointing directly at it (Vogel-Ciernia and Wood, 2014). Manual scores were confirmed by repeating the scoring using AnyMaze (http://www.anymaze.co.uk/) on video recordings of the mouse's exploration. To ensure results were not skewed by animals that did not explore, exclusion criteria was introduced. Animals were excluded from the dataset if they failed to explore an individual object for more than 5 seconds or all objects for more than 15 seconds during training phases, or if they failed to explore both objects for a total of 10 seconds in the test session.

To determine whether mice have a preference for a specific object (or object location) a discrimination index was calculated for each session. The discrimination index was defined as the difference between the exploration time of the novel object (or novel object location) and the exploration time of the familiar object, divided by the total exploration time. A score greater than zero indicates preference for the novel object or object location.

Statistical analysis. Statistical analysis was performed in R v3.30 (R Core Team, 2014). Scripts were written and run using RStudio 0.99.902 (RStudio Team, 2015; http://www.rstudio.com). Details of data distributions and tests are given in the main text and figures. When a measure was obtained repeatedly from the same animal, the mean for that animal was used for population level analyses unless indicated otherwise. Linear mixed effect models (LMEs) were

fit using lme 4 1.1-12 (Bates et al., 2015). Animal identity was included in models as a random effect and the variable of interest as a fixed effect. To evaluate significance of effects using LMEs, the model without the variable of interest (a reduced/null model) was compared to the model with the variable of interest using a likelihood ratio test. Because for experiments comparing effects of expression of GFP with TeLC-GFP (Figures 3 and 4) the distribution of the data appeared clearly non-normal, for analysis of these experiments we used robust statistical methods to compare groups (Wilcox, 2016). These were implemented in R using the packages WRS (https://github.com/nicebread/WRS) and WRS2 (v0.9-2 from cran.r-project.org). Comparisons of groups used the percentile bootstrap method. For independent groups differences between medians were evaluated using the R function medpb2 (in WRS2). For dependent groups the bootdpci function (in WRS) was used to compare 20% trimmed means. Results are reported using 10^5 bootstrap samples. Linear regression was performed using a least squares method that allows heteroscedasticity, implemented in the R function olshc4 (WRS package), with slopes compared using the R function ols2ci (WRS package). For multiple comparisons within an experiment reported p values were adjusted by the Benjamani and Hockberg method using the R function p.adjust.

DATA AND SOFTWARE AVAILABILITY

Data and code to reproduce the analyses reported in the paper will be made available at the time of publication via the University of Edinburgh DataShare repository (http://datashare.is.ed.ac.uk/). Analysis code will be made available via the Nolan Lab GitHub repository (https://github.com/orgs/MattNolanLab/).

Supplemental Movie 1 (SupplementalMovie.mp4). Relates to Figure 1.

The video shows a trained mouse performing four consecutive beaconed trials followed by a non-beaconed trial.

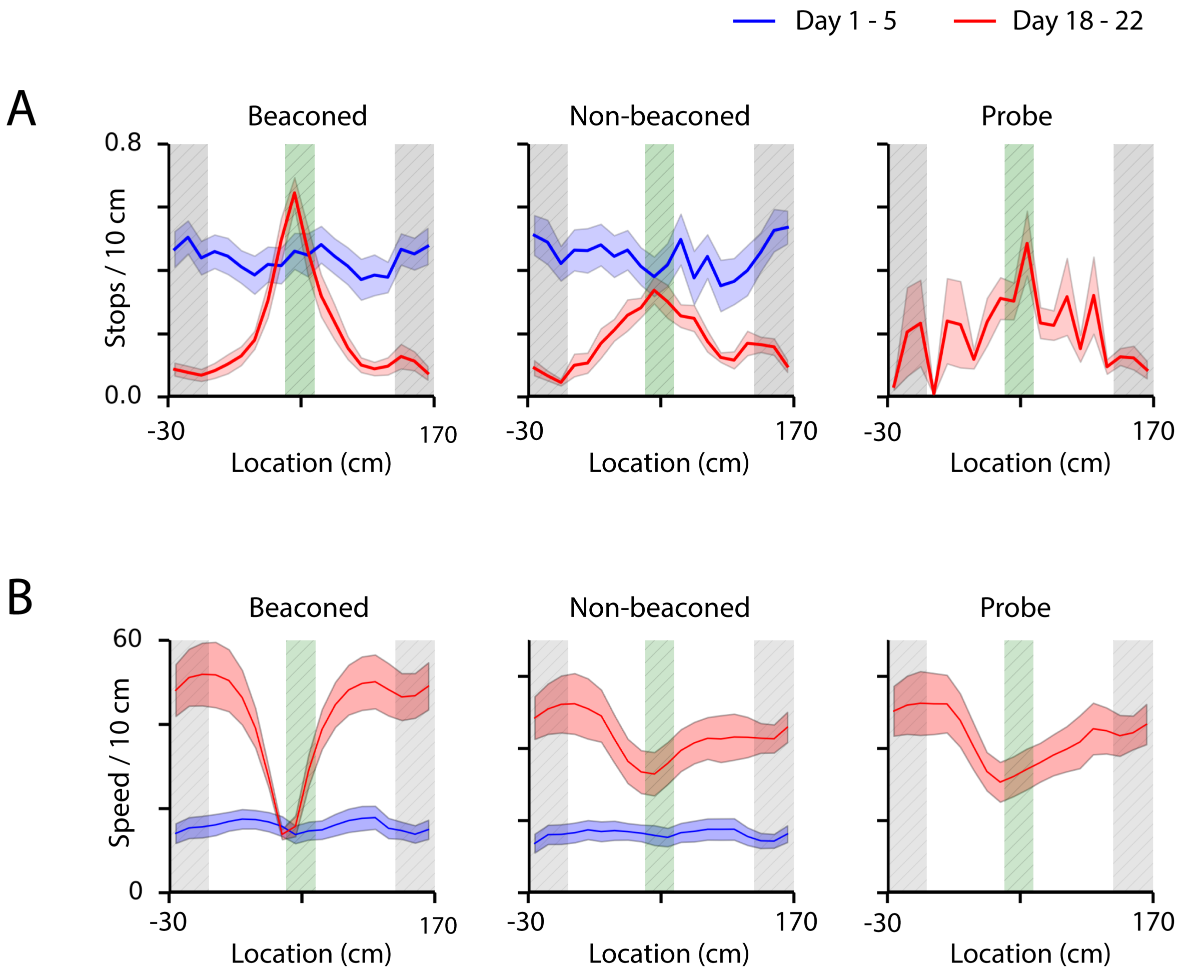

Supplemental Figure 1. Quantification of performance in a virtual location estimation task. Relates to Figure 1.

(A-B) Mean number of stops per 10 cm bin (A) and running speed (B) plotted as a function of location for beaconed, non-beaconed and probe trials on days 1-5 (blue) and 18-22 (red) of training. Shaded regions indicate the standard error of the mean.

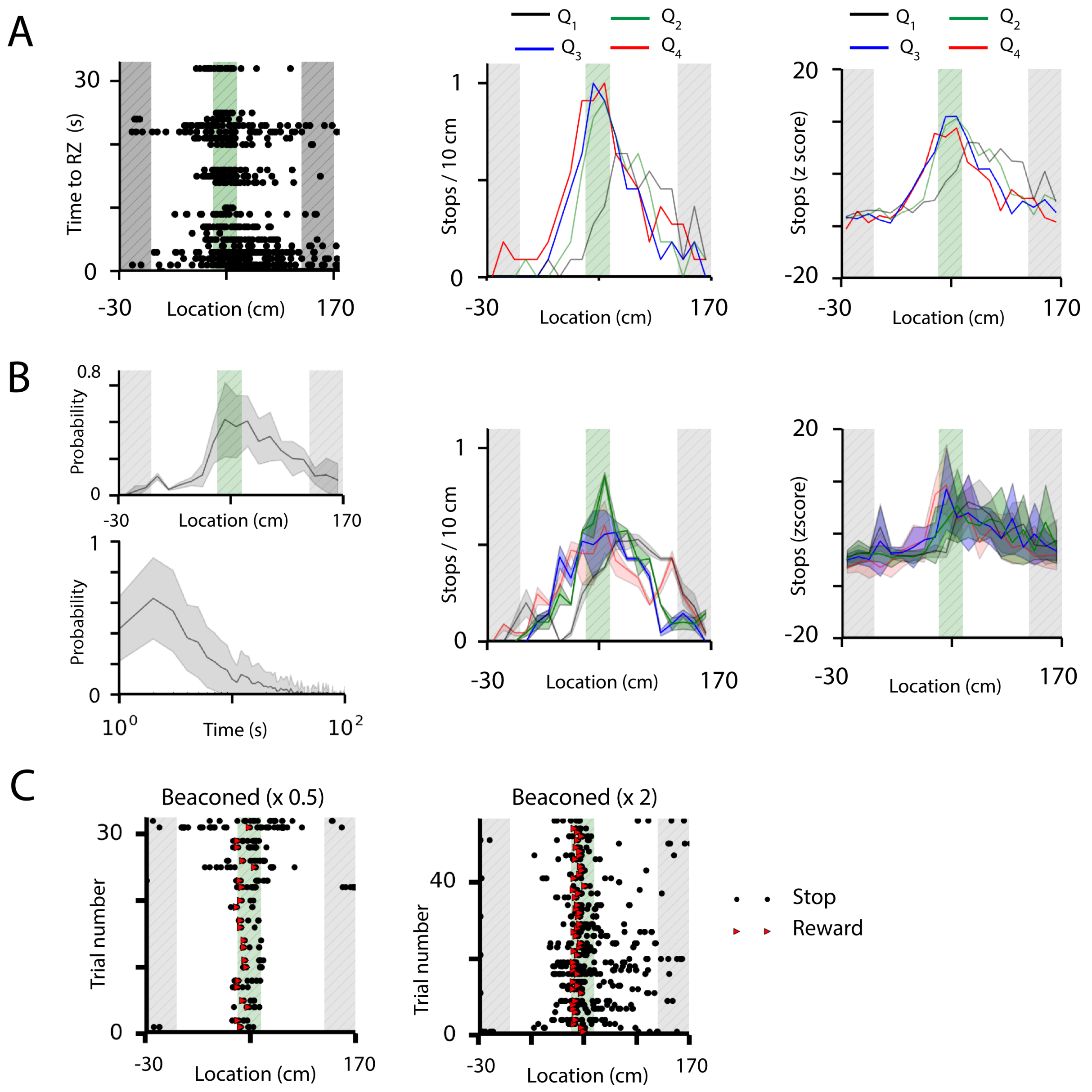

Supplemental Figure 2. Dependence of task performance on running speed and track gain. Relates to Figure 2.

A. Example plot for a single mouse of stops as a function of location with trials ordered on the y-axis according to time taken to reach the reward zone (left). The similar distribution of stop locations is consistent with use of a path integration mechanism rather than estimation of a fixed time interval. The number of stops in each 10 cm bin

(middle) and the z scored probability of stopping (right) are plotted for each quartile of the data sorted according to time taken to reach the reward zone, e.g. Q1 is the fastest 25% of trials, Q2 the next fastest, etc.

B. The distribution of stop locations (upper left) and stop times (lower left) for probe trials from all mice. Note that because the stop times were highly skewed they are plotted on a logarithmic axis. Consistent with stopping location being determined by a path integration mechanism, rather than a time counting mechanism, the mean coefficient of variation for stop times (1.18 ± 0.18) was substantially higher than for stop locations (0.45 ± 0.06; $p = 0.010$, paired t-test, $df = 4$, $t = -4.6$). In support of this interpretation, the group averaged time-ordered quartiles of mean stop probabilities (middle) and z scored stopping probabilities (right) did not appear to vary systematically (these panels show group data corresponding to the example data in A).

C. Examples of stopping locations on beaconed trials for the mice for which data on gain change trials is shown in Figure 2.

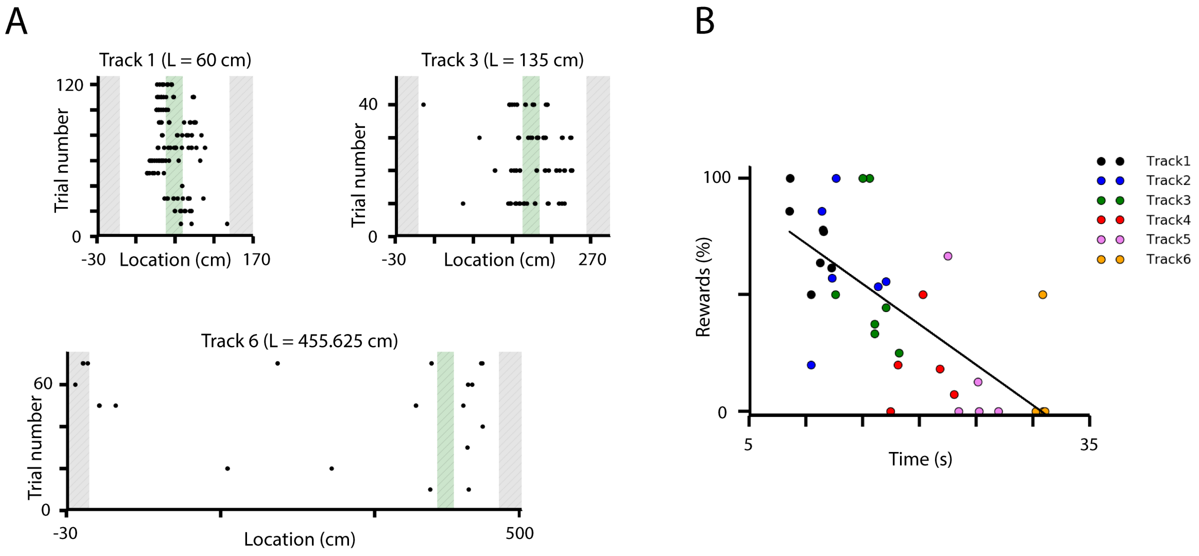

Supplemental Figure 3. Location estimates become inaccurate with increasing distance and time. Relates to Figure 3.

A. Examples of stopping locations on probe trials on tracks increasing in length. Note stop locations are centered in the reward zone on shorter tracks and become inaccurate with increasing length.

B. Percentage of correct probe trials as a function of average time to reach the reward zone for each animal on each track. Performance on different tracks is indicated by the colors. The line shows results of linear regression ($p = 4.6 \times 10^{-6}$ and $r = -0.70$).

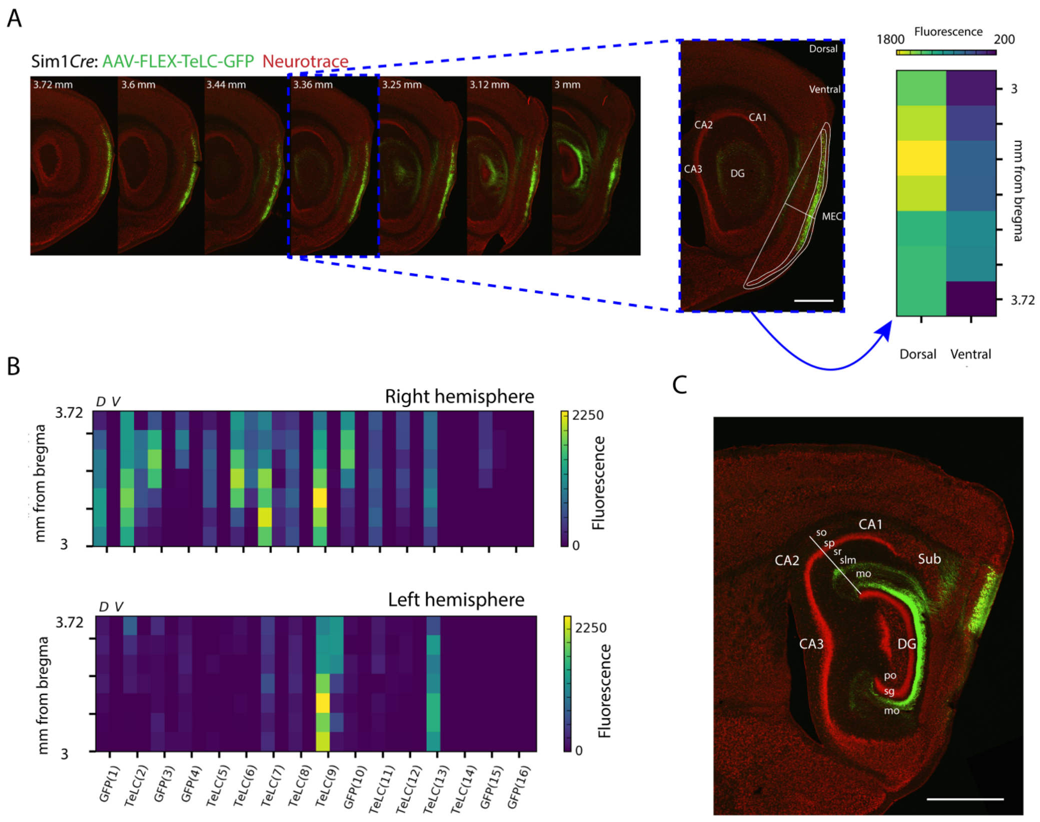

Supplemental Figure 4. Quantification of viral expression in layer 2 of the MEC. Relates to Figure 5.

A. Examples of a series of sagittal brain sections (lateral to medial) used to quantify TeLC-GFP / GFP expression (green). For each section a region of interest corresponding to layer 2 was drawn and fluorescence levels automatically calculated.

B. Quantification of TeLC-GFP / GFP expression for all animals. Height on the y-axis corresponds to the medio-lateral position of the section from which fluorescence was quantified. Dorsal (D) and ventral (V) expression levels are show separately for each animal.

(C) Expression throughout the hippocampal region. Note that labeling is present in the terminal fields of stellate cells in the dentate gyrus. Labeling is absent from CA1

indicating that entorhinal pyramidal cells do not express the virally delivered transgenes.

Scale bar is 1 mm.

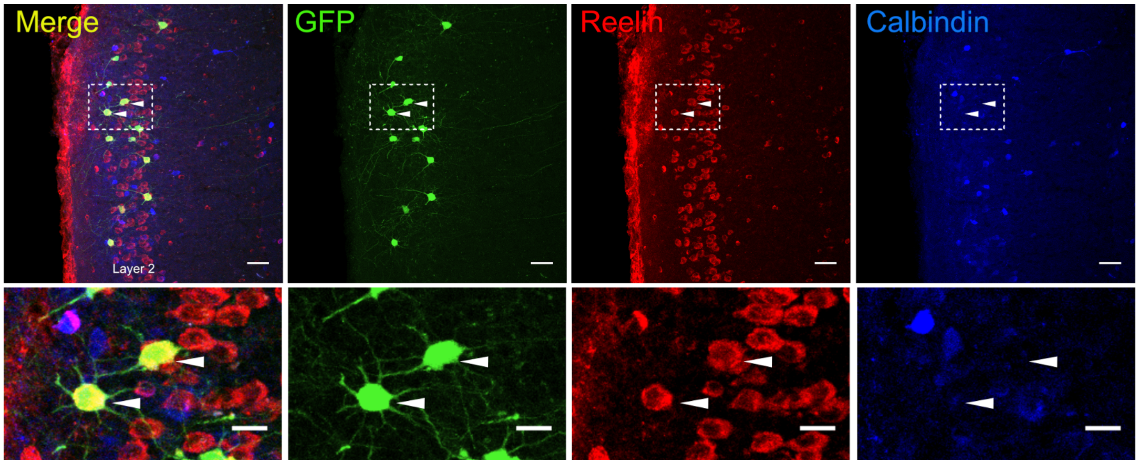

Supplemental Figure 5. Expression of TeLC is restricted to L2SCs. Relates to Figure 5.

Examples sagittal section containing MEC from a $Sim1^{Cre}$ mouse following injection of AAV-FLEX-TeLC-eGFP. The eGFP signal (green) is present in layer 2 neurons labeled with antibodies against the stellate cell marker reelin (red), but not neurons labeled with antibodies against the pyramidal cell marker calbindin (blue). We find similar labeling in all mice tested (n = 3). Selective Cre-dependent expression of transgenes in L2SCs is consistent with previous analysis of $Sim1^{Cre}$ mice (Sürmeli et al., 2015). Scale bars for upper panels are 50 μm and for lower panels are 20 μm.

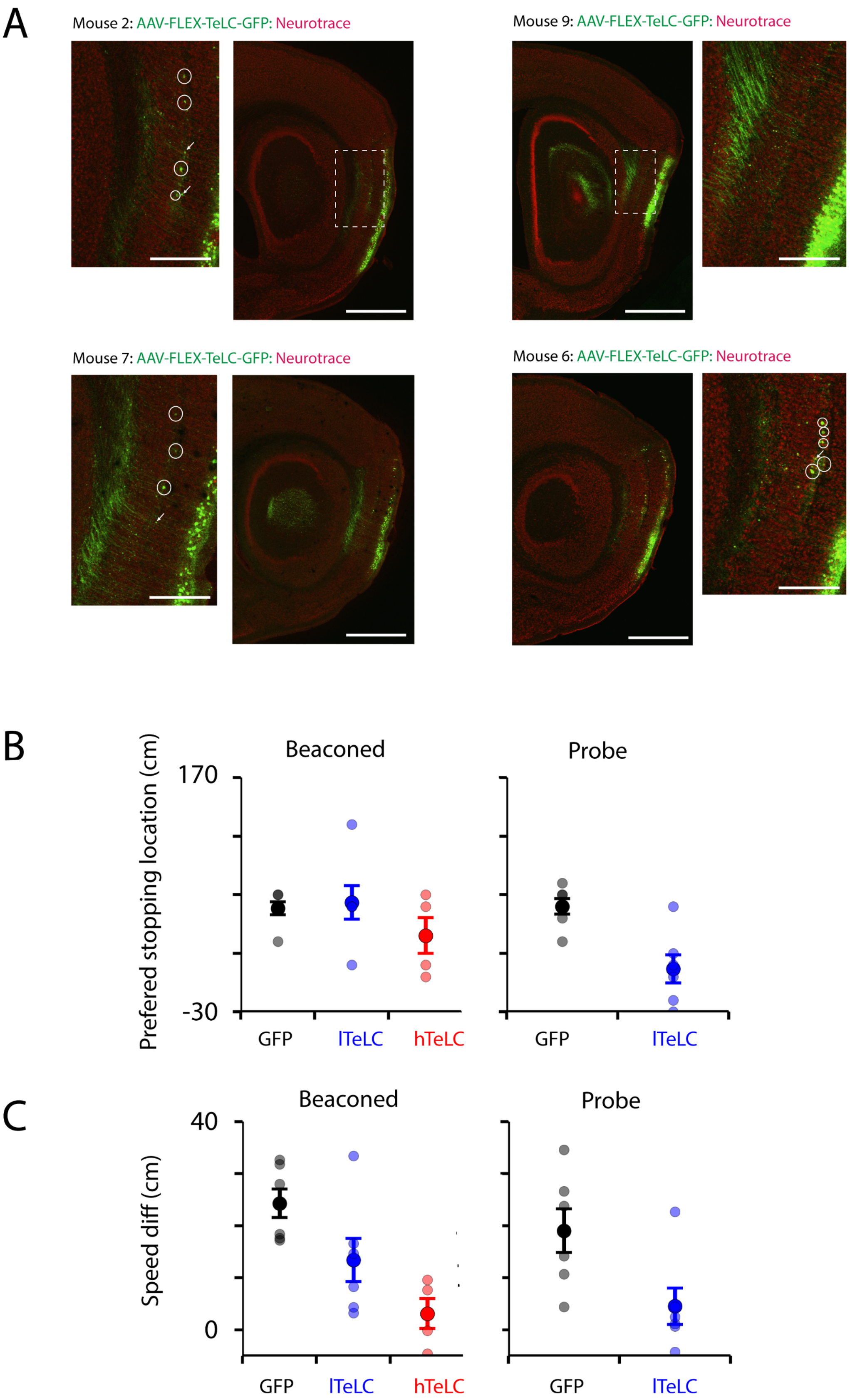
A
Mouse 2: AAV-FLEX-TeLC-GFP: Neurotrace
Mouse 9: AAV-FLEX-TeLC-GFP: Neurotrace
Mouse 7: AAV-FLEX-TeLC-GFP: Neurotrace
Mouse 6: AAV-FLEX-TeLC-GFP: Neurotrace
B
Beaconed
Probe
Prefered stopping location (cm)
170
-30
GFP
lTeLC
hTeLC
GFP
lTeLC
C
Beaconed
Probe
Speed diff (cm)
40
0
GFP
lTeLC
hTeLC
GFP
lTeLC

Supplemental Figure 6. TeLC-GFP expression in L5a and additional quantification of TeLC effects on location estimation. Relates to Figure 5.

(A) Sagittal sections from the brains of the four mice with highest expression of TeLC-eGFP. Each section is the one with the highest number of labeled cells (highlighted by circles) in layer 5a for that mouse. There were sufficiently few labeled cells in L5a that the total number across all sections could be counted (mouse 2: 16; mouse 6: 6; mouse 7: 10; mouse 9: 0). All of these mice, including the one without any detectable cells in L5a, failed to reach the performance threshold for graduate to the probe trial phase of the experiment. Mice with lower expression of TeLC-eGFP in L2 did not have detectable expression in L5a.

(B-C) Mean location of the most frequent binned stopping location (B) and mean difference between running speed at the start of the track and on entrance to the reward zone (C) during beaconed trials (left) and probe trials (centre). For each measure the beaconed and probe trials for mice in the lTeLC and eGFP groups are also compared directly (right). For the preferred stopping location neither the lTeLC or hTeLC groups differed from the GFP group (lTeLC: p = 0.78, percentile bootstrap corrected for multiple comparisons, test statistic = 0, 95% confidence interval [-3.5, 3]; hTeLC: p = 0.74, test statistic = 2.5, 95% confidence interval [-1, 6.5]), while lTeLC and GFP groups differed significantly during probe trials (p = 0.007, test statistic = 6, 95% confidence interval [2, 9]). For the difference in running speeds the hTeLC groups differed from the GFP group (p = 0.0, percentile bootstrap corrected for multiple comparisons, test statistic = 19.6, 95% confidence interval [9.0, 32.7]), but the lTeLC group did not (hTeLC: p = 0.11, test

statistic = 11.72, 95% confidence interval [-2.2, 25.6]), while lTeLC and GFP groups differed significantly during probe trials ($p = 0.03$, test statistic = 17.2, 95% confidence interval [1.6, 28.8]). For both measures performance of the lTeLC was impaired on probe compared with beaconed trials (preferred location: $p = 0.0053$, percentile bootstrap; speed difference $p = 0.0$).

REFERENCES

Bates, D., Machler, M., Bolker, B.M., and Walker, S.C. (2015). Fitting Linear Mixed-Effects Models Using lme4. J Stat Softw *67*, 1-48.

Linkert, M., Rueden, C.T., Allan, C., Burel, J.M., Moore, W., Patterson, A., Loranger, B., Moore, J., Neves, C., Macdonald, D., *et al.* (2010). Metadata matters: access to image data in the real world. J Cell Biol *189*, 777-782.

McClure, C., Cole, K.L., Wulff, P., Klugmann, M., and Murray, A.J. (2011). Production and titering of recombinant adeno-associated viral vectors. J Vis Exp, e3348.

Murray, A.J., Sauer, J.F., Riedel, G., McClure, C., Ansel, L., Cheyne, L., Bartos, M., Wisden, W., and Wulff, P. (2011). Parvalbumin-positive CA1 interneurons are required for spatial working but not for reference memory. Nat Neurosci *14*, 297-299.

Sürmeli, G., Marcu, D.-C., McClure, C., Garden, D.L.F., Pastoll, H., and Nolan, M.F. (2015). Molecularly defined circuitry reveals input-output segregation in deep layers of the medial entorhinal cortex. Neuron *88*, 1040-1053.

Vogel-Ciernia, A., and Wood, M.A. (2014). Examining object location and object recognition memory in mice. Curr Protoc Neurosci *69*, 8 31 31-17.

Wilcox, R.R. (2016). Introduction to robust estimation and hypothesis testing, 4th edition. edn (Waltham, MA: Elsevier).
