# Supplementary material for: Stellate Cells in the Medial Entorhinal Cortex Are Required for Spatial Learning
Source: Cell Rep. 2018 Jan 30;22(5):1313–24. doi: 10.1016/j.celrep.2018.01.005 (PMC5809635; doi:10.1016/j.celrep.2018.01.005)
Supplement: Document S2. Article plus Supplemental Information [file mmc3.pdf]

Cell Reports

Article

Stellate Cells in the Medial Entorhinal Cortex Are Required for Spatial Learning

Graphical Abstract

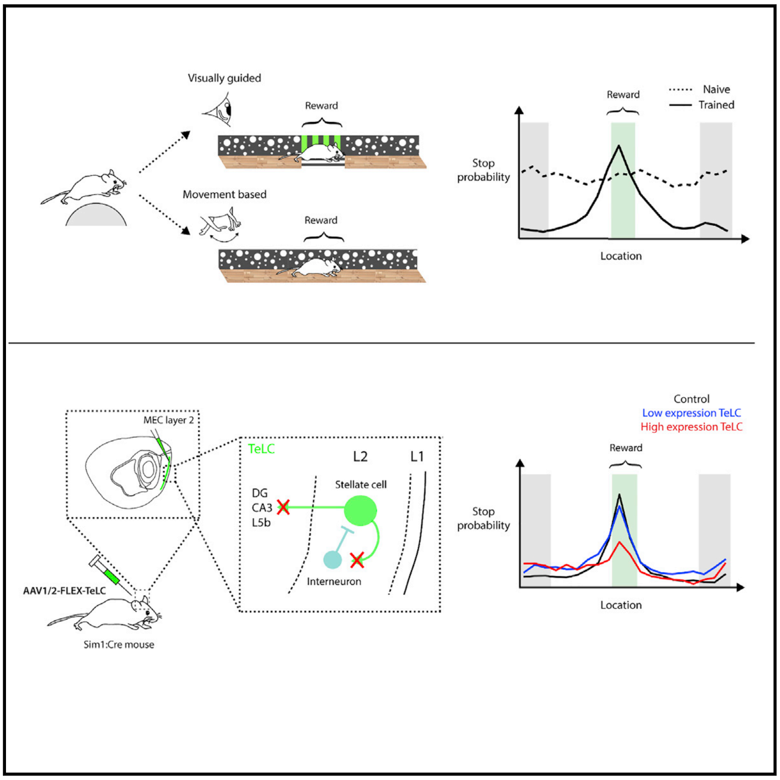

Authors

Sarah A. Tennant, Lukas Fischer, Derek L.F. Garden, ..., Christina McClure, Emma R. Wood, Matthew F. Nolan

Correspondence

mattnolan@ed.ac.uk

In Brief

Tennant et al. develop virtual reality tasks that dissociate beaconing and path integration strategies for location estimation. In combination with genetically targeted inactivation of synaptic output, the authors provide evidence for a critical role for entorhinal stellate cells in spatial learning.

Highlights

- Mice learn to estimate location by path integration and cue-based strategies
- Motor-related self-motion signals are used for path integration
- Accuracy of path integration decreases with distance
- Stellate cells in medial entorhinal cortex are required for spatial learning

Tennant et al., 2018, Cell Reports *22*, 1313–1324
January 30, 2018 © 2018 The Author(s).
https://doi.org/10.1016/j.celrep.2018.01.005

Cell Reports
Article

OPEN ACCESS
CellPress

Stellate Cells in the Medial Entorhinal Cortex Are Required for Spatial Learning

Sarah A. Tennant,[1] Lukas Fischer,[1] Derek L.F. Garden,[1] Klára Zsófia Gerlei,[1] Cristina Martinez-Gonzalez,[1] Christina McClure,[1] Emma R. Wood,[1] and Matthew F. Nolan[1,2,*]
[1]Centre for Discovery Brain Sciences, University of Edinburgh, Edinburgh EH8 9XD, UK
[2]Lead Contact
*Correspondence: mattnolan@ed.ac.uk
https://doi.org/10.1016/j.celrep.2018.01.005

SUMMARY

Spatial learning requires estimates of location that may be obtained by path integration or from positional cues. Grid and other spatial firing patterns of neurons in the superficial medial entorhinal cortex (MEC) suggest roles in behavioral estimation of location. However, distinguishing the contributions of path integration and cue-based signals to spatial behaviors is challenging, and the roles of identified MEC neurons are unclear. We use virtual reality to dissociate linear path integration from other strategies for behavioral estimation of location. We find that mice learn to path integrate using motor-related self-motion signals, with accuracy that decreases steeply as a function of distance. We show that inactivation of stellate cells in superficial MEC impairs spatial learning in virtual reality and in a real world object location recognition task. Our results quantify contributions of path integration to behavior and corroborate key predictions of models in which stellate cells contribute to location estimation.

INTRODUCTION

The ability to learn and update estimates of location during movement is central to theories of animal and artificial navigation (Durrant-Whyte and Bailey, 2006; McNaughton et al., 1996, 2006). In mammals, this core cognitive function may be achieved either using spatial cues, for example, through triangulation or beaconing strategies (Geva-Sagiv et al., 2015), or by path integration mechanisms, which generate representations of location from information about direction and speed of movement (Etienne and Jeffery, 2004). However, behavioral dissociation of path integration from cue-based navigation is challenging, as for many spatial behaviors investigated experimentally location estimates generated by any of several possible strategies may be sufficient for successful task performance. Indeed, while elegant experimental manipulations have directly tested mechanisms and roles of path integration in invertebrates (Collett et al., 1998, 2013; Wittlinger et al., 2006), the extent to which mammals use path integration strategies behaviorally is unclear, and whether the underlying neural substrates differ from those for cue-based location estimation is not known (Etienne and Jeffery, 2004; Jacob et al., 2017; Van Cauter et al., 2013; Winter et al., 2013).

The medial entorhinal cortex (MEC) contains multiple functional cell types that generate spatial representations that may be well suited to support behavioral estimation of location (Diehl et al., 2017; Hafting et al., 2005; Hardcastle et al., 2017; Solstad et al., 2008). These functional cell types include grid cells, which encode location through repeating hexagonally arranged firing fields (Hafting et al., 2005). Within the MEC, layer 2 has the greatest density of neurons with grid-firing fields (Sargolini et al., 2006), and grid firing has been localized to excitatory neurons with stellate and pyramidal morphology (Domnisoru et al., 2013; Schmidt-Hieber and Häusser, 2013; Sun et al., 2015). The stellate cells in layer 2 (L2SCs) have extensive local intralaminar connections (Beed et al., 2013; Couey et al., 2013; Fuchs et al., 2016; Pastoll et al., 2013), discrete projections to principal cells in layer 5b (Sürmeli et al., 2015), and long-range projections to the hippocampus (Schwartz and Coleman, 1981; Varga et al., 2010), making them well placed to coordinate and distribute grid and other spatial signals. Inactivation of L2SCs suppresses contextual fear conditioning (Kitamura et al., 2015). However, while grid cells encode representations of an animal's current location and theoretical models predict they may be used to plan trajectories to future locations (Burak and Fiete, 2009; Bush et al., 2015; Stemmler et al., 2015), it is not clear whether output from L2SCs is required for behaviors that require estimation of specific locations.

Because of the metric properties of their firing fields, grid cells have been proposed to encode the output of a path integration computation (McNaughton et al., 2006), and many theoretical models of grid firing perform path integration (Burgess and O'Keefe, 2011; Giocomo et al., 2011; Zilli, 2012). For example, continuous attractor network models (McNaughton et al., 2006), which have been proposed to account for grid firing based on connectivity between L2SCs and nearby interneurons (Couey et al., 2013; Pastoll et al., 2013), generate location estimates by integrating external spatial cues with velocity signals (Burak and Fiete, 2009; Fuhs and Touretzky, 2006; Guanella et al., 2007). Other models demonstrate that grid firing need not be the result of a path integration computation (Cheung, 2016; Kropff and Treves, 2008), and theoretical analyses suggest that the grid code may simply serve as a high-capacity spatial representation (Mathis et al., 2012; Sreenivasan and Fiete, 2011). In support of a path integration role, lesioning the MEC impairs measures of path integration in real world behavioral tasks (Jacob et al., 2017; Van Cauter et al., 2013; Winter et al., 2013); but, with this

This is an open access article under the CC BY license (http://creativecommons.org/licenses/by/4.0/).

OPEN ACCESS
CellPress

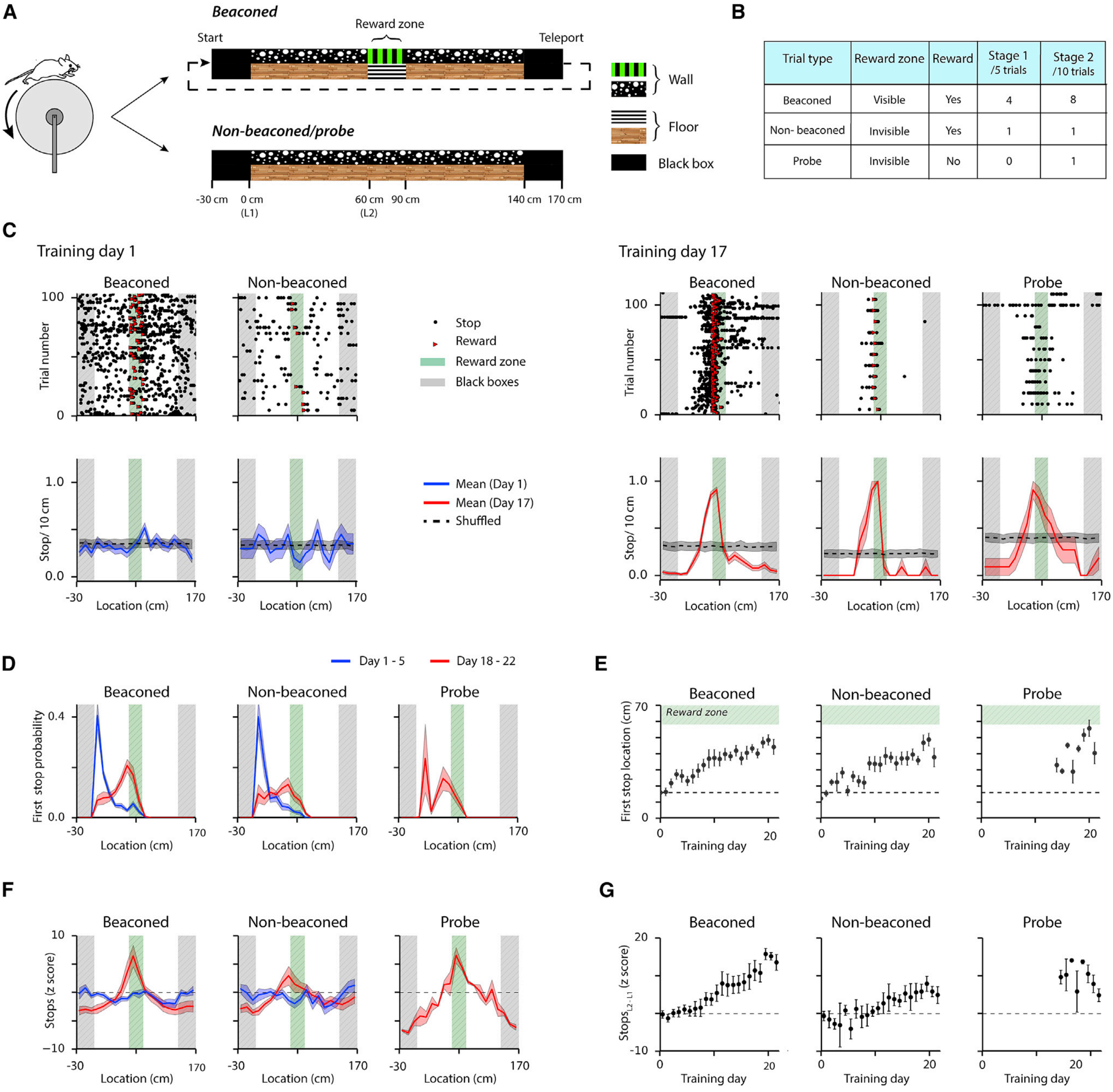

Figure 1. Mice Learn to Estimate Location Using a Path Integration Strategy

(A) Schematic of the virtual track used on beaconed trials (upper) or non-beaconed and probe trials (lower). The reward location is indicated by visual cues from stripes on the floor and walls of the track only on the beaconed trials.

(B) Configuration of trial types.

(C) Examples of raster plots of stopping locations as a function of track position, separated according to trial type, on day 1 (upper left) and on day 17 (upper right), and corresponding mean number of stops/10-s bin (lower plots). Stopping locations on the raster plots are indicated by dots, which are red for locations that triggered a reward, and otherwise are black. The mean numbers of stops are indicated by solid lines and shuffled means by dashed lines. The shaded bands around the means indicate the SEM.

(D) Average probability of the first stop on each trial as a function of binned track location for all mice (n = 8 mice) across days 1–5 (blue lines) and days 18–22 of training (red lines) separated according to trial type. Shaded regions indicate SEM. Bin width is 10 cm.

(E) Average first stop location as a function of training day for each trial type. The location of the first stop varied as a function of day for beaconed ($p < 2.2 \times 10^{-16}$, $\chi(1)^2 = 119.4$, likelihood ratio test) and non-beaconed trials ($p = < 2.2 \times 10^{-16}$, $\chi(1)^2 = 92.4$). There was no significant difference between the three trial types on days 18–22 ($p = 0.23$, $F(2,87) = 1.48$), 1-way repeated-measures ANOVA). Error bars are SEM (N = 8 mice for beaconed and non-beaconed trials and N = 6 mice for probe trials).

(legend continued on next page)

approach, it is not possible to distinguish roles of individual cell populations, and the contributions of surrounding brain structures are difficult to rule out. Moreover, to avoid confounding visual cues that might support landmark or beaconing strategies, path integration in real world conditions must be tested in the dark, which may impair representation by grid cells (Chen et al., 2016; Pérez-Escobar et al., 2016).

The hypothesized importance of L2SCs within circuitry that generates grid firing leads to the prediction that L2SCs are critical for spatial behaviors. The nature of the predicted contribution of L2SCs to spatial behaviors depends on the model considered. While path integration has been a major focus when investigating models of grid firing, in continuous attractor network models the same circuitry, depending on the availability of external inputs, generates grid patterns either through path integration or as a consequence of external spatial drive to the grid circuit (Guanella et al., 2007; Milford et al., 2010; Pastoll et al., 2013; Solanka et al., 2015). In these models, when external spatial cues are available, they are sufficient to dictate which neurons are active; but, when external spatial cues are not available, the models estimate location relative to the last spatial cue using path integration (Pastoll et al., 2013; Solanka et al., 2015). Analysis of robot systems suggests that this feature of continuous attractor circuits may be important for resolving navigational uncertainty (Milford et al., 2010). Given these considerations, if hypothesized continuous attractor networks within the MEC are the sole source of location estimates for hippocampal neurons important for spatial memory, then the inactivation of L2SCs should impair learning of cued and path integration-based estimates of location. On the other hand, if L2SCs are the source of velocity inputs to a downstream path integrator circuit, or if cue-based information reaches the hippocampus by routes that do not involve L2SCs, then inactivation of L2SCs would impair only path integration-based estimates of location. Finally, if the function of L2SCs is restricted to the identification of context (Kitamura et al., 2015), then inactivation of L2SCs should not affect estimation of location by either cued or path integration strategies.

Here we introduce methods for behavioral dissociation, in mice, of the linear component of path integration from cue-based localization strategies. We demonstrate that virtual reality-based behaviors can probe path integration strategies while avoiding confounds from spatial cues present in real world experiments. We find that mice successfully learn to use motor-related information to locate rewards using a path integration strategy, although with accuracy that decreases rapidly as a function of distance, unlike that of cue-based strategies. To investigate roles of L2SCs, we inactivated their outputs by the expression of tetanus toxin light chain (TeLC) (Murray et al., 2011). We found that this disrupts the adoption of both path integration and cue-based behavioral strategies. Consistent with hypothesized spatial roles of L2SCs, we also found that, in real-world experiments, the inactivation of L2SCs impairs the recognition of object locations, but not recognition of novel objects. Our results provide quantitative constraints for models that aim to account for mammalian path integration, and they implicate L2SCs as a critical component of the neural circuitry for cue- and path integration-based spatial learning.

RESULTS

A Behavioral Task for Quantitative Investigation of Cue- and Path Integration-Based Estimation of Location

To be able to selectively investigate neural mechanisms for beaconing and path integration, we developed a behavioral test that, depending on the task configuration, can be effectively solved either using local cues or by estimating location from self-motion signals, but in which potentially confounding external spatial cues are not available (Figure 1A; Movie S1). We trained mice to stop at a defined location on a virtual linear track to receive rewards. The virtual track had clearly identifiable start and end zones connected by a corridor that, apart from the clearly marked reward zone, did not contain any location-specific cues. In contrast to real world experiments, in which the distant end of a track could be used as a cue to estimate location, the end of the virtual track was not visible from the reward zone and, therefore, could not be used as a cue. In the first phase of training, the location of the reward zone was clearly indicated to the mouse using local visual cues on 4 of every 5 trials (beaconed trials) (Figure 1B). On the fifth trial, the visual markers for the reward zone were absent, but stopping within the zone was rewarded (non-beaconed trials). After a pre-determined training period, and if mice passed a performance criterion (see the Supplemental Experimental Procedures), every second non-beaconed trial was replaced with a probe trial. On probe trials, the visual cue was absent and no reward was delivered. Performance on non-beaconed and probe trials can be used to test whether mice estimate their position on the track using a path integration strategy, while probe trials also enable search strategies to be investigated.

We asked if mice locate the reward zone using a path integration strategy. On the first day of training, mice often received rewards by stopping in the reward zone, but there was no apparent spatial organization to their stopping behavior (Figure 1C). With training, the behavior of the mice changed, such that on leaving the start of the track mice ran, typically without stopping, to a region close to the reward zone, at which point they advanced at short intervals until they obtained a reward (Figure 1C). This change in behavior was readily observed in all mice as an increase in the distance from the start zone to the location of the first stop (Figures 1C–1E), from location-dependent changes in the probability of stopping (Figure S1A), and by a reduction in running speed as the animal approached the reward zone (Figure S1B). To enable quantitative comparison of stopping

(F) Mean *Z* scored probability of stopping as a function of binned track location for all mice (N = 8) across days 1–5 (blue lines) and days 18–22 of training (red lines) separated according to trial type. Shaded regions indicate SEM. Bin width is 10 cm.

(G) Spatial stopping behavior, quantified by the difference between the z score at the start of the track and at the entrance to the reward zone, plotted as a function of training day for each trial type. The difference varied as a function of day for beaconed ($p < 2.2 \times 10^{-16}$, $\chi(1)^2 = 142.5$, likelihood ratio test) and non-beaconed groups ($p = 7.4 \times 10^{-10}$, $\chi(1)^2 = 37.9$). Probe trials on days 18–22 did not differ from non-beaconed trials ($p = 0.78$, $F(1,50) = 0.08$).

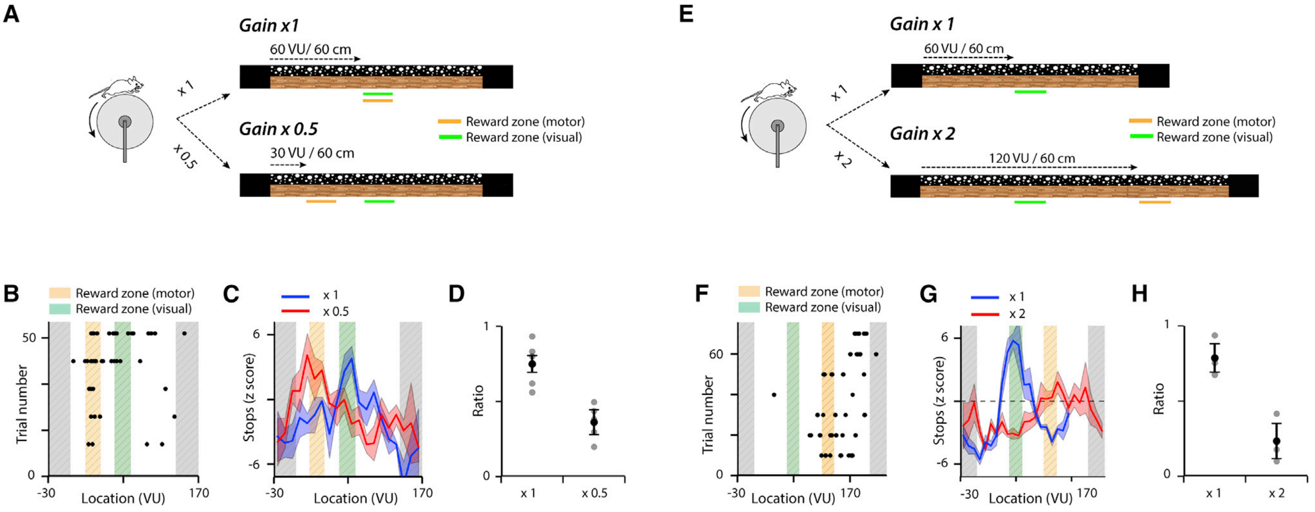

Figure 2. Path Integration Uses Motor-Related Movement Signals

(A and E) Schematic of track designs used to test a decrease (A) or an increase (E) in the gain between motor and visual reference frames. For standard trials, for every 60 cm mice run on the treadmill, the visual track moves 60 virtual units (VU). On reduced gain trials, for every 60 cm mice run, the visual track moves 30 VU. For increased gain trials the visual track moves 120 VU for every 60 cm mice run.

(B and F) Example plots of stop locations from single mice for trials in which the gain between treadmill movement and visual update of the track is reduced by 0.5 (B) or increased by ×2 (F). The trial number refers to all trials, but for clarity only data from gain change trials are shown.

(C and G) Average of Z scored stop locations across all mice for control probe trials (×1) and trials on which the gain is reduced (C) or increased (G). Averaged data are plotted as ± SEM (N = 5 mice for ×0.5 gain, N = 4 mice for ×2 gain).

(D and H) To quantify the effects of the gain change we compared, for each trial type, the ratio of stops in the location of the reward zone in the visual reference frame (orange) to the sum of the number of stops in the reward zone in the visual and motor reference frames (green). The ratio is modified by reducing ($t(4) = 3.7$, $p = 0.021$, paired t test) (D) or increasing gain ($t(3) = 6.5$, $p = 0.0073$) (H). Error bars indicate SEM.

Thus, on trials with reduced gain (B–D), or increased gain (F–H), stops occur in anticipation of the reward zone location in the motor reference frame.

strategies between animals, we calculated Z scored stopping probabilities by normalizing the mean probability of stopping at a given location to the mean and SD predicted by shuffled datasets (see the Supplemental Experimental Procedures). The distribution of stops in naive animals was similar in the experimental and shuffled datasets, whereas in trained animals the probability of stopping on the first part of the track was reduced and immediately before the reward zone was increased, relative to the shuffled data (Figures 1C, 1F, and 1G). Strikingly, this spatially selective stopping behavior was maintained on both non-beaconed and probe trials (Figures 1C–1G; Figures S1A and S1B). Because on these trials visual cues that might indicate the correct stopping location were absent, and on probe trials cues associated with the dispensing of rewards were also not available, these data indicate that mice solve the task using a path integration strategy. On the probe trials, mice typically stopped near the start of the hidden reward zone and continued to stop at short intervals until they reached the end of the hidden reward zone, at which point trained mice typically ran continuously to the end of the track to initiate a new trial (Figure 1C), suggesting that mice may also use path integration strategies to estimate the length of the reward zone.

Path Integration-Based Estimates of Location Update Using Self-Motion Signals

In principle, path integration can be achieved by updating location estimates using either visual (e.g., optic flow) or self-motion (e.g., proprioceptive feedback and motor efference) signals (Etienne et al., 1996; Raudies et al., 2016). To distinguish between these possibilities, we altered the relationship between treadmill movement and update of the visual projection of the track during probe trials. We found that, on these gain manipulation trials, mice continue to stop at a location predicted by the treadmill movement rather than by the visually perceived track movement (Figures 2A–2H). Thus, when the rate of update of the visual projection of the track was halved (Figures 2A–2D), or doubled (Figures 2E–2H), the first stop location and peak average stop location were just ahead of the reward zone predicted by the treadmill position. It is possible that animals use estimates of time instead of, or as well as, distance to complete the task (Kraus et al., 2015). To address this, we examined stopping location as a function of average running speed. If mice were using elapsed time to estimate location, then the distribution of stopping locations should depend on running speed, as mice running faster would cover a greater distance. In contrast, we found little or no dependence of the first stop location on running speed (Figures S2A and S2B). Thus, mice appear able to solve the task using a path integration strategy based on self-motion cues.

Accuracy of Path Integration Decreases with Distance from Location Cues

Theoretical models predict that internal noise will result in errors in path integration that increase with distance traveled (Cheung and Vickerstaff, 2010). The extent to which such errors limit the ability of mice to estimate location by path integration mechanisms is unclear. We therefore trained mice, using tracks of

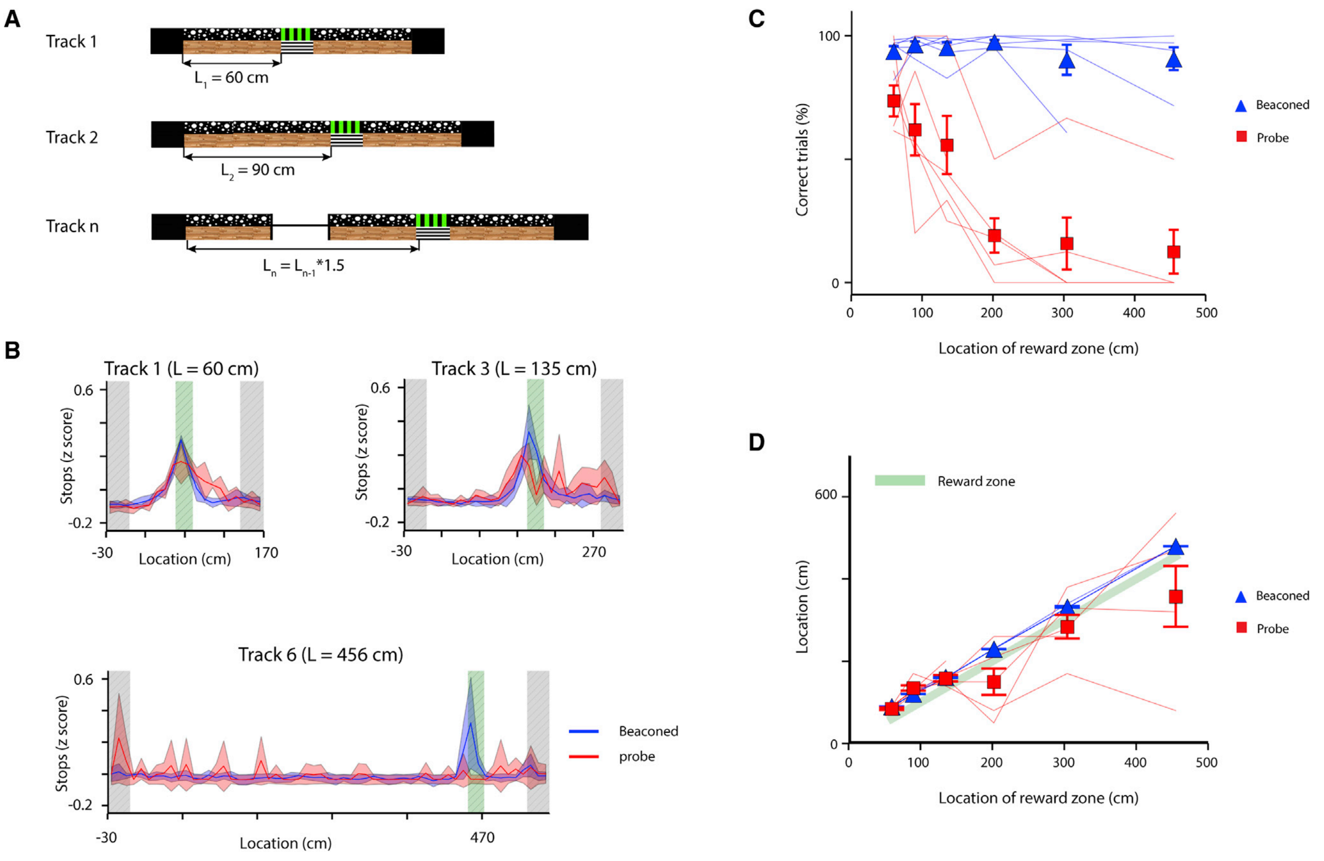

Figure 3. Path Integration Becomes Less Accurate with Increasing Distance

(A) For tracks of increasing length the distance from the start zone to the reward zone increases as indicated. The length of other parts of the track does not change.

(B) *Z* scored probability of stopping during probe trials as a function of location for three tracks of increasing length.

(C) Mean success rate at obtaining rewards as a function of distance from the start of the track to the reward zone separated according to trial type (N = 7 mice). The success rate depended on distance to the reward zone ($p = 8.74 \times 10^{-7}$, $F(1,60) = 30.1$) and accuracy of probe trials differed from beaconed trials ($p < 10^{-16}$, $F(1,60) = 139.3$). Success rate depended on distance to the reward zone for probe trial data ($\chi^2(1) = 22.8$, $p = 1.8 \times 10^{-6}$), but not for beaconed trial data ($\chi^2(1) = 1.87$, $p = 0.17$).

(D) Mean of the most frequent stop location plotted as a function of distance. The most frequent stop location depended on distance to the reward zone ($p < 10^{-16}$, $F(1,60) = 276.5$, 2-way repeated-measures ANOVA), but was independent of trial type ($p = 0.2$, $F(1,60) = 1.5$).

Error bars in (C) and (D) indicate SEM.

increasing length, to locate rewards at distances from 60 cm to >4 m from the start zone (Figure 3). After mice reached a criterion performance (see the Supplemental Experimental Procedures) on a track of a given length, we increased the distance to the reward zone by a factor of 1.5 (Figure 3A). We found that, as the length of the track was increased, mice continued to stop in the reward zone on a high proportion of beaconed trials (Figures 3B and 3C). Adaptation to the new reward zone location was usually apparent within the first 5 trials of the first session with the new track, indicating that visual cues can rapidly reconfigure the behavior. In contrast, the fraction of probe trials in which mice stopped in the reward zone dropped substantially as the track length increased (Figure 3C; Figure S3A). This steep drop in performance was also seen when success was evaluated as a function of time taken to reach the reward zone for tracks of different lengths (Figure S3B). Examination of stopping patterns indicated that, for intermediate-length tracks, the stopping locations were centered around the reward zone, even as the number of correct trials decreased (Figures 3B and 3D; Figure S3A). However, from trial to trial stop locations were variable and often were outside the reward zone, explaining the high number of errors (Figures 3B and 3D; Figure S3A). These observations argue against errors resulting from a residual memory for the previous stop location and are consistent with an accumulation of error in a path integrator system. Thus, in the absence of landmark cues to anchor path integration, the ability to accurately estimate location drops rapidly with distance from a known starting point in a manner that is consistent with performance of a noisy path integrator.

Blocking the Output from L2SCs Prevents Spatial Learning

Because superficial layers of the MEC are enriched with neurons that have spatial firing properties (Diehl et al., 2017; Hafting et al.,

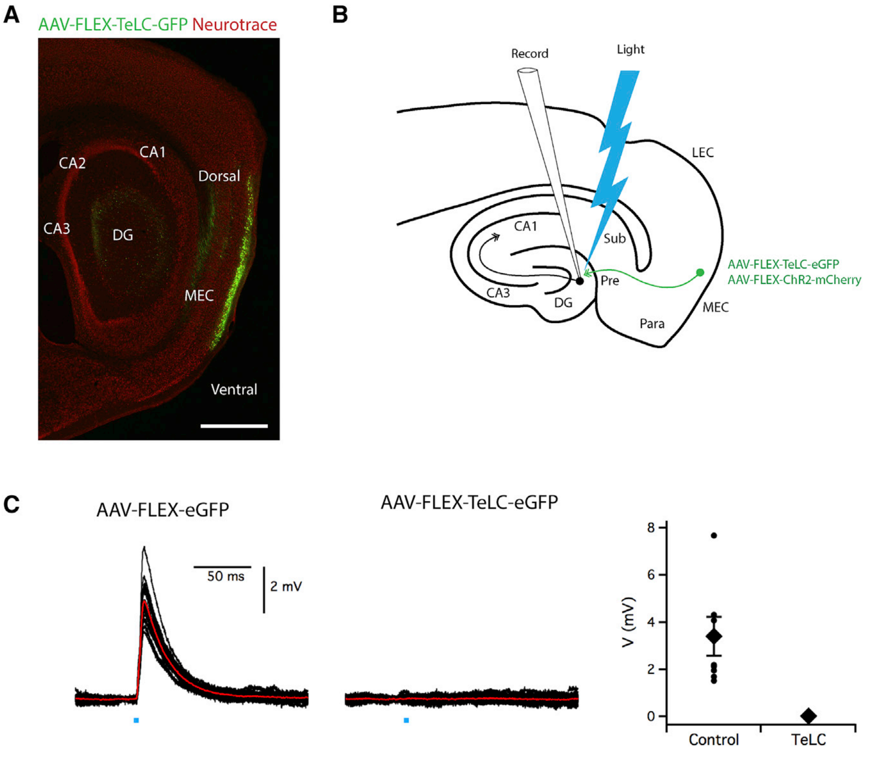

Figure 4. Targeted Expression of TeLC to L2SCs Abolishes Their Synaptic Output

(A) Example of a sagittal section from the brain of a $Sim1^{Cre}$ mouse following injection of AAV-TeLC-EGFP into the MEC. Scale bar, 1 mm.

(B) Schematic of experiment to test the effect of TeLC expression on synaptic output from L2SCs. AAV-FLEX-ChR2-mCherry and either AAV-FLEX-TeLC-EGFP or AAV-FLEX-EGFP were injected into the MEC of $Sim1^{Cre}$ mice. Synaptic output from L2SCs was evaluated by recording light evoked response of granule cells in the dentate gyrus.

(C) Examples of membrane potential responses of dentate gyrus granule cells to optogenetic activation of L2SCs expressing ChR2 and either GFP (left) or TeLC-EGFP (middle). Responses are present in all neurons from control animals (n = 10 neurons, N = 5 mice) and were absent in all neurons from animals expressing TeLC-EGFP (n = 8 neurons, N = 4 mice). The peak response was reduced by expression of TeLC-EGFP (right) (p = 0, percentile bootstrap comparison of control and TeLC-EGFP groups, test statistic = 2.125, 95% confidence interval [1.69, 4.29]). Circles are individual neurons, diamonds are the population average. Two neurons from two control mice were excluded from the plot and statistical analysis as they showed very large responses that reached action potential threshold preventing their quantification. Error bars indicate SEM.

2005; Hardcastle et al., 2017; Solstad et al., 2008), and as grid-firing patterns generated by neurons in the MEC are consistent with the output of a neural path integrator (McNaughton et al., 2006), we asked if neural circuitry in superficial MEC is required for learning of the beaconed or path integration components of the location estimation task. We focused on L2SCs, as the highest density of grid cells is in layer 2 (Sargolini et al., 2006), and L2SCs have grid-firing fields (Domnisoru et al., 2013). To be able to selectively manipulate L2SCs, we took advantage of $Sim1^{Cre}$ mice, which we found previously give specific genetic access to L2SCs (Sürmeli et al., 2015). To test the role of L2SCs, we blocked their synaptic output by injecting an adeno-associated virus (AAV) that expresses TeLC and EGFP conditionally on the presence of Cre (AAV-FLEX-TeLC-EGFP) (Murray et al., 2011) into the superficial MEC of $Sim1^{Cre}$ mice (Figure 4A). As a control, we used an AAV that expresses only EGFP (AAV-FLEX-EGFP). Expression of EGFP was restricted to L2SCs and was absent from the surrounding neurons (Figure 4A; Figures S4 and S5). To test whether expression of TeLC blocks SC output, we co-expressed channelrhodopsin 2 (ChR2) in L2SCs, to enable their optical activation, along with either TeLC-EGFP or EGFP (Figure 4B). When we recorded from downstream granule cells in the hippocampus, we observed synaptic currents following light activation of ChR2 in slices from mice expressing the control EGFP construct, but not in slices from mice expressing TeLC (Figure 4C). Thus, targeted expression of TeLC using $Sim1^{Cre}$ mice enables the block of synaptic output from L2SCs.

Does blocking output from L2SCs affect the ability of mice to learn a rewarded location? To address this, we injected the MEC of $Sim1^{Cre}$ mice with AAV-FLEX-TeLC-EGFP (n = 10) or AAV-FLEX-EGFP (n = 6). We trained the mice for 3 weeks in the virtual location estimation task, and then we sacrificed them in order to analyze the extent of expression of the viral transgenes (Figures S4 and S5). We found that the proportion of trials on which mice stopped in the reward zone was reduced for the TeLC-expressing mice compared to control mice. This was manifest as a delay to reach the criterion for the introduction of probe trials into the experiment (control: 7.33 ± 0.33 days, TeLC: 14.3 ± 1.69 days; p = 0.00069, percentile bootstrap comparison of control and TeLC groups, test statistic = −6.5, 95% confidence interval [−13,−2.5]). The delay depended on the extent of viral transduction in the TeLC group, but not in the control group (Figure 5A). Because expression and task progression were variable between animals, for further analysis we divided the mice, according to the extent of labeling of neurons in the dorsal MEC, into groups with high (hTeLC, n = 4) and low (lTeLC, n = 6) expression of TeLC (Figure 5A; Figure S4). Whereas all control mice reached the criterion for inclusion of probe trials within 9 days (7.33 ± 0.33 days), the lTeLC mice were delayed (10.5 ± 1.18 days; p = 0.019, test statistic = −3.5, 95% confidence interval [−6.5,−0.5]), and the hTeLC mice did not meet the criteria within the 19 days of the experiment. We note that, in 3 of 4 mice from the hTeLC group, we observed small numbers of labeled cells in L5a. Because very few cells were labeled in L5a in any animal and as the behavioral impairment was present in the mouse that had no detectable expression in L5a, expression of TeLC in deep layers is unlikely to account for the observed behavioral changes (Figure S6). Thus, these data indicate that output from L2SCs plays a key role in learning the

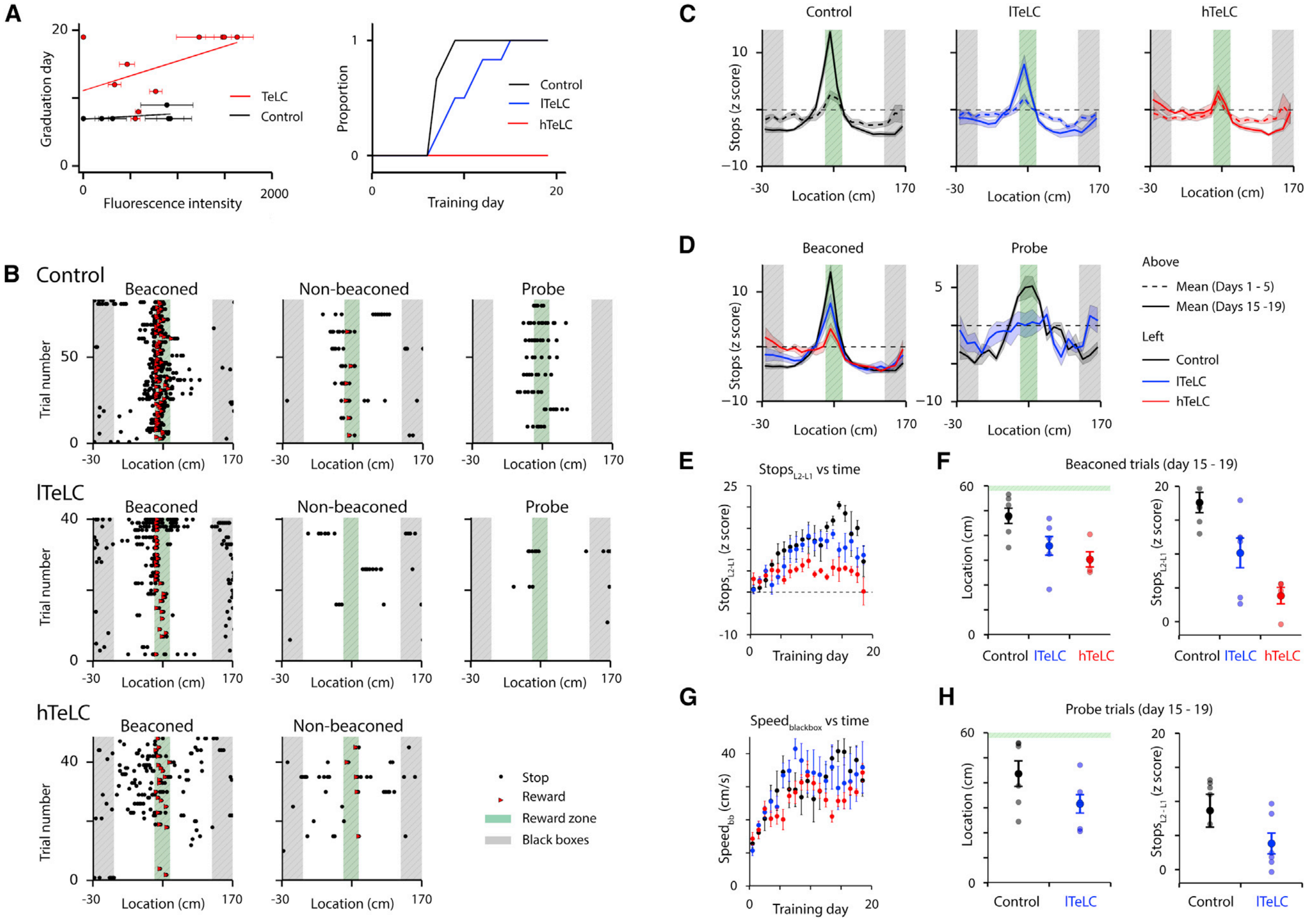

Figure 5. Inactivation of L2SCs Impairs Estimation of Location

(A) Day of the experiment on which each mouse from TeLC (N = 10) and control groups (N = 6) met the performance criteria to graduate from stage 1 (beaconed and non-beaconed trials) to stage 2 (beaconed, non-beaconed and probe trials) as a function of mean intensity of GFP fluorescence in layer 2 of the dorsal MEC (left), and proportion of mice that had graduated to stage 2 as a function of training day (right). The graduation day correlated with fluorescence intensity for the TeLC group ($p = 0.00077$, robust least-squares regression), but not the control group ($p = 0.38$; comparison of GFP and TeLC-GFP groups: $p = 0.0012$, for statistical analysis see the Experimental Procedures).

(B) Examples of rasters of stopping locations on day 17 of training for a control mouse, and for mice with high and low expression levels of TeLC (lTeLC and hTeLC). Black dots indicating stopping location are absent on some trials because the animal did not stop.

(C) Mean z-scored probability of stopping as a function of track location during beaconed trials for GFP only control (left), lTeLC (center), and hTeLC mice (right) on days 1–5 and days 15–19.

(D) Comparison of mean z-scored probability of stopping for trained mice (days 15–19) for each group on beaconed trials (left) and probe trials (right).

(E) The difference, between the start of the track and the start of the reward zone, in the probability of stopping ($Stops_{L2-L1}$) (locations L1 and L2 are indicated in Figure 1A) increased with training for GFP mice ($p = 1.4 \times 10^{-10}$, $\chi(1)^2 = 41.2$, likelihood ratio test) and lTeLC mice ($p = 5.2 \times 10^{-8}$, $\chi(1)^2 = 29.6$), but not for hTeLC mice ($p = 0.89$, $\chi(1)^2 = 0.017$).

(F) Analysis of spatial strategy for beaconed trials during days 15-19. The mean location of the first stop (left) differed between control (GFP) and all TeLC mice (lTeLC and hTeLC combined) ($p = 0.021$, percentile bootstrap, test statistic = 16.1, confidence interval [2.9, 26.1]), and hTeLC mice differed from control mice ($p = 0.01$, percentile bootstrap corrected for multiple comparisons, test statistic = 22.2, 95% confidence interval [6.4, 28.6]), but there was no significant difference between lTeLC and control mice ($p = 0.09$, test statistic = 13.1, 95% confidence interval [−0.23, 26.8]). $Stops_{L2-L1}$ (right) differed between control and all TeLC mice (lTeLC and hTeLC combined) ($p = 0.00052$, test statistic = 11.75, 95% confidence interval [3.26, 16.1]), and hTeLC and lTeLC mice differed from control mice (hTeLC: $p = 0.0$, test statistic = 12.1, 95% confidence interval [8.4, 18.1]; lTeLC: $p = 0.034$, test statistic = 5.42, 95% confidence interval [1.1, 14.1]).

(G) Running speed in the black box at the end of the track increased with training for all groups of mice (GFP: $p = 3.5 \times 10^{-11}$, $\chi(1)^2 = 43.7$; lTeLC: $p = 0.0013$, $\chi(1)^2 = 10.4$; hTeLC: $p = 6.5 \times 10^{-6}$, $\chi(1)^2 = 20.3$). During week 4 there was no difference between groups in their running speed within the black box (adjusted $p => 0.7$ for all comparisons, percentile bootstrap test).

(H) Analysis of spatial strategy for probe trials during days 15–19. The first stop location (left) differed between lTeLC and GFP groups ($p = 0.045$, test-statistic = 17.9, 95% confidence interval [0.64, 34.5]). $Stops_{L2-L1}$ during probe trials (right) did not differ significantly between lTeLC and control mice ($p = 0.097$, test statistic = 10.7, 95% confidence interval [−1.2, 12.2]).

Error bars in (A) and (E)–(H) indicate SEM.

location of a reward zone, with the number of available L2SCs determining the rate of learning.

Learning Deficits following the Block of L2SC Output Include Cue- and Path Integration-Based Estimation of Location

How do the deficits in performance of TeLC mice relate to the acquisition of a spatial stopping strategy? We first compared stopping strategies used by mice after >14 days training with stopping strategies used over the first 5 days of training. We found that, after training, control mice and lTeLC mice both demonstrated spatial stopping strategies on beaconed trials (Figures 5B and 5C). The distribution of stop locations was distinct from that of naive mice (Figure 5C), suggesting that mice in both groups learn to stop in the region of the reward zone. In contrast, hTeLC mice did not develop a clear spatial stopping strategy (Figures 5B–5D), and the distribution of stop locations appeared similar to the first week of training (Figures 5C and 5D).

To quantitatively compare these changes, we evaluated the difference between stopping probability at the start of the track and the start of the reward zone, which we refer to as $Stops_{L2\text{-}L1}$, as a function of the day of the experiment (Figure 5E). We found that $Stops_{L2\text{-}L1}$ measured during beaconed trials increased with training for the control group and the lTeLC group, consistent with these mice learning a spatial stopping strategy, but did not change for the hTeLC group (Figure 5E). Comparison of stopping patterns on days 15–19 indicated that mice in the lTeLC group were, nevertheless, impaired relative to the control group, with first stop location and $Stops_{L2\text{-}L1}$ that were intermediate to that of the hTeLC group (Figure 5F). We noticed that training in the task was also associated with an increase in running speed that was particularly apparent in the part of the track between the end of the reward zone and the end of the black box that separates tracks between trials (Figure S1B). To quantify this change, we evaluated the average running speed in the black box as a function of day of the experiment. In contrast to measures of the spatial stopping strategy, running speed in the black box increased with training for control, lTeLC, and hTeLC mice (Figure 5G), suggesting that all mice learn about the structure of the task such that they increase their running speed to minimize the time between consecutive rewards. Consistent with this interpretation, we found no detectable difference between groups in their running speed on days 15–19 (Figure 5G). Thus, inactivation of L2SCs impairs learning of a location, but not task structure, with the size of the deficit dependent on the extent of inactivation.

To evaluate the effects of inactivation of L2SCs on path integration, we compared probe trials between lTeLC and control groups (Figures 5D and 5H). In contrast to control mice, the average distribution of stop locations for lTeLC mice showed little spatial organization on probe trials (Figure 5D). The lTeLC group differed significantly from the EGFP group in the first stop location (Figure 5H), but not the *Z* score difference between the start of the track and the reward zone (Figure 5H). Analysis of preferred stopping locations and running speed also indicated deficits in the lTeLC group compared to the control mice (Figures S6B and S6C). Thus, estimation of location by lTeLC mice on probe trials appears to be impaired.

Together, these results indicate that L2SCs are required to learn a reward location in environments in which beaconing and path integration are the only available strategies, while inactivation of L2SCs does not appear to influence learning about task structure.

L2SC Output Is Required for Object Location Recognition

Finally, to establish whether the deficits we identified in virtual reality-based tests of location estimation extend to real world behaviors, we investigated the effects on location recognition of expressing TeLC in L2SCs. We used an object location memory task that takes advantage of an animal's spontaneous tendency to explore relocated objects (Figure 6A). During a sample phase, mice explored an arena containing two identical objects. During a test phase, one of the objects was relocated to a novel location and mice were allowed to re-explore the arena. To compare the two groups, we calculated the relative time spent exploring each object during the test phase. Whereas control mice that expressed only EGFP in L2SCs showed preferential exploration of the relocated object over the stationary object (discrimination ratio significantly greater than 0, $p = 0.0053$, one-sample t test versus 0, $df = 7$, $t = 3.99$), mice that expressed TeLC in L2SCs did not ($p = 0.45$, one-sample t test versus 0, $df = 7$, $t = 0.79$) (Figure 6B). Consistent with this, discrimination between the objects was substantially lower for mice expressing TeLC in L2SCs compared with control mice (Figure 6B). There was no significant difference in total exploration times between control mice and mice expressing TeLC in L2SCs (Figure 6C), indicating that the differences in discrimination ratios did not reflect disparities in exploration.

To test whether impairments in object location discrimination following the expression of TeLC in L2SCs extend to recognition of objects, we tested the same mice in a version of the task in which a familiar object was replaced with a novel object in the test phase (Figure 6D). Total exploration during the sample and test phases of the task were similar between control and TeLC-expressing mice; both groups showed above-chance discrimination of the novel object, and there was no significant difference in discrimination indexes between control and TeLC-expressing mice, indicating that object recognition was unimpaired (Figures 6E and 6F). Together, these results indicate that, while L2SCs are not required for the recognition of objects, blocking their output impairs discrimination between novel and familiar object locations.

DISCUSSION

By using a virtual reality-based behavioral task, we dissociate estimation of location by beaconing and path integration in conditions in which both visual and motor-related movement information is available. Our results indicate that the accuracy of location estimation by path integration drops steeply as a function of distance, and they suggest that, when visual motion signals are available, mice nevertheless use a motor-based reference frame for path integration. By selectively inhibiting output from L2SCs, we find that this population of medial entorhinal neurons is required for mice to learn to estimate location in the virtual reality task and in a real world object location recognition task. Our data constrain

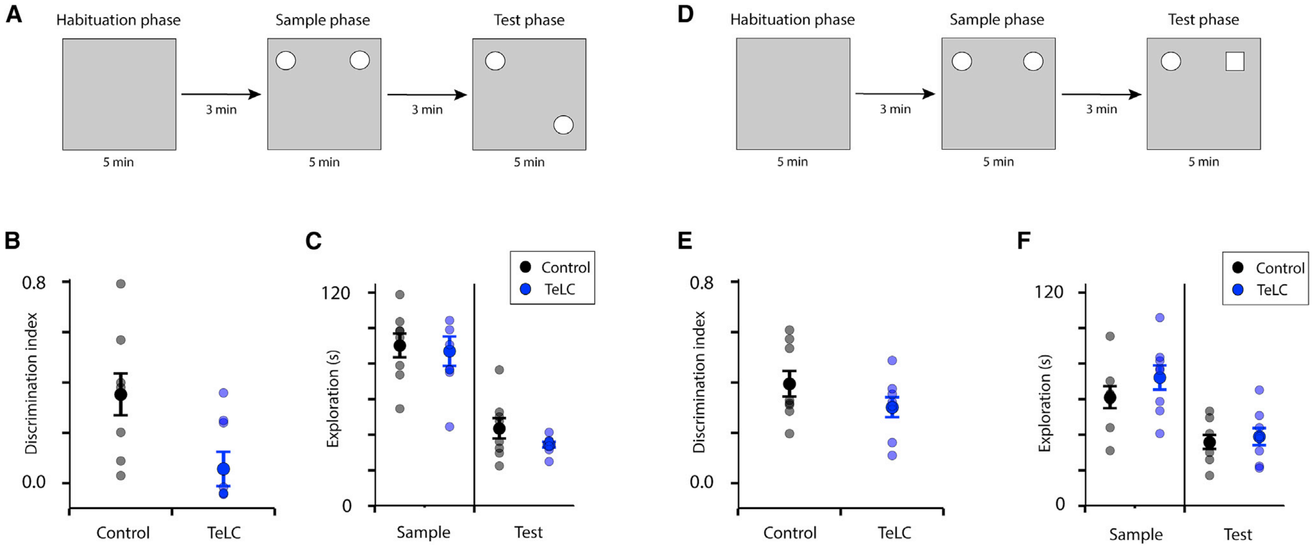

Figure 6. Layer 2 Stellate Cells Are Required for Object Location Recognition

(A and D) Schematized organization of the object location (A) and object recognition (D) experiments. In the test phase of the object location experiment one object is moved to a novel location (A), whereas in the object recognition experiment a novel object is introduced at a familiar location (D).

(B and E) The discrimination index for control mice (Control, N = 8) differed significantly from mice with output from L2SCs inactivated (TeLC, N = 8) in the object location experiment ($p = 0.022$, unpaired t test, $df = 14$, $t = 2.58$) (B), but not the object recognition experiment ($p = 0.19$, unpaired t test, $df = 14$, $t = 1.37$) (E).

(C and F) Total exploration times in the sample and test phases did not differ between animals in the object location experiment (sample phase $p = 0.78$, $df = 14$, $t = 0.28$; test phase $p = 0.17$, $df = 14$, $t = 1.45$) (C) or in the object recognition experiment (sample phase $p = 0.27$, $df = 14$, $t = -1.14$; test phase $p = 0.66$, $df = 14$, $t = -0.45$) (F), indicating that different recognition scores do not result from differences in overall exploration.

Error bars in (B), (C), (E), and (F) indicate SEM.

possible models for behavioral estimation of location, and they provide evidence for a critical involvement of grid cell circuitry.

Because spatial cognition involves parallel perceptual and memory processes, with multiple strategies available to provide the brain with estimates of location, investigation of specific cognitive mechanisms in isolation is challenging. Our approach using a virtual location estimation task is in contrast to real world experiments that isolate path integration from other behavioral strategies through the use of environmental manipulations (Etienne and Jeffery, 2004; Jacob et al., 2017; Van Cauter et al., 2013; Winter et al., 2013). In real world experiments, evaluation of path integration requires the execution of behaviors in darkness in order to prevent confounding influences of visual landmark cues, but this manipulation also removes visual input required for normal function of grid cell circuits, and, therefore, it could impair path integration mechanisms that rely on grid cells (Chen et al., 2016; Pérez-Escobar et al., 2016). Confounding contributions from residual spatial cues, for example, from odors or sounds, are also difficult to fully exclude in real world experiments. In contrast, in the virtual reality-based tasks we introduce here, both visual and motor information is available to the mice, while odor, auditory, or visual cues in the experimental room are not useful in solving the task. Thus, because we were able to represent reward locations in an environment that is devoid of triangulation and beaconing cues, we have been able to specifically probe psychophysical properties of path integration, including dependence on distance and the roles of visual and motor reference frames.

What is the nature of the movement signals used to estimate location by path integration? While place and grid cells can encode elapsed time as well as location (Kraus et al., 2015; Pastalkova et al., 2008), the estimation of time rather than distance is unlikely to explain our observations, as the time taken by an animal to reach the reward zone from the start of the track was a poor predictor of stopping location (Figures S2A and S2B). Recordings from hippocampal place cells suggest that either motor or visual reference frames can be used to represent location (Chen et al., 2013). Because we find that the locations at which mice stop followed the physical distance moved on the treadmill, rather than that predicted by visual signals from the projected track (Figures 2A–2D), our data suggest that behavioral estimation of location by path integration uses a motor-based reference frame. The origin of the motor signals driving the path integrator is unclear, but it may include copies of centrally generated motor commands or proprioceptive feedback. Because mice were head fixed and, therefore, vestibular output is effectively clamped, a necessary role of vestibular motion signals in our experimental conditions can be ruled out. Nevertheless, it is possible that, in different behavioral conditions, the signals used to generate location estimates by path integration may differ.

A critical constraint on behavioral use of path integration to estimate location is the extent to which estimates drift in the absence of spatial cues to anchor the path integrator (Cheung and Vickerstaff, 2010). In models that account for grid cell firing through a path integration mechanism, grid patterns are stable in the absence of noise; but, when noise is introduced into the

neural circuitry, the grid pattern drifts unless additional spatial input is provided (Burak and Fiete, 2009; Guanella et al., 2007; Solanka et al., 2015; Zilli and Hasselmo, 2010). While models differ in their assumptions about the rate at which drift accumulates, in all models additional information about location is required to correct drift. Indeed, as we discuss below, when this additional information is available, for example, from beaconing cues, it may dominate output from circuits capable of path integration. We find that the estimation of location through path integration, but not by beaconing, becomes unreliable for distances >2 m. Since the estimation of longer distances might, in principle, be improved if additional training reduces the sensitivity of the path integrator to noise, our results place only a lower bound on performance. Nevertheless, our observations impose constraints on the behavioral scenarios under which outputs of a neural path integrator may be useful. Our results are also consistent with recent findings that grid fields appear to rapidly drift when visual stimuli are removed (Chen et al., 2016; Pérez-Escobar et al., 2016). While this loss of grid firing may result from an absence of optic flow signals as movement inputs to the grid circuit (Raudies and Hasselmo, 2015; Raudies et al., 2016), our data suggest that distance estimation uses motor signals. Visible spatial cues may instead be critical to anchor grid firing and path integration in the face of drift (Pastoll et al., 2013). The relatively rapid accumulation of drift in grid cell firing (Chen et al., 2016; Pérez-Escobar et al., 2016) and of path integration error that we describe here suggest that, in mice, path integration mechanisms may be important for moment-to-moment tracking of location rather than long-range navigation.

While cells with grid and other spatial firing properties are enriched in superficial layers of the MEC (Sargolini et al., 2006), and previously L2SCs have been shown to be important for contextual learning (Kitamura et al., 2015), it has been unclear whether they contribute to spatial behaviors. Our results provide evidence that L2SCs in the dorsal MEC are required for learning that depends on location estimation within an environment (Figures 5 and 6). These data also speak to a hypothesized role for grid cells as the output of a neural path integrator (McNaughton et al., 2006). Continuous attractor network models that generate grid fields perform path integration using speed and direction signals, but, when external spatial signals are present, they can dictate activity in these circuits. Our results with inactivation of L2SCs corroborate the prediction that, if circuits of this kind are the source of location estimates used to guide behavior, inactivation of these circuits should impair estimation of location by beaconing and by path integration. Nevertheless, additional interpretations are conceivable. Inactivation of external spatial inputs to an integrator circuit would lead to similar behavioral outcomes, although this interpretation is inconsistent with the finding that L2SCs have grid fields (Domnisoru et al., 2013). Alternatively, L2SCs may be downstream of the hypothesized path integrator circuit. In this case, L2SCs must, nevertheless, be a necessary output path by which combined path integration and beaconing signals influence spatial behaviors. The possibility that beaconing and path integration systems operating in parallel, with L2SCs required only for beaconing, appears unlikely, as in this scenario path integration behavior should be maintained after the inactivation of L2SCs.

The effects of our targeted manipulation may be manifest via interactions within L2 of the MEC (Beed et al., 2013; Couey et al., 2013; Pastoll et al., 2013), by projections from L2SCs to cell populations in deeper layers of the MEC (Sürmeli et al., 2015), or through longer range projections to the dentate gyrus and CA3 (Schwartz and Coleman, 1981). Indeed, brain regions downstream of the MEC, including the hippocampus, contain neurons with properties indicative of roles in encoding of path integration and beaconing signals (Chen et al., 2013; Samsonovich and McNaughton, 1997). While following lesions of the MEC place firing in the hippocampus is reduced (Hales et al., 2014) and learning in the water maze is impaired (Hales et al., 2014; Morrissey and Takehara-Nishiuchi, 2014; Steffenach et al., 2005), the loss of place firing is only partial, suggesting that additional spatial signals reach hippocampal structures. One possibility is that L2SCs may be specialized to integrate visual cues with spatial information (Pérez-Escobar et al., 2016; Yoo and Lee, 2017), while spatial information from olfactory or other non-visual cues reaches the hippocampus through the lateral entorhinal cortex (Leitner et al., 2016; Van Cauter et al., 2013). These signals are of no use to solve the virtual reality-based location estimation task, and they may not be sufficient for the object location recognition task under our experimental conditions.

Quantitative dissection of simple behaviors has been essential in establishing underlying computational principles and circuit mechanisms. In contrast, analysis of cognitive behaviors involving multiple sensory modalities is more challenging because of their additional behavioral complexity and because brains may have multiple neural strategies available to solve a task. By implementing a relatively simple spatial task in virtual reality, we have been able to quantitatively dissect roles of beaconing and linear path integration in estimation of location. Our experiments provide evidence to support the long-standing idea that computations by grid cells in the MEC support estimation of location, and, while alternative models remain feasible, our results corroborate key predictions of grid cell models that perform path integration in a manner that integrates external spatial input with velocity signals. Finally, our results may help link deficits in spatial cognition found in dementia to underlying circuit mechanisms. Conceptually similar virtual tasks may be useful as assays of early deficits in dementia, while the key roles we identify for L2SCs suggest that damage restricted to a single cell population at very early stages of degeneration may be detectable by appropriately designed behavioral tests.

EXPERIMENTAL PROCEDURES

Further details and an outline of methods and resources used in this work can be found in the Supplemental Experimental Procedures.

Animals

All animal experiments were carried out under a project license granted by the UK Home Office, were approved by the Animal Welfare and Ethical Review Board (AWERB) of the University of Edinburgh School of Medicine and Veterinary Medicine, and conformed with the UK Animals (Scientific Procedures) Act 1986 and the European Directive 86/609/EEC on the protection of animals used for experimental purposes. Male and female mice, aged 7–12 weeks, were used for all experiments. Mice were randomly allocated to experimental groups.

OPEN ACCESS
CellPress

Data Analysis and Statistical Methods

To quantify virus expression, we measured mean GFP fluorescence using FIJI. Confocal images were opened using the Bio-Formats package (Linkert et al., 2010). Collection, analysis, and presentation of data from virtual reality-based behavioral experiments were performed using custom scripts written in python 3.5 (https://www.python.org) using Numpy version (v.)1.8.1, Scipy v.0.11.0b1, and Matplotlib v.1.5.1 packages. Scripts were written using Spyder 2.3 (www.pythonhosted.org/spyder). Electrophysiology data were analyzed using IGORpro (Wavemetrics). For object exploration tasks, behavior was quantified using the Multitimer scoring system (Vogel-Ciernia and Wood, 2014), and manual scores were confirmed by repeating the scoring using AnyMaze (http://www.anymaze.co.uk/) on video recordings of the mouse's exploration. Full details of quantification are provided in the Supplemental Experimental Procedures.

Statistical analysis was performed in R v.3.30 (R Core Team, 2014). Scripts were written and run using RStudio 0.99.902 (RStudio Team, 2015; https://www.rstudio.com). Details of data distributions and tests are given in the main text and figures. When a measure was obtained repeatedly from the same animal, the mean for that animal was used for population level analyses unless indicated otherwise. Linear mixed effect models (LMEs) were fit using lme 4 1.1-12 (Bates et al., 2015). Animal identity was included in models as a random effect and the variable of interest as a fixed effect. To evaluate significance of effects using LMEs, the model without the variable of interest (a reduced/null model) was compared to the model with the variable of interest using a likelihood ratio test. Because for experiments comparing effects of expression of GFP with TeLC-GFP (Figures 4 and 5) the distribution of the data appeared clearly non-normal, for analysis of these experiments we used robust statistical methods to compare groups (Wilcox, 2016). These were implemented in R using the packages WRS (https://github.com/nicebread/WRS) and WRS2 (v.0.9-2 from https://cran.r-project.org). Comparisons of groups used the percentile bootstrap method. For independent groups, differences between medians were evaluated using the R function medpb2 (in WRS2). For dependent groups, the bootdpci function (in WRS) was used to compare 20% trimmed means. Results are reported using 10^5 bootstrap samples. Linear regression was performed using a least-squares method that allows heteroscedasticity, implemented in the R function olshc4 (WRS package), with slopes compared using the R function ols2ci (WRS package). For multiple comparisons within an experiment, reported p values were adjusted by the Benjamini and Hochberg method using the R function p.adjust.

Data and code to reproduce the analyses reported in the paper will be made available via the University of Edinburgh DataShare repository (http://dx.doi.org/10.7488/ds/2290). Analysis code will be made available via the Nolan Lab GitHub repository (https://github.com/MattNolanLab).

SUPPLEMENTAL INFORMATION

Supplemental Information includes Supplemental Experimental Procedures, six figures, and one movie and can be found with this article online at https://doi.org/10.1016/j.celrep.2018.01.005.

ACKNOWLEDGMENTS

We thank Ian Duguid for advice in establishing head fixation and treadmill technologies, Gülşen Sürmeli for assistance in targeting of L2SCs, and Jessica Menzies and Michelle Haglund for collecting the object recognition and object location data. This work was supported by the BBSRC (BB/L010496/1 and a BBSRC Eastbio studentship [grant BB/M010996/1]), the Human Frontiers Science Program (RGP0062/2014), and the Wellcome Trust (200855/Z/16/Z and WT093295MA). The funders had no role in study design, data collection and analysis, decision to publish, or preparation of the manuscript.

AUTHOR CONTRIBUTIONS

Conceptualization, S.A.T., L.F., and M.F.N.; Methodology, S.A.T., L.F., E.R.W., and M.F.N.; Software, S.A.T., K.Z.G., and L.F.; Formal Analysis, S.A.T., D.L.F.G., C.M.-G., and M.F.N.; Investigation, S.A.T., C.M.-G., and D.L.F.G.; Resources, C.M.; Writing – Original Draft, M.F.N. and S.A.T.; Writing – Review & Editing, M.F.N., E.R.W., and S.A.T.; Supervision, M.F.N.; Project Administration, M.F.N.; Funding Acquisition, M.F.N.

DECLARATION OF INTERESTS

The authors declare no competing interests.

Received: August 7, 2017
Revised: December 5, 2017
Accepted: January 2, 2018
Published: January 30, 2018

REFERENCES

Bates, D., Machler, M., Bolker, B.M., and Walker, S.C. (2015). Fitting linear mixed-effects models using lme4. J. Stat. Softw. *67*, 1–48.

Beed, P., Gundlfinger, A., Schneiderbauer, S., Song, J., Böhm, C., Burgalossi, A., Brecht, M., Vida, I., and Schmitz, D. (2013). Inhibitory gradient along the dorsoventral axis in the medial entorhinal cortex. Neuron *79*, 1197–1207.

Burak, Y., and Fiete, I.R. (2009). Accurate path integration in continuous attractor network models of grid cells. PLoS Comput. Biol. *5*, e1000291.

Burgess, N., and O'Keefe, J. (2011). Models of place and grid cell firing and theta rhythmicity. Curr. Opin. Neurobiol. *21*, 734–744.

Bush, D., Barry, C., Manson, D., and Burgess, N. (2015). Using grid cells for navigation. Neuron *87*, 507–520.

Chen, G., King, J.A., Burgess, N., and O'Keefe, J. (2013). How vision and movement combine in the hippocampal place code. Proc. Natl. Acad. Sci. USA *110*, 378–383.

Chen, G., Manson, D., Cacucci, F., and Wills, T.J. (2016). Absence of Visual Input Results in the Disruption of Grid Cell Firing in the Mouse. Curr. Biol. *26*, 2335–2342.

Cheung, A. (2016). Probabilistic Learning by Rodent Grid Cells. PLoS Comput. Biol. *12*, e1005165.

Cheung, A., and Vickerstaff, R. (2010). Finding the way with a noisy brain. PLoS Comput. Biol. *6*, e1000992.

Collett, M., Collett, T.S., Bisch, S., and Wehner, R. (1998). Local and global vectors in desert ant navigation. Nature *394*, 269–272.

Collett, M., Chittka, L., and Collett, T.S. (2013). Spatial memory in insect navigation. Curr. Biol. *23*, R789–R800.

Couey, J.J., Witoelar, A., Zhang, S.J., Zheng, K., Ye, J., Dunn, B., Czajkowski, R., Moser, M.B., Moser, E.I., Roudi, Y., and Witter, M.P. (2013). Recurrent inhibitory circuitry as a mechanism for grid formation. Nat. Neurosci. *16*, 318–324.

Diehl, G.W., Hon, O.J., Leutgeb, S., and Leutgeb, J.K. (2017). Grid and nongrid cells in medial entorhinal cortex represent spatial location and environmental features with complementary coding schemes. Neuron *94*, 83–92.e6.

Domnisoru, C., Kinkhabwala, A.A., and Tank, D.W. (2013). Membrane potential dynamics of grid cells. Nature *495*, 199–204.

Durrant-Whyte, H., and Bailey, T. (2006). Simultaneous localization and mapping: part I. IEEE Robot. Autom. Mag. *13*, 99–110.

Etienne, A.S., and Jeffery, K.J. (2004). Path integration in mammals. Hippocampus *14*, 180–192.

Etienne, A.S., Maurer, R., and Séguinot, V. (1996). Path integration in mammals and its interaction with visual landmarks. J. Exp. Biol. *199*, 201–209.

Fuchs, E.C., Neitz, A., Pinna, R., Melzer, S., Caputi, A., and Monyer, H. (2016). Local and distant input controlling excitation in layer ii of the medial entorhinal cortex. Neuron *89*, 194–208.

Fuhs, M.C., and Touretzky, D.S. (2006). A spin glass model of path integration in rat medial entorhinal cortex. J. Neurosci. *26*, 4266–4276.

Geva-Sagiv, M., Las, L., Yovel, Y., and Ulanovsky, N. (2015). Spatial cognition in bats and rats: from sensory acquisition to multiscale maps and navigation. Nat. Rev. Neurosci. *16*, 94–108.

Giocomo, L.M., Moser, M.B., and Moser, E.I. (2011). Computational models of grid cells. Neuron *71*, 589–603.

Guanella, A., Kiper, D., and Verschure, P. (2007). A model of grid cells based on a twisted torus topology. Int. J. Neural Syst. *17*, 231–240.

Hafting, T., Fyhn, M., Molden, S., Moser, M.B., and Moser, E.I. (2005). Microstructure of a spatial map in the entorhinal cortex. Nature *436*, 801–806.

Hales, J.B., Schlesiger, M.I., Leutgeb, J.K., Squire, L.R., Leutgeb, S., and Clark, R.E. (2014). Medial entorhinal cortex lesions only partially disrupt hippocampal place cells and hippocampus-dependent place memory. Cell Rep. *9*, 893–901.

Hardcastle, K., Maheswaranathan, N., Ganguli, S., and Giocomo, L.M. (2017). A multiplexed, heterogeneous, and adaptive code for navigation in medial entorhinal cortex. Neuron *94*, 375–387.e7.

Jacob, P.Y., Gordillo-Salas, M., Facchini, J., Poucet, B., Save, E., and Sargolini, F. (2017). Medial entorhinal cortex and medial septum contribute to self-motion-based linear distance estimation. Brain Struct. Funct. *222*, 2727–2742.

Kitamura, T., Sun, C., Martin, J., Kitch, L.J., Schnitzer, M.J., and Tonegawa, S. (2015). Entorhinal cortical ocean cells encode specific contexts and drive context-specific fear memory. Neuron *87*, 1317–1331.

Kraus, B.J., Brandon, M.P., Robinson, R.J., 2nd, Connerney, M.A., Hasselmo, M.E., and Eichenbaum, H. (2015). During running in place, grid cells integrate elapsed time and distance run. Neuron *88*, 578–589.

Kropff, E., and Treves, A. (2008). The emergence of grid cells: Intelligent design or just adaptation? Hippocampus *18*, 1256–1269.

Leitner, F.C., Melzer, S., Lütcke, H., Pinna, R., Seeburg, P.H., Helmchen, F., and Monyer, H. (2016). Spatially segregated feedforward and feedback neurons support differential odor processing in the lateral entorhinal cortex. Nat. Neurosci. *19*, 935–944.

Linkert, M., Rueden, C.T., Allan, C., Burel, J.M., Moore, W., Patterson, A., Loranger, B., Moore, J., Neves, C., Macdonald, D., et al. (2010). Metadata matters: access to image data in the real world. J. Cell Biol. *189*, 777–782.

Mathis, A., Herz, A.V., and Stemmler, M. (2012). Optimal population codes for space: grid cells outperform place cells. Neural Comput. *24*, 2280–2317.

McNaughton, B.L., Barnes, C.A., Gerrard, J.L., Gothard, K., Jung, M.W., Knierim, J.J., Kudrimoti, H., Qin, Y., Skaggs, W.E., Suster, M., and Weaver, K.L. (1996). Deciphering the hippocampal polyglot: the hippocampus as a path integration system. J. Exp. Biol. *199*, 173–185.

McNaughton, B.L., Battaglia, F.P., Jensen, O., Moser, E.I., and Moser, M.B. (2006). Path integration and the neural basis of the 'cognitive map'. Nat. Rev. Neurosci. *7*, 663–678.

Milford, M.J., Wiles, J., and Wyeth, G.F. (2010). Solving navigational uncertainty using grid cells on robots. PLoS Comput. Biol. *6*, e1000995.

Morrissey, M.D., and Takehara-Nishiuchi, K. (2014). Diversity of mnemonic function within the entorhinal cortex: a meta-analysis of rodent behavioral studies. Neurobiol. Learn. Mem. *115*, 95–107.

Murray, A.J., Sauer, J.F., Riedel, G., McClure, C., Ansel, L., Cheyne, L., Bartos, M., Wisden, W., and Wulff, P. (2011). Parvalbumin-positive CA1 interneurons are required for spatial working but not for reference memory. Nat. Neurosci. *14*, 297–299.

Pastalkova, E., Itskov, V., Amarasingham, A., and Buzsáki, G. (2008). Internally generated cell assembly sequences in the rat hippocampus. Science *321*, 1322–1327.

Pastoll, H., Solanka, L., van Rossum, M.C., and Nolan, M.F. (2013). Feedback inhibition enables θ-nested γ oscillations and grid firing fields. Neuron *77*, 141–154.

Pérez-Escobar, J.A., Kornienko, O., Latuske, P., Kohler, L., and Allen, K. (2016). Visual landmarks sharpen grid cell metric and confer context specificity to neurons of the medial entorhinal cortex. eLife *5*, e16937.

Raudies, F., and Hasselmo, M.E. (2015). Differences in visual-spatial input may underlie different compression properties of firing fields for grid cell modules in medial entorhinal cortex. PLoS Comput. Biol. *11*, e1004596.

Raudies, F., Hinman, J.R., and Hasselmo, M.E. (2016). Modelling effects on grid cells of sensory input during self-motion. J. Physiol. *594*, 6513–6526.

Samsonovich, A., and McNaughton, B.L. (1997). Path integration and cognitive mapping in a continuous attractor neural network model. J. Neurosci. *17*, 5900–5920.

Sargolini, F., Fyhn, M., Hafting, T., McNaughton, B.L., Witter, M.P., Moser, M.B., and Moser, E.I. (2006). Conjunctive representation of position, direction, and velocity in entorhinal cortex. Science *312*, 758–762.

Schmidt-Hieber, C., and Häusser, M. (2013). Cellular mechanisms of spatial navigation in the medial entorhinal cortex. Nat. Neurosci. *16*, 325–331.

Schwartz, S.P., and Coleman, P.D. (1981). Neurons of origin of the perforant path. Exp. Neurol. *74*, 305–312.

Solanka, L., van Rossum, M.C., and Nolan, M.F. (2015). Noise promotes independent control of gamma oscillations and grid firing within recurrent attractor networks. eLife *4*, e06444.

Solstad, T., Boccara, C.N., Kropff, E., Moser, M.B., and Moser, E.I. (2008). Representation of geometric borders in the entorhinal cortex. Science *322*, 1865–1868.

Sreenivasan, S., and Fiete, I. (2011). Grid cells generate an analog error-correcting code for singularly precise neural computation. Nat. Neurosci. *14*, 1330–1337.

Steffenach, H.A., Witter, M., Moser, M.B., and Moser, E.I. (2005). Spatial memory in the rat requires the dorsolateral band of the entorhinal cortex. Neuron *45*, 301–313.

Stemmler, M., Mathis, A., and Herz, A.V. (2015). Connecting multiple spatial scales to decode the population activity of grid cells. Sci. Adv. *1*, e1500816.

Sun, C., Kitamura, T., Yamamoto, J., Martin, J., Pignatelli, M., Kitch, L.J., Schnitzer, M.J., and Tonegawa, S. (2015). Distinct speed dependence of entorhinal island and ocean cells, including respective grid cells. Proc. Natl. Acad. Sci. USA *112*, 9466–9471.

Sürmeli, G., Marcu, D.-C., McClure, C., Garden, D.L.F., Pastoll, H., and Nolan, M.F. (2015). Molecularly defined circuitry reveals input-output segregation in deep layers of the medial entorhinal cortex. Neuron *88*, 1040–1053.

Van Cauter, T., Camon, J., Alvernhe, A., Elduayen, C., Sargolini, F., and Save, E. (2013). Distinct roles of medial and lateral entorhinal cortex in spatial cognition. Cereb. Cortex *23*, 451–459.

Varga, C., Lee, S.Y., and Soltesz, I. (2010). Target-selective GABAergic control of entorhinal cortex output. Nat. Neurosci. *13*, 822–824.

Vogel-Ciernia, A., and Wood, M.A. (2014). Examining object location and object recognition memory in mice. Curr. Protoc. Neurosci. *69*, 8.31.1–8.31.17.

Wilcox, R.R. (2016). Introduction to Robust Estimation and Hypothesis Testing, Fourth Edition (Waltham, MA: Elsevier).

Winter, S.S., Köppen, J.R., Ebert, T.B., and Wallace, D.G. (2013). Limbic system structures differentially contribute to exploratory trip organization of the rat. Hippocampus *23*, 139–152.

Wittlinger, M., Wehner, R., and Wolf, H. (2006). The ant odometer: stepping on stilts and stumps. Science *312*, 1965–1967.

Yoo, S.W., and Lee, I. (2017). Functional double dissociation within the entorhinal cortex for visual scene-dependent choice behavior. eLife *6*, e21543.

Zilli, E.A. (2012). Models of grid cell spatial firing published 2005-2011. Front. Neural Circuits *6*, 16.

Zilli, E.A., and Hasselmo, M.E. (2010). Coupled noisy spiking neurons as velocity-controlled oscillators in a model of grid cell spatial firing. J. Neurosci. *30*, 13850–13860.

Cell Reports, Volume *22*

Supplemental Information

Stellate Cells in the Medial Entorhinal Cortex

Are Required for Spatial Learning

Sarah A. Tennant, Lukas Fischer, Derek L.F. Garden, Klára Zsófia Gerlei, Cristina Martinez-Gonzalez, Christina McClure, Emma R. Wood, and Matthew F. Nolan

Supplemental Experimental Procedures

CONTACT FOR REAGENT AND RESOURCE SHARING

Matthew Nolan (mattnolan@ed.ac.uk) is the Lead Contact for reagent and resource sharing. All published reagents will be shared on an unrestricted basis; reagent requests should be directed to the lead author.

RESOURCES TABLE

REAGENT or RESOURCE	SOURCE	IDENTIFIER
Antibodies		
NeuroTraceÂ® 640/660 Deep-Red Fluorescent Nissl Stain - Solution in DMSO	Thermo Fisher Scientific Inc.	Cat# N21483
NeuroTraceÂ® 530/610 Red Fluorescent Nissl Stain - Solution in DMSO	Thermo Fisher Scientific Inc.	Cat# N21482
Rabbit anti Calbindin D-28k	Swant	Cat# CB 38
Mouse Anti-Reelin Antibody	Millipore	Cat# MAB5364
Goat anti-Rabbit IgG (H+L) Secondary Antibody, Alexa FluorÂ® 405 conjugate	Invitrogen	Cat# A-31556
Goat anti-Mouse IgG (H+L) Secondary Antibody, Alexa FluorÂ® 647 conjugate	Invitrogen	Cat# A-21236
Bacterial and Virus Strains		
AAV1/2-FLEX-TeLC-GFP	Murray et al., 2011	NA
AAV1/2-FLEX-GFP	Murray et al., 2011	NA
AAV1/2-FLEX-rev-ChR2(H134R)-mCherry	Atasoy et al., 2008	Plasmid from Addgene Cat#18916
Chemicals, Peptides, and Recombinant Proteins		
LOCTITE 3 g Transparent Gel Tube Cyanoacrylate Adhesive for Various Materials Ultra Gel	RS components	Cat# 330-4018
Paraformaldehyde powder 95%	Sigma Aldrich	Cat# 158127
Phosphate Buffer Powder	Sigma Aldrich	Cat# P7994-1EA
Simplex Powder Pink Shade S28/1	Associated Dental Products Ltd	Cat# ACR806
Simplex Rapid Liquid	Associated Dental Products Ltd	Cat# ACR924
Deposited Data		
Raw and analyzed data	This Paper	http://datashare.is.ed.ac.uk/

Analysis code	This Paper	https://github.com/orgs/MattNolanLab/
Body weight information for C57BL/6J (000664)	Jax Jaboratory	https://www.jax.org/jax-mice-and-services/strain-data-sheet-pages/body-weight-chart-000664
Experimental Models: Organisms/Strains		
Mouse: Tg(Sim1cre)KJ21Gsat/ Mmucd	MMRRC	RRID:MMRRC_034614-UCD
Mouse: JAX C57/BL6jCrl	Charles River	Cat# 000664
Software and Algorithms		
Any – Maze Behavioural Tracking Software	Stoelting Co.	http://www.anymaze.co.uk
Blender3D	Bourke and Felinto (2010)	https://www.blender.org
Fiji is just ImageJ	Schindelin et al (2012)	https://fiji.sc
Bio-Formats	Linkert et al (2010)	https://www.openmicroscopy.org/bio-formats/
Multitimer	Patrick Spooner, University of Edinburgh	NA
Python programming language v3.6.1	Van Rossum (1995)	Python Software Foundation (https://www.python.org)
R v3.4.2	RCore Team (2013)	https://www.r-project.org
RStudio v1.0.153	RStudio Team (2016)	http://www.rstudio.com/
Spyder - The Scientific PYthon Development EnviRonment v2.3.5	Github repository	www.pythonhosted.org/spyder / https://github.com/spyder-ide/spyder
Numpy v1.8.1	van der Walt et a (2011)	http://www.numpy.org
Scipy v0.11.0b1	Jones et al (2001)	https://www.scipy.org
Matplotlib v1.5.1	Hunter (2007)	https://matplotlib.org

ggplot2 v2.2.1	Wickham (2009)	http://ggplot2.org
gridExtra	Auguie (2017)	https://cran.r-project.org/web/packages/gridExtra/index.html
dplyr v0.7.4	Wickham et al (2017)	https://cran.r-project.org/web/packages/dplyr/index.html
tidyr	Wickham and Henry (2017)	https://cran.r-project.org/web/packages/tidyr/index.html
lme4 v1.1-14	Cran repository	https://cran.r-project.org/
Nlme v3.1-131	Cran repository / Pinheiro et al (2017)	https://cran.r-project.org/web/packages/nlme/index.html
WRS2 v0.3-2	Cran repository	https://cran.r-project.org/
WRS	R forge repository	http://R-Forge.R-project.org
Key components of virtual reality set up		
Cylindrical polystyrene treadmill	Graham Sweet Studios	Custom made *
Silicon tubing	NResearch	Cat# TBGM101
Torus shaped screen	Talbot Designs Limited	Custom made *
Rotary encoder	Pewatron	Cat# E6-2500-472-IE
Disposable Straight Feeding Needle, 18 Gauge, 50.8 mm	Harvard apparatus	Cat# 598636
1Tube Normally Closed Full Opening Pinch Valve	NResearch	Cat# 225PNC1-21
Arduino Due	RS Components	Cat# 769-7412
L-shaped head post	3D Creations Lab	Custom made *
Feeding needle holder	3D Creations Lab	Custom made *
CoolDrive one single valve driver	NResearch	Cat# 225D1X180
Head clamp	Thor Labs	Custom made *
Enhanced aluminium coated mirror, 140mm diameter x 6mm thick	Knight Optical Ltd	Custom made *
Angular amplification mirror	3D Creations lab	Custom made *
Other		
Alpro Dairy Free Fresh Milk Alternative Soya, Original	Sainsbury's	NA

* Custom designs will be shared upon request

REFERENCES

Atasoy D, Aponte Y, Su HH, Sternson SM. *J.* A FLEX switch targets Channelrhodopsin-2 to multiple cell types for imaging and long-range circuit mapping. *Neurosci. 2008 Jul 9. 28(28):7025-30.* 10.1523/JNEUROSCI.1954-08.2008PubMed 18614669

Baptiste Auguie (2017). gridExtra: Miscellaneous Functions for "Grid" Graphics. R package version 2.3. https://CRAN.R-project.org/package=gridExtra

Bourke, P. D. and Felinto, D. Q. (2010), Blender and Immersive Gaming in a Hemispherical Dome , Global Science & Technology Forum (GSTF).

G. van Rossum, Python tutorial, Technical Report CS-R9526, Centrum voor Wiskunde en Informatica (CWI), Amsterdam, May 1995

John D. Hunter. Matplotlib: A 2D Graphics Environment, Computing in Science & Engineering, 9, 90-95 (2007),DOI:10.1109/MCSE.2007.55 (publisher link)

Jones E, Oliphant E, Peterson P, *et al.* SciPy: Open Source Scientific Tools for Python**,** 2001- , http://www.scipy.org/ [Online; accessed 2017-12-19].

Linkert, M., Rueden, C. T., Allan, C., Burel, J. M., Moore, W., Patterson, A., Loranger, B., Moore, J., Neves, C., MacDonald, D., Tarkowska, A., Sticco, C., Hill, E., Rossner, M., Eliceiri, K. W. and Swedlow, J. R. (2010), ‘Metadata matters: Access to image data in the real world’, Journal of Cell Biology 189 (5), 777–782.

Murray AJ[1], Sauer JF, Riedel G, McClure C, Ansel L, Cheyne L, Bartos M, Wisden W, Wulff P. Parvalbumin-positive CA1 interneurons are required for spatial working but not for reference memory. Nat Neurosci. 2011 Mar;14(3):297-9. doi: 10.1038/nn.2751. Epub 2011 Jan 30.

Pinheiro J, Bates D, DebRoy S, Sarkar D and R Core Team (2017). _nlme: Linear and Nonlinear Mixed Effects Models_. R package version 3.1-131, <URL: https://CRAN.R-project.org/package=nlme>.

R Core Team (2013). R: A language and environment for statistical computing. R Foundation for Statistical Computing, Vienna, Austria. URL http://www.R-project.org/

RStudio Team (2016). RStudio: Integrated Development for R. RStudio, Inc., Boston, MA URL http://www.rstudio.com/.

Schindelin, J., Arganda-Carreras, I., Frise, E., Kaynig, V., Longair, M., Pietzsch, T., Preibisch, S., Rueden, C., Saalfeld, S., Schmid, B., Tinevez, J.-Y., White, D. J., Hartenstein, V., Eliceiri, K., Tomancak, P. and Cardona, A. (2012), 'Fiji: an open-source platform for biological-image analysis', Nature Methods 9 (7), 676–682.

Stéfan van der Walt, S. Chris Colbert and Gaël Varoquaux. The NumPy Array: A Structure for Efficient Numerical Computation, Computing in Science & Engineering, 13, 22-30 (2011), DOI:10.1109/MCSE.2011.37 (publisher link)

H. Wickham. ggplot2: Elegant Graphics for Data Analysis. Springer-Verlag, New York, 2009.

H. Wickham, Romain Francois, Lionel Henry and Kirill Müller (2017). dplyr: A Grammar of Data Manipulation. R package version 0.7.4. https://CRAN.R-project.org/package=dplyr

H. Wickham and L. Henry (2017). tidyr: Easily Tidy Data with 'spread()' and 'gather()' Functions. R package version 0.7.2. https://CRAN.R-project.org/package=tidyr

EXPERIMENTAL MODEL AND SUBJECT DETAILS

All animal experiments were carried out in compliance with protocols approved by the Animal Welfare and Ethical Review Board (AWERB) of the University of Edinburgh School of Medicine and Veterinary Medicine and under a UK Home Office Project License. Male and female mice, aged 7 – 12 weeks, were used for all experiments. Mice were randomly allocated to experimental groups. To selectively manipulate the activity of stellate cells in layer 2 of the MEC, a transgenic mouse line ($Sim1^{Cre}$) was used that enabled genetic access to layer 2 stellate cells as described in Sürmeli et al (2015). In this mouse line, Cre expression is controlled by the Single minded homolog-1 (Sim1) promoter. The line was generated by GenSat and obtained from MMRRC (strain name: Tg(Sim1cre)KJ21Gsat/ Mmucd). It was maintained by crossing male $Sim1^{Cre}$ mice with female C57/BL6jCrl wild-type mice (Charles River). Animals were kept in standard cages containing toys and wheels, and were maintained on a 12:12 hour reverse light:dark schedule (with lights off at 7am). Animals were group housed (up to 4 mice per cage post weaning) until surgeries for head post implantation and then were single housed. Mice had free access to food and water until the beginning of food deprivation described below. All animals had access to a running wheel in their home cages, except for two days of postoperative recovery. Small cardboard tubes were also placed in each cage after postoperative recovery for environmental enrichment.

METHOD DETAILS

Virtual-reality system. Mice were attached to a custom designed head clamp (Thor Labs) situated above a cylindrical polystyrene treadmill (Graham Sweet Studios) that allows movement in two directions; forward and back. Linear virtual tracks designed in Blender3D (Blender.com) were projected onto a torus shaped screen (Talbot Designs Limited) that surrounded the animal. The system was designed so that the convex mirror spreading the image over the screen has a

surface profile that creates a linear relationship between the incident angle and the deflection angle across its entire surface. The animal is placed at one intersection point of the torus, while the convex mirror is placed at the other intersection point, thus creating a horopter. The resulting linear relationships along the horizontal and vertical axes between the projection of the virtual environment, and its perception in terms of movement across the animal's retina, avoid 'warping' or other unnatural movement of visual flow patterns across the animal's field of vision. A rotary encoder (Pewatron, E6-2500-472-IE) that monitors movement on the treadmill communicates with Blender3D, which was used to generate and update the virtual environment. A feeding tube (Harvard Apparatus) placed in front of the animal, and controlled by a normally closed, full opening pinch valve (NResearch, 225PNC1-21), was used to dispense soy-milk rewards (5-10 μl per reward). Stops in the reward zone for a pre-specified duration were detected by Blender3D, which interacted with the pinch valve via a microcontroller (Arduino Due, www.arduino.cc).

Virtual-track design. The standard virtual track was 2 m long with a 20 cm wide floor and 20 cm high walls. The floor had a wooden pattern and the proximal walls were decorated with a uniform repeating pattern of pseudo-randomized black and white dots. To allow consecutive trials to be clearly distinguished, the visible track was preceded and followed by black boxes of length 30 cm, such that locations -30 cm to 0 cm correspond to a black box, locations 0 to 140 cm to the visible track and locations 140 to 170 cm to a black box. On reaching the end of the track animals were 'teleported' back to the beginning to start another trial. To prevent the black box being used as a distal cue for locating the reward zone the visible the track was restricted to 50 cm ahead of the animal.

For beaconed trials a reward zone was located 60-80 cm from the start of the visible track. The reward zone was marked by cues consisting of black and green vertical stripes on the track walls. For non-beaconed and probe trials the cues were removed. On beaconed and non-beaconed trials, but not on probe trials, rewards were dispensed when mice stopped in the reward zone.

During standard trials every 1 cm travelled on the treadmill moves the animal an equivalent virtual unit (VU) along the visually projected track (1 cm: 1 VU). For gain modulation trials the conversion of physical movement on the treadmill to virtual movement was altered such that, the ratio of distance moved on the treadmill to distance traversed in the virtual linear track was either halved (gain x 0.5, 2 cm: 1 VU) or doubled (gain x 2, 1 cm: 2 VU).

For experiments in which the track length was increased, a series of tracks were generated (track 1 to track n) in which the distance from the end of the first black box to the start of the reward zone was described by the equation, $L_n = L_{n-1}*1.5$, where L_n and L_{n-1} are the distance from the start point to the reward zone for the current and previous tracks in the series. Track 1 was the standard track described above (length = 2m, L = 60 cm and gain = 1). The black boxes at the start and end of the tracks were 30 cm long for all tracks.

Head fixation. A head-post was attached to the skull of each mouse enabling it to be head fixed in the virtual reality set up. Surgeries were performed 10-13 days before beginning behavioral experiments. Mice were anesthetized using 1-3 % Isoflurane gas (Abbott Laboratories, IL) in oxygen. Analgesia was achieved by subcutaneous administration of Vetergesic (Ceva, UK) at a dose of 0.08 ml/kg bodyweight. The scalp was shaved and cleaned with antiseptic, the mouse was placed in a stereotaxic frame (Stoelting 51900) and maintained at 37°C using a

thermostatic heat blanket. The eyes were covered throughout surgery with hydrating opaque eye-gel (Viscotears, TX). A small region of the scalp (approx. 1.8 mm by 2.5 mm) was removed to expose lambda and bregma, the top of the skull was cleaned with isotonic saline and connective tissue removed with a scalpel blade. The skin was then glued to the skull with Vetbond (VetTech) leaving the top of the skull exposed for head-post implantation. A scalpel blade was used to cut ridges into the top of the skull. An L-shaped head post (Protolabs) was lowered onto the skull and fixed in place with Loctite glue. The exposed skull and post were then embedded in dental cement (Simplex rapid acrylic denture polymer, Associated Dental Products Ltd, UK). Following surgery mice were then placed on a heat bench for 30 - 60 minutes until they regained consciousness, and were given additional analgesic (Vetergesic) at 8-12 hours post surgery.

Viral injections. Adeno-associated viruses (AAVs) viruses were targeted stereotaxically to the MEC as described previously (Sürmeli et al., 2015). For behavioral experiments, viral injections were carried out during the head post surgery. Injections were via bilateral craniotomies targeted at 3.5 mm from bregma along the medial lateral axis and just above the transverse sinus along the anterior posterior axis. For each craniotomy, four injections were made along the dorsal-ventral axis of the MEC at intervals of approximately 200 μm. All four injections were performed using the same injection coordinates, but differed in the angle of the micropipette and depth of the injection site. For the first injection, which aimed to hit the dorsal most portion of the MEC, the micropipette was lowered at an angle of -16°. For each injection after this the angle was incrementally reduced by 3° so that the last injection was performed at an angle of -7° for targeting the ventral portion of layer 2. The depth of the injection sites was determined form the depth at which the micropipette bends once it hit the dura lining the surface of the brain. Approximate depths at which a bend should be seen in an 8 week old mouse are as follows for

each injection site: 1st injection: 1.8 mm; 2nd injection: 2 mm; 3rd injection: 2.2 mm; 4th injection: 2.4 mm.

Viruses were generated in-house and were titered by qPCR following expression in HEK cells. Viruses used where AAV-TeLC-GFP and AAV-GFP (obtained from Peer Wulf, Kiel University)(Murray et al., 2011), and AAV-FLEX-rev-ChR2(H134R)-mCherry (Addgene plasmid #18916). AAV-TeLC-GFP had a titer of 6.4 x 10^{12} cp/ml, AAV-GFP had a titer of 5.1 x 10^{12} cp/ml and AAV-FLEX-rev-ChR2(H134R)-mCherry had a titer of 9.45 x 10^{12} cp/ml. We injected 400-650 nl of AAV at each site. AAVs were generated with a chimeric 1/2 serotype as described previously (McClure et al., 2011).

Handling and habituation of experimental mice. To minimize behavioral variability the daily routine of the experimenter was made as consistent as possible (e.g. the same products were used for bodily hygiene, hands were cleaned before training in the same way etc). For one week after surgical procedures mice were handled daily for ten minutes, once in the morning and once in the afternoon for 5 and 10 minutes respectively. During each handling session mice were weighed to establish a baseline body weight for subsequent food deprivation.

Habituation to the apparatus began a minimum of a week after post-operative recovery. Mice were habituated to the head restraint on two consecutive days for sessions lasting 5 and 10 minutes respectively. Habituation sessions for each mouse were carried out at the same time of day as their subsequent training. During habituation sessions the virtual reality was on, but rewards were not dispensed. To minimize stress experienced by the mice, they were encouraged to walk voluntarily onto the treadmill before head restraint. Mice were handled for a further 5 minutes after habituation sessions.

Food deprivation. Mice began food deprivation after the final habituation session. Initially, mice were deprived of food for 24 hours. They were then fed 3.5 g of food daily at their allocated training time for 4 days before starting behavioral training. During behavioral training mice were fed between 3 and 4 g of chow 30 minutes after the end of their training session. The amount of food depended on their behavior during the training session and their current weight relative to their reference body weight. A daily growth curve was adjusted for each mouse according to its pre-experiment body weight (see (https://www.jax.org/jax-mice-and-services/strain-data-sheet-pages/body-weight-chart-000664)). The mouse's weight was then calculated as a percentage of the reference body weight and daily food was allotted to achieve a target bodyweight of 85 % of the reference body weight.

During behavioral training mice were trained in 5 day blocks followed by a 2 day break. For feeding on break days a specific protocol was followed. On the last training session of each block mice were fed 1 g more than their daily feeding amount. On the first day of the break mice were fed 0.5 g more than their daily feeding amount and on the last day of the break mice resumed their usual feeding amount.

Task rewards, criteria and stage progression. During behavioral experiments, mice were rewarded with 5 - 10 μl soy-milk when they stopped for a specified duration in the reward zone. A stop is detected during the experiment when the running speed of the mouse (calculated as the distance covered over the last 200 ms) drops below 0.7 cm/sec. On beaconed and non-beaconed trials mice were rewarded the first time they stopped in the reward zone. At the same time as the reward was released a 500 ms tone was played to reinforce the place-reward association. Rewards were not given on probe trials.

Each behavioral experiment was split into distinct stages. In stage 1, four out of five trials used beaconed tracks, and one out of five used non-beaconed tracks. In stage 2, probe trials replaced one out of every two trials on non-beaconed tracks. Mice progressed from stage 1 to stage 2 after a minimum number of days and when 75 % of trials in a session were successfully rewarded over two consecutive training days. For the experiment described in Figure 1, stage 1 was split into two sub-stages. In the first, the delay between stopping in the reward zone and receiving a reward was 0 ms. The delay was increased to 125 ms when mice completed 30 or more trials with ≥ 75 % of trials rewarded over two consecutive days. Once mice performed ≥ 75 % on a 125 ms delay, they graduated to stage 2 of the behavioral experiment. In this experiment, the minimum number of days to progress from stage 1 to stage 2 was 15. Following this set of experiments we decided the additional 125 ms delay was unnecessary given that probe trials could be used to assay performance and so in all other experiments the delay between stopping in the reward zone and receiving a reward was 0 ms throughout the experiment. In these experiments the minimum number of days to progress from stage 1 to stage 2 was 7.

Behavioral training. On each training day each mouse was removed from the holding room and taken to the experimental room 30 minutes prior to its allocated training time. The mice were handled for 5 - 10 minutes, weighed and then placed in a large cage or open field filled with toys and a wheel. Mice were then handled for a further 5 - 10 minutes before sessions on the virtual reality set up. Sessions lasted 30 - 40 minutes each. After training mice were handled again for 5 minutes before being placed in the large cage for a further 10 minutes. Mice were then returned to their home cages and the holding room before feeding.

For the experiments described in Figures 5 and 6 the experimenter was blind to the viral manipulation. For this, a colleague assigned each mouse in the experiment a number. Mice were then allocated training times according to their number. Typically the first mouse (mouse 1) began training between 10 and 11 am and the last no later than 7 pm. Allocation of mice was randomized so that an equal number of control and experimental mice were trained in the morning and afternoon. The experimenter was un-blinded only once behavioral training and initial histological analysis were complete. For the experiment described in Figure 5, a first histological analysis using a semi-quantitative scoring system was carried out before un-blinding, and a second analysis using the quantification of fluorescent images described below was carried out after un-blinding. Results from the two analyses were consistent with one another. The experiment described in Figure 6 used only the quantitative system for scoring of expression levels.

Histology. Following completion of behavioral experiments to evaluate effects of transducing stellate cells with AAV-TeLC-GFP and AAV-GFP, mice were anaesthetized with Isoflurane and given a lethal dose of sodium pentobarbital (Euthatal, Meridal Animal Health, UK). Tissues were fixed by transcardial perfusion of ice-cold phosphate buffered saline (PBS; Invitrogen) followed by 4 % paraformaldehyde (PFA; Sigma Aldrich) in 0.2 M phosphate buffer (PB; Sigma Aldrich). Brains were removed and left in 4 % PFA in 0.2 M PB overnight and then left in 30 % sucrose in PBS for two nights. Brains were stored at 4°C on a shaker and wrapped in foil at all times.

Brains were sectioned in the sagittal plane at 50 - 60 μm thickness with a freezing microtome and then floated in a solution of 0.2 % Triton X-100 (Sigma Aldrich) in PBS (PBS-T). Slices were permeabilised with 0.2 % Triton X-100 (Sigma Aldrich) in PBS (PBS-T) and blocked with 5 % normal goat serum (NGS) in PBS-T for one hour at room temperature. Sections were then

incubated for 1-2 hours in fluorescent Nissl stain (1:500; Neurotrace 640/660 or 530/610, Life Technologies) in PBS-T at room temperature. Slices were then washed in PBS-T 4 times for 15 minutes each before being mounted on polysine slides (Thermo Scientific, UK), embedded in mounting media (Mowiol) and cover-slipped. Sections from a subset of brains were tested for co-localization of reelin and calbindin with eGFP fluorescence (Supplemental Figure 4). Sections were rinsed in PBS 3 times and blocked for 1 h in 10 % NGS in PBS-T, before adding primary antibodies (anti-Reelin, raised in mouse, Millipore, 1:500; anti-Calbindin, raised in rabbit, Swant, 1:1000; in 10% NGS in PBST) and incubated overnight at 4°C. The next day, sections were washed in PBS 3 times, then incubated overnight at 4°C in secondary antibodies (goat anti-mouse Alexa-647 and goat anti-rabbit Alexa-405, both 1:500; Invitrogen in 1% NGS in PBST). They were then washed in PBS, mounted onto slides with Mowiol, and stored in the dark at 4°C until imaged.

Imaging. Imaging of labelled sections was carried out using either Nikon A1 or a Zeiss LSM800 confocal microscopes. Single plane tiled images of all the sagittal sections were taken using a EC Plan-Neofluar 5x/0.16 objective. Stacks of images containing the MEC were taken through 23 μm of the Z-axis (z-step of 3 μm) using a 20x Plan Apo VC 20x DIC N2 objective. Combinations of the laser wavelengths used were 647 nm, 555nm, 488 nm and 405 nm depending on the staining used. Images were subsequently adjusted and analysed in ImageJ. For each slice quantified, a control image was taken so that background fluorescence could be subtracted from the fluorescence measurements taken from the region of interest.

Electrophysiology experiments. We used stereotaxic surgeries as above to target AAV to the MEC of $Sim1^{Cre}$ mice. Mice were injected either with a mixture of AAV-FLEX-rev-ChR2(H134R)-mCherry and either AAV-FLEX-TeLC-GFP or AAV-FLEX-GFP. Between 2 and 4 weeks after

injections, we prepared horizontal 400 μm thick brain slices containing the hippocampal dentate gyrus. We made patch-clamp recordings from granule cells and evaluated their responses to stimulation with 488 nm wavelength light following methods we have described previously (Sürmeli et al., 2015). Slices chosen for recording showed expression of eGFP and mCherry in the middle molecular layer of the dentate gyrus. Recording electrodes had a resistance of 3-5 MΩ and contained the following intracellular solution (mM): K Gluconate 130; KCl 10, HEPES 10, MgCl2 2, EGTA 0.1, Na2ATP 2, Na2GTP 0.3 NaPhosphocreatine 10, biocytin 5.4. The external aCSF had the following composition (mM): NaCl 124, NaH2PO4 1.2, KCl 2.5, NaHCO3 25, Glucose 25, CaCl2 2, MgCl2 1. Recordings were made in current-clamp mode from cells with series resistance ≤ 30 MΩ and with bridge-balance and pipette capacitance neutralization applied.

Object exploration tasks. Experiments compared Sim1^{Cre} mice with injection of either AAV-FLEX-TeLC-GFP or AAV-FLEX-GFP into the MEC. Behavioral testing started nine days after injections. Mice were placed in an opaque plastic chamber measuring 61cm x 61cm x 60cm, surrounded by black curtains equipped with salient high contrast distal visual cues (e.g. a large yellow bin bag, a feather duster). The positions of the distal cues remained consistent throughout experiments. The chamber was cleaned prior to testing and between sessions using 70% ethanol and the bedding redistributed evenly.

Over the first 4 days the animals were habituated to the testing box. Mice then received 4 testing sessions over 4 days: two object recognition sessions, and two object-location sessions. A session consisted of three 5 min exploration phases separated by 3 min

intervals: a habituation phase, a sample phase and a test phase. For each phase, the mouse was placed into the test box, and allowed to explore. Between phases the mouse was removed from the test box and placed in an opaque container while the floor and walls of the box and objects were cleaned with 70% ethanol and the box prepared for the next phase. At the end of a testing session the mouse was placed back into its home cage, and the test box and objects cleaned and prepared for the next mouse.

The phases of the object recognition task were structured in the following way. Habituation phase: the box contained no objects. Sample phase: the box contained 2 copies of the same novel object. Test phase: one object was replaced with a third identical copy, whilst the other was replaced with a novel object. The phases of the object location task were structured in the following way. Habituation phase: the box contained no objects. Sample phase: the box contained 2 copies of the same novel object. Test phase: one object was moved to a novel location. To ensure that changes in behavior were not a result of preference for a certain object or side of the arena, the location and choice of the objects were counterbalanced between treatment groups. For both tasks, the time spent exploring each object in the sample and tests phases was measured.

QUANTIFICATION AND STATISTICAL ANALYSES

Quantification of virus expression. Quantification of virus expression was carried out in FIJI. Confocal images were opened using the Bio-Formats package (Linkert et al., 2010). For each

hemisphere in each mouse, quantification was performed in 7 sagittal slices located 3 to 3.72 mm from the midline. First, using the image of the slice labeled with Neurotrace, the length of the MEC was outlined using the freehand tool in FIJI. The midpoint of the line was used to split the MEC into dorsal and ventral sections. Layer 2 of the MEC in the dorsal and ventral sections was then selected using the freehand tool. The mean pixel intensity of fluorescence in the GFP channel, which identifies virally transduced neurons, was then calculated for dorsal and ventral layer 2 using FIJIs measurement tool. This was carried out for each of the dorsal and ventral segments in each slice.

Expression of GFP outside of layer 2 was rare, but in some animals occasional labeled cell bodies were observed in layer 5a, which was identified on the basis of Neurotrace labeling. Because GFP expression in layer 5a was sparse, and to avoid contamination from axons of L2SCs, expression was quantified by counting the number of cell bodies expressing virus. Labeled cells were counted in each slice along for each hemisphere in each mouse

Analysis of virtual reality behaviours. Data collection, analysis and presentation was performed using custom scripts written in python 3.5 (www.python.org) using Numpy v1.8.1, Scipy v0.11.0b1 and Matplotlib v1.5.1 packages. Scripts were written using Spyder 2.3 (www.pythonhosted.org/spyder). For each trial the trial type, elapsed time, track location and running speed averaged over the previous 200 ms were recorded at approximately 60 Hz as outputs from Blender. Stopping locations were recorded as locations on the track at which the velocity was < 0.7 cm/s and that were > 1 cm from a previously recorded stop. For analysis of the location of stops the track was divided into 20 bins, and the mean number of stops calculated for each bin. For experiments using longer tracks the bin size remained at 10 cm and so the number of bins was increased.

To facilitate comparison between animals, z-scored stopping probabilities were calculated as follows. First, the stop times for a given session were shuffled. For a given animal on a given day we sampled 100 trials with replacement. We considered the track as a circle and added an angle drawn from a uniform random distribution to each set of stop locations for each trial. This procedure was repeated 1000 times to generate a shuffled data set. The shuffled data set was then used to calculate means and standard deviations for the probability of stopping in each bin. Z-scored stopping probabilities were then calculated for each bin such that z score = (Mean Stops - Mean Shuffled Stops)/(Shuffled STD).

A number of additional measures of general task performance were calculated. These included: the number of trials completed per training session; the number of rewards obtained per training day; the fraction of rewarded trial; the days to meet the criteria to graduate to stage two of the experiment.

For the experiment described in Figure 4, one of the mice in the GFP group became unwell on day 17 and at this point excluded from the experiment. Data from this mouse before this point were included in the reported analyses.

Electrophysiology analysis. Electrophysiology data were analyzed using IGORpro (Wavemetrics). Amplitudes of individual light-evoked EPSPs were measured and then the mean calculated for each neuron.

Analysis of object behaviors. For each task, object exploration was scored manually using the 'Multitimer' scoring system. An exploration was recorded when the mouse's nose was within 1 cm of the object and pointing directly at it (Vogel-Ciernia and Wood, 2014). Manual scores were confirmed by repeating the scoring using AnyMaze (http://www.anymaze.co.uk/) on video recordings of the mouse's exploration. To ensure results were not skewed by animals that did not explore, exclusion criteria was introduced. Animals were excluded from the dataset if they failed to explore an individual object for more than 5 seconds or all objects for more than 15 seconds during training phases, or if they failed to explore both objects for a total of 10 seconds in the test session.

To determine whether mice have a preference for a specific object (or object location) a discrimination index was calculated for each session. The discrimination index was defined as the difference between the exploration time of the novel object (or novel object location) and the exploration time of the familiar object, divided by the total exploration time. A score greater than zero indicates preference for the novel object or object location.

Statistical analysis. Statistical analysis was performed in R v3.30 (R Core Team, 2014). Scripts were written and run using RStudio 0.99.902 (RStudio Team, 2015; http://www.rstudio.com). Details of data distributions and tests are given in the main text and figures. When a measure was obtained repeatedly from the same animal, the mean for that animal was used for population level analyses unless indicated otherwise. Linear mixed effect models (LMEs) were

fit using lme 4 1.1-12 (Bates et al., 2015). Animal identity was included in models as a random effect and the variable of interest as a fixed effect. To evaluate significance of effects using LMEs, the model without the variable of interest (a reduced/null model) was compared to the model with the variable of interest using a likelihood ratio test. Because for experiments comparing effects of expression of GFP with TeLC-GFP (Figures 3 and 4) the distribution of the data appeared clearly non-normal, for analysis of these experiments we used robust statistical methods to compare groups (Wilcox, 2016). These were implemented in R using the packages WRS (https://github.com/nicebread/WRS) and WRS2 (v0.9-2 from cran.r-project.org). Comparisons of groups used the percentile bootstrap method. For independent groups differences between medians were evaluated using the R function medpb2 (in WRS2). For dependent groups the bootdpci function (in WRS) was used to compare 20% trimmed means. Results are reported using 10^5 bootstrap samples. Linear regression was performed using a least squares method that allows heteroscedasticity, implemented in the R function olshc4 (WRS package), with slopes compared using the R function ols2ci (WRS package). For multiple comparisons within an experiment reported p values were adjusted by the Benjamani and Hockberg method using the R function p.adjust.

DATA AND SOFTWARE AVAILABILITY

Data and code to reproduce the analyses reported in the paper will be made available at the time of publication via the University of Edinburgh DataShare repository (http://datashare.is.ed.ac.uk/). Analysis code will be made available via the Nolan Lab GitHub repository (https://github.com/orgs/MattNolanLab/).

Supplemental Movie 1 (SupplementalMovie.mp4). Relates to Figure 1.

The video shows a trained mouse performing four consecutive beaconed trials followed by a non-beaconed trial.

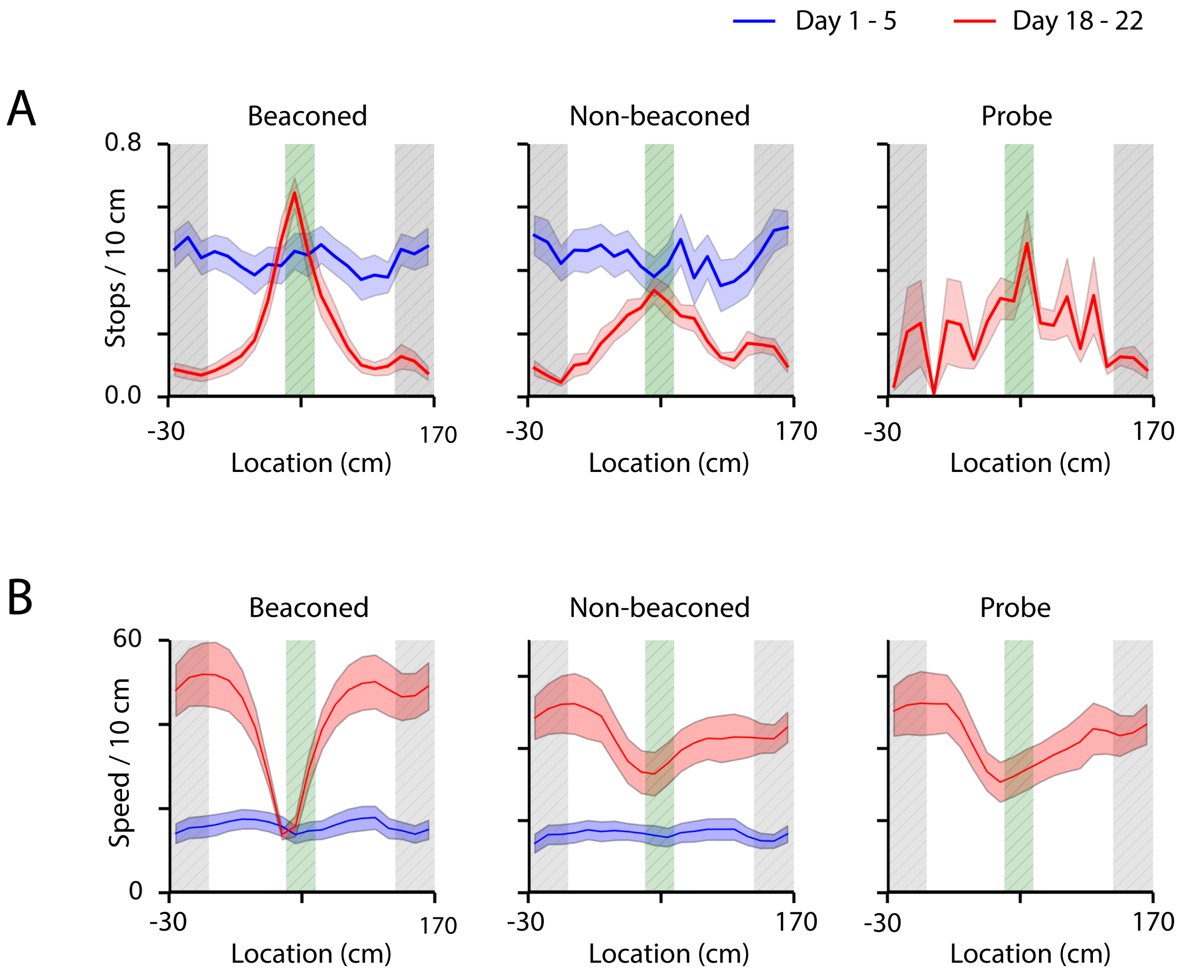

Supplemental Figure 1. Quantification of performance in a virtual location estimation task. Relates to Figure 1.

(A-B) Mean number of stops per 10 cm bin (A) and running speed (B) plotted as a function of location for beaconed, non-beaconed and probe trials on days 1-5 (blue) and 18-22 (red) of training. Shaded regions indicate the standard error of the mean.

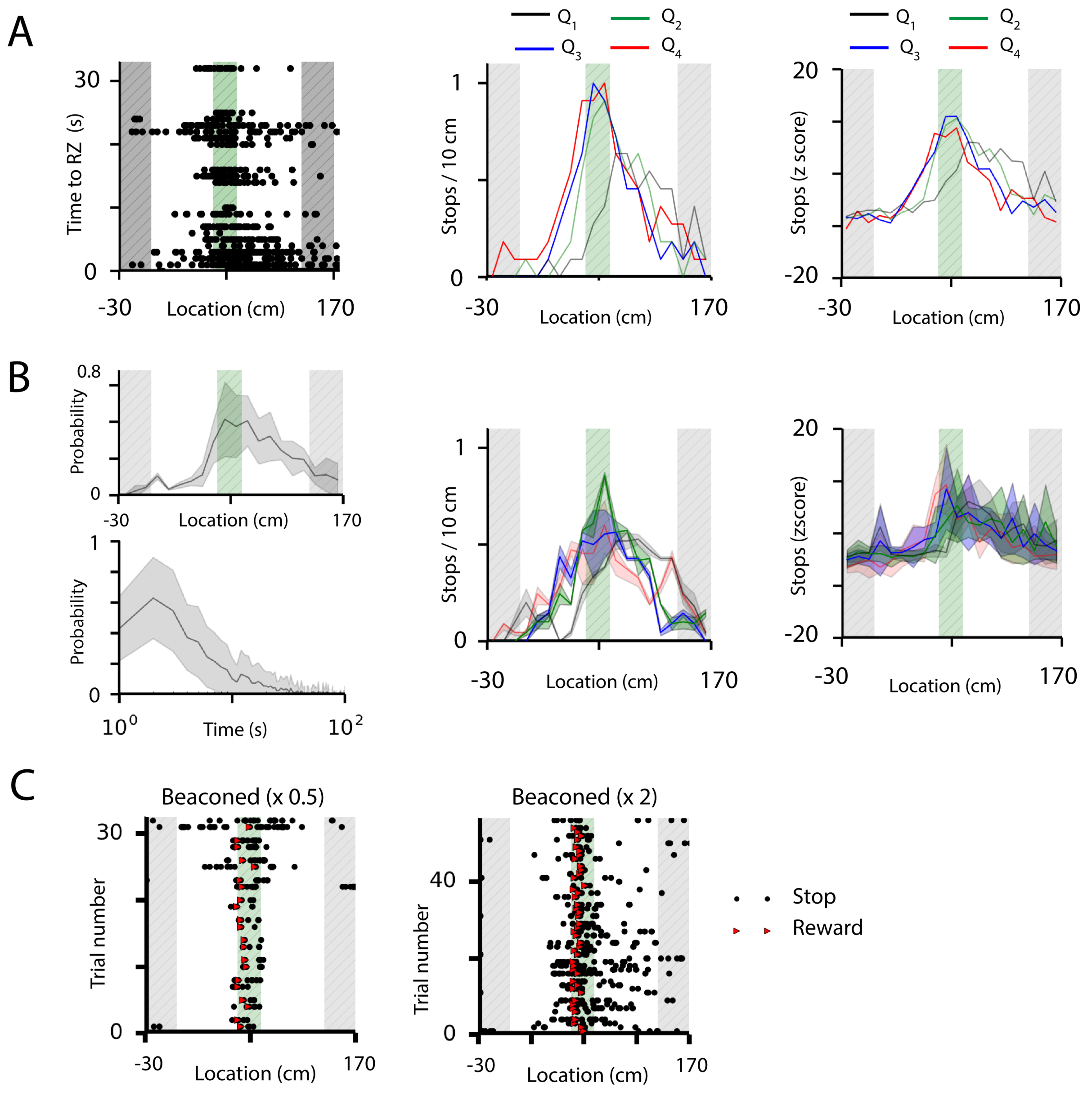

Supplemental Figure 2. Dependence of task performance on running speed and track gain. Relates to Figure 2.

A. Example plot for a single mouse of stops as a function of location with trials ordered on the y-axis according to time taken to reach the reward zone (left). The similar distribution of stop locations is consistent with use of a path integration mechanism rather than estimation of a fixed time interval. The number of stops in each 10 cm bin

(middle) and the z scored probability of stopping (right) are plotted for each quartile of the data sorted according to time taken to reach the reward zone, e.g. Q1 is the fastest 25% of trials, Q2 the next fastest, etc.

B. The distribution of stop locations (upper left) and stop times (lower left) for probe trials from all mice. Note that because the stop times were highly skewed they are plotted on a logarithmic axis. Consistent with stopping location being determined by a path integration mechanism, rather than a time counting mechanism, the mean coefficient of variation for stop times (1.18 ± 0.18) was substantially higher than for stop locations (0.45 ± 0.06; p = 0.010, paired t-test, df = 4, t = -4.6). In support of this interpretation, the group averaged time-ordered quartiles of mean stop probabilities (middle) and z scored stopping probabilities (right) did not appear to vary systematically (these panels show group data corresponding to the example data in A).

C. Examples of stopping locations on beaconed trials for the mice for which data on gain change trials is shown in Figure 2.

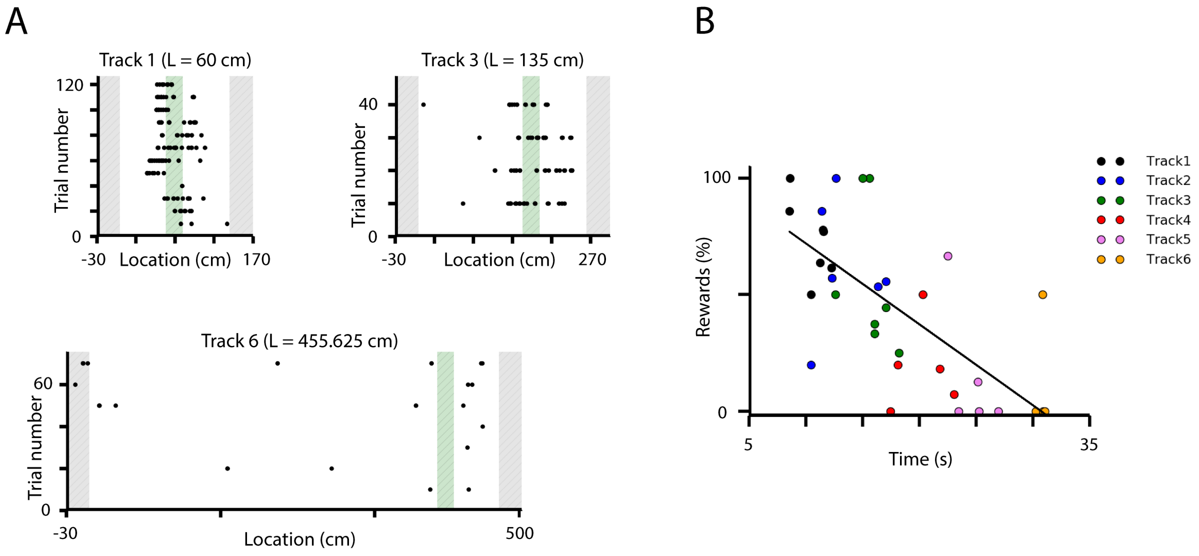

Supplemental Figure 3. Location estimates become inaccurate with increasing distance and time. Relates to Figure 3.

A. Examples of stopping locations on probe trials on tracks increasing in length. Note stop locations are centered in the reward zone on shorter tracks and become inaccurate with increasing length.

B. Percentage of correct probe trials as a function of average time to reach the reward zone for each animal on each track. Performance on different tracks is indicated by the colors. The line shows results of linear regression ($p = 4.6 \times 10^{-6}$ and $r = -0.70$).

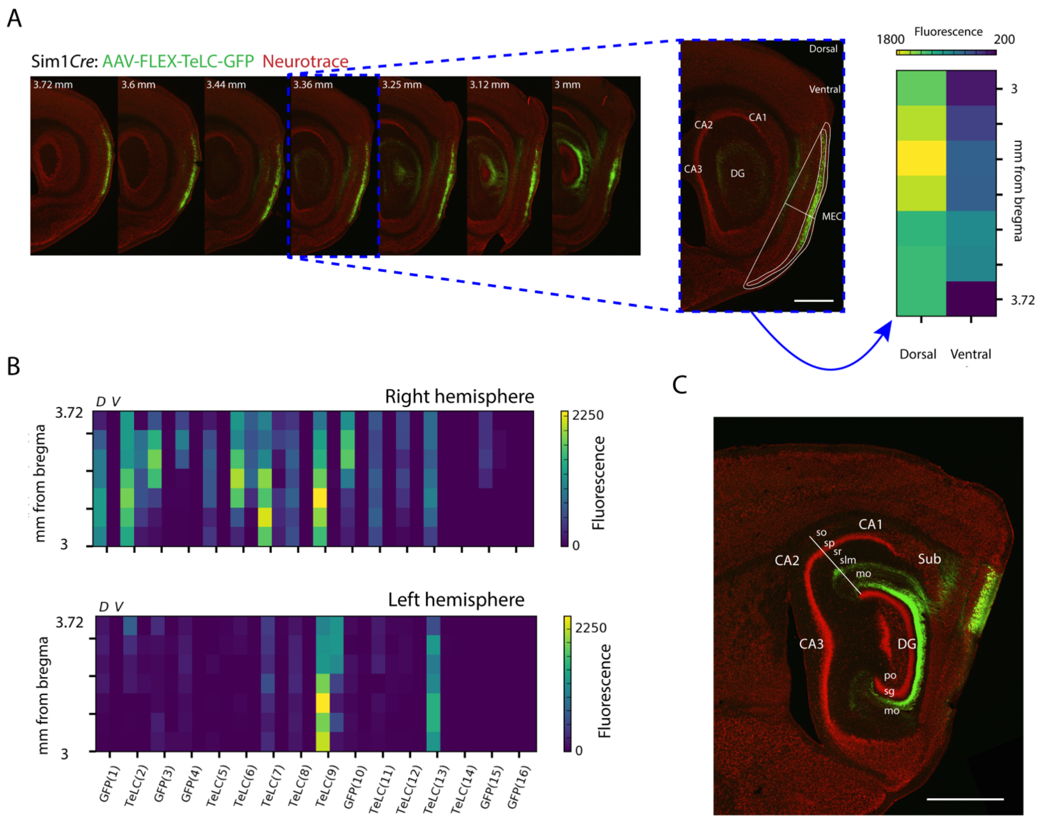

Supplemental Figure 4. Quantification of viral expression in layer 2 of the MEC. Relates to Figure 5.

A. Examples of a series of sagittal brain sections (lateral to medial) used to quantify TeLC-GFP / GFP expression (green). For each section a region of interest corresponding to layer 2 was drawn and fluorescence levels automatically calculated.

B. Quantification of TeLC-GFP / GFP expression for all animals. Height on the y-axis corresponds to the medio-lateral position of the section from which fluorescence was quantified. Dorsal (D) and ventral (V) expression levels are show separately for each animal.

(C) Expression throughout the hippocampal region. Note that labeling is present in the terminal fields of stellate cells in the dentate gyrus. Labeling is absent from CA1

indicating that entorhinal pyramidal cells do not express the virally delivered transgenes.

Scale bar is 1 mm.

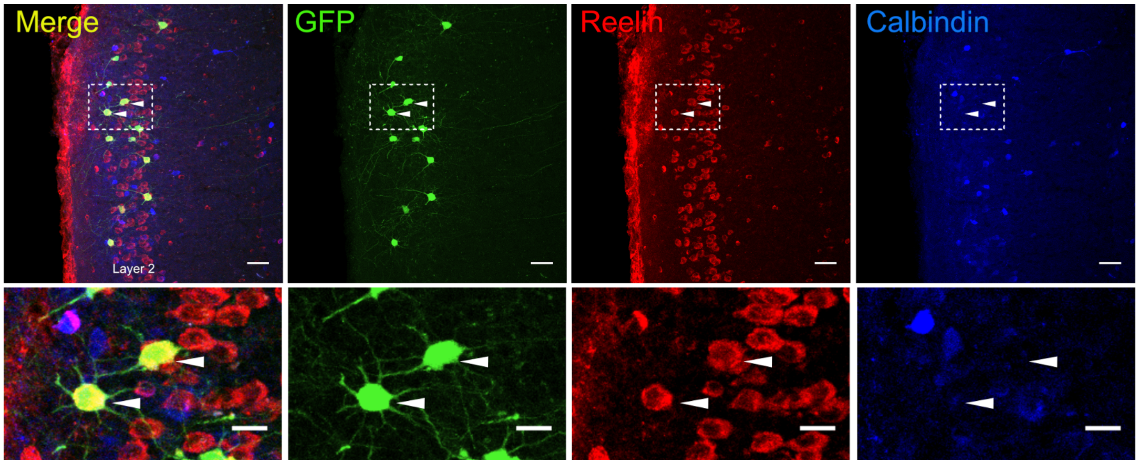

Supplemental Figure 5. Expression of TeLC is restricted to L2SCs. Relates to Figure 5.

Examples sagittal section containing MEC from a Sim1^{Cre} mouse following injection of AAV-FLEX-TeLC-eGFP. The eGFP signal (green) is present in layer 2 neurons labeled with antibodies against the stellate cell marker reelin (red), but not neurons labeled with antibodies against the pyramidal cell marker calbindin (blue). We find similar labeling in all mice tested (n = 3). Selective Cre-dependent expression of transgenes in L2SCs is consistent with previous analysis of Sim1^{Cre} mice (Sürmeli et al., 2015). Scale bars for upper panels are 50 μm and for lower panels are 20 μm.

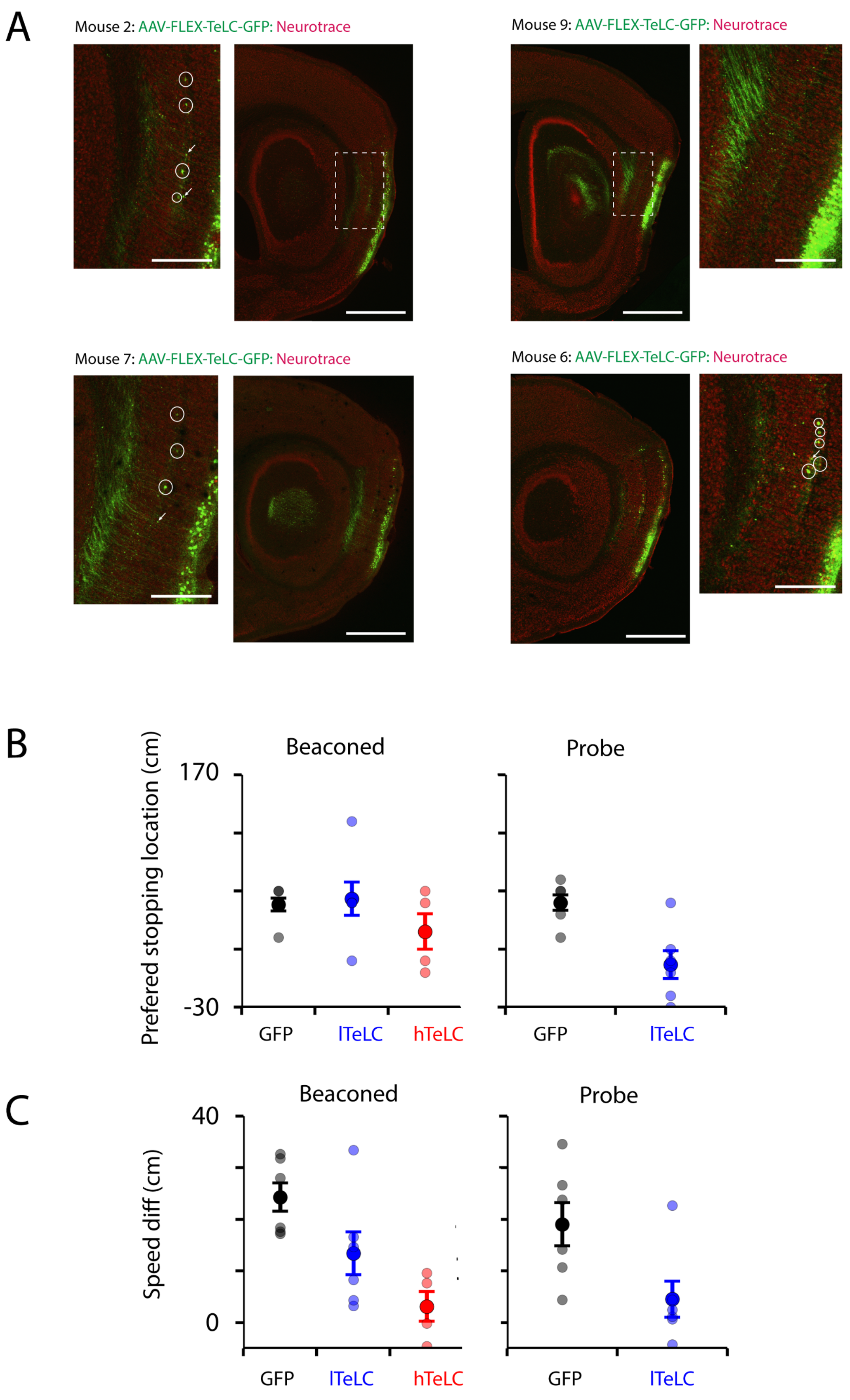

A
Mouse 2: AAV-FLEX-TeLC-GFP: Neurotrace
Mouse 9: AAV-FLEX-TeLC-GFP: Neurotrace
Mouse 7: AAV-FLEX-TeLC-GFP: Neurotrace
Mouse 6: AAV-FLEX-TeLC-GFP: Neurotrace
B
Beaconed
Probe
Prefered stopping location (cm)
170
-30
GFP
lTeLC
hTeLC
C
Beaconed
Probe
Speed diff (cm)
40
0
GFP
lTeLC
hTeLC

Supplemental Figure 6. TeLC-GFP expression in L5a and additional quantification of TeLC effects on location estimation. Relates to Figure 5.

(A) Sagittal sections from the brains of the four mice with highest expression of TeLC-eGFP. Each section is the one with the highest number of labeled cells (highlighted by circles) in layer 5a for that mouse. There were sufficiently few labeled cells in L5a that the total number across all sections could be counted (mouse 2: 16; mouse 6: 6; mouse 7: 10; mouse 9: 0). All of these mice, including the one without any detectable cells in L5a, failed to reach the performance threshold for graduate to the probe trial phase of the experiment. Mice with lower expression of TeLC-eGFP in L2 did not have detectable expression in L5a.

(B-C) Mean location of the most frequent binned stopping location (B) and mean difference between running speed at the start of the track and on entrance to the reward zone (C) during beaconed trials (left) and probe trials (centre). For each measure the beaconed and probe trials for mice in the lTeLC and eGFP groups are also compared directly (right). For the preferred stopping location neither the lTeLC or hTeLC groups differed from the GFP group (lTeLC: p = 0.78, percentile bootstrap corrected for multiple comparisons, test statistic = 0, 95% confidence interval [-3.5, 3]; hTeLC: p = 0.74, test statistic = 2.5, 95% confidence interval [-1, 6.5]), while lTeLC and GFP groups differed significantly during probe trials (p = 0.007, test statistic = 6, 95% confidence interval [2, 9]). For the difference in running speeds the hTeLC groups differed from the GFP group (p = 0.0, percentile bootstrap corrected for multiple comparisons, test statistic = 19.6, 95% confidence interval [9.0, 32.7]), but the lTeLC group did not (hTeLC: p = 0.11, test

statistic = 11.72, 95% confidence interval [-2.2, 25.6]), while lTeLC and GFP groups differed significantly during probe trials ($p = 0.03$, test statistic = 17.2, 95% confidence interval [1.6, 28.8]). For both measures performance of the lTeLC was impaired on probe compared with beaconed trials (preferred location: $p = 0.0053$, percentile bootstrap; speed difference $p = 0.0$).

REFERENCES

Bates, D., Machler, M., Bolker, B.M., and Walker, S.C. (2015). Fitting Linear Mixed-Effects Models Using lme4. J Stat Softw *67*, 1-48.

Linkert, M., Rueden, C.T., Allan, C., Burel, J.M., Moore, W., Patterson, A., Loranger, B., Moore, J., Neves, C., Macdonald, D.*, et al.* (2010). Metadata matters: access to image data in the real world. J Cell Biol *189*, 777-782.

McClure, C., Cole, K.L., Wulff, P., Klugmann, M., and Murray, A.J. (2011). Production and titering of recombinant adeno-associated viral vectors. J Vis Exp, e3348.

Murray, A.J., Sauer, J.F., Riedel, G., McClure, C., Ansel, L., Cheyne, L., Bartos, M., Wisden, W., and Wulff, P. (2011). Parvalbumin-positive CA1 interneurons are required for spatial working but not for reference memory. Nat Neurosci *14*, 297-299.

Sürmeli, G., Marcu, D.-C., McClure, C., Garden, D.L.F., Pastoll, H., and Nolan, M.F. (2015). Molecularly defined circuitry reveals input-output segregation in deep layers of the medial entorhinal cortex. Neuron *88*, 1040-1053.

Vogel-Ciernia, A., and Wood, M.A. (2014). Examining object location and object recognition memory in mice. Curr Protoc Neurosci *69*, 8 31 31-17.

Wilcox, R.R. (2016). Introduction to robust estimation and hypothesis testing, 4th edition. edn (Waltham, MA: Elsevier).
